# Design, Synthesis, and Biological Evaluation of 2-Hydroxy-4-phenylthiophene-3-carbonitrile as PD-L1 Antagonist and Its Comparison to Available Small Molecular PD-L1 Inhibitors

**DOI:** 10.1021/acs.jmedchem.3c00254

**Published:** 2023-07-14

**Authors:** Marta A. Ważyńska, Roberto Butera, Marta Requesens, Annechien Plat, Tryfon Zarganes-Tzitzikas, Constantinos G. Neochoritis, Jacek Plewka, Lukasz Skalniak, Justyna Kocik-Krol, Bogdan Musielak, Katarzyna Magiera-Mularz, Ismael Rodriguez, Simon N. Blok, Marco de Bruyn, Hans W. Nijman, Philip H. Elsinga, Tad A. Holak, Alexander Dömling

**Affiliations:** † Department of Obstetrics and Gynecology, University Medical Center Groningen, University of Groningen, Hanzeplein 1, 9713 GZ Groningen, The Netherlands; ‡ Department of Drug Design, University of Groningen, A. Deusinglaan 1, 9713 AV Groningen, The Netherlands; § Centre for Medicines Discovery, Nuffield Department of Medicine, Alzheimer’s Research UK Oxford Drug Discovery Institute, NDM Research Building, Roosevelt Drive, OX3 7FZ Oxford, U.K.; ∥ Department of Chemistry, University of Crete, Voutes, 70013 Heraklion, Greece; ⊥ Department of Organic Chemistry, Faculty of Chemistry, Jagiellonian University, Gronostajowa 2, 30-387 Krakow, Poland; # Doctoral School of Exact and Natural Sciences, Jagiellonian University, Prof. St. Łojasiewicz St 11, 30-348 Krakow, Poland; ¶ Department of Nuclear Medicine and MolecularImaging, University Medical Center Groningen, University of Groningen, Hanzeplein 1, 9713 GZ Groningen, The Netherlands; ∇ Institute of Molecular and Translational Medicine, Faculty of Medicine and Dentistry and Czech Advanced Technology and Research Institute, Palacky University in Olomouc, Olomouc 77900, Czech Republic

## Abstract

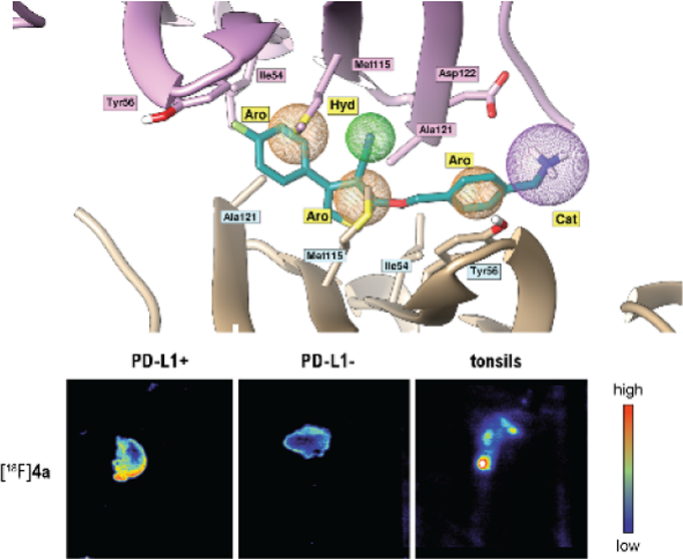

In search of a potent small molecular PD-L1 inhibitor, we designed and synthesized a compound based on a 2-hydroxy-4-phenylthiophene-3-carbonitrile moiety. Ligand’s performance was tested in vitro and compared side-by-side with a known PD-L1 antagonist with a proven bioactivity BMS1166. Subsequently, we modified both compounds to allow ^18^F labeling that could be used for PET imaging. Radiolabeling, which is used in drug development and diagnosis, was applied to investigate the properties of those ligands and test them against tissue sections with diverse expression levels of PD-L1. We confirmed biological activity toward hPD-L1 for this inhibitor, comparable with BMS1166, while holding enhanced pharmacological properties.

## Introduction

In recent decades, immunotherapy, mostly based on the PD-1/PD-L1 axis, emerged as a promising pharmacological approach in cancer treatment. Especially in lung cancer and melanoma, PD-1/PD-L1 targeting monoclonal antibodies are highly efficacious giving patients an improved chance to fight the disease.^1^ However, not every cancer patient responds to therapy and the possible side effects of antibody therapy vary from mild ones, such as skin rash and dry mouth, to more severe symptoms including hypothyroidism and autoimmunological diseases.^2^ Moreover, immunotherapy is expensive and the cost of nivolumab, a PD-1 antibody, is over 2500€ for 1 therapy cycle.^3,4^ Therefore, a search for effective treatment, which is preferably non-immunogenic, lacks adverse reactions, and is affordable is urgently needed. Those characteristics are addressed by small molecular weight inhibitors (SMIs), which are not only inexpensive to synthesize but also are usually characterized by good cell permeability and, unlike much larger antibodies, can be orally available.

The PD-1/PD-L1 axis emerged as one of the most effective immune checkpoint blockade (ICB) strategies in the last decade. Until now, only therapeutics targeting PD-1/PD-L1 and CTLA-4 have been approved by FDA. Recent studies on co-crystal structures of PD-L1 and antibodies complexes have shown that monoclonal antibodies [atezolizumab, durvalumab, or avelumab, ca. 150 kDa, binding affinity dissociation constant (p*K*_d_) of 9–10] bind by 5 complementarity-determining regions (CDRs) and those epitopes are conserved between those antibodies.^5−8^ Remarkably, smaller structures, such as PD-L1 nanobodies (KN035, ca. 15 kDa), bind just as efficiently with p*K*_d_ of 8.5, but interact with only 2 CDRs. However, it was reported that only one CDR is in fact crucial for the high affinity toward PD-L1.^8,9^ This region represents a narrow binding area yielding a possibility for a “drug miniaturization,” which will be more specific. Small molecular ligands for PD-L1 (MW < 1 kDa) can either mimic antibody binding or stabilize PD-L1 dimer. The formation of this dimer results in the inability of PD-1 to bind to PD-L1, therefore, blocking PD-1 signaling. Compounds mimicking antibodies are macrocyclic peptides.^10,11^ Other ligands follow the biphenyl structure from Bristol-Myers Squibb (BMS) patented in 2015. They are characterized by low nanomolar IC_50_ values and have been recently found to restore T-cell cytolytic function to the extent comparable with nivolumab, as well as reduce tumor growth in vivo.^11,12^ The promising preclinical testing resulted in a few of the small molecular PD-L1 inhibitors being evaluated in clinical trials, such as MAX-10181 (Maxinovel) and GS-4224 (Gilead), both ligands based on the biphenyl core.^13,14^

To study the binding properties of novel small molecular PD-L1 inhibitors, different assays can be applied. Most commonly used are PD-1/PD-L1 blockade assays employing fluorescence measurements, either on a protein level such as the homogeneous time-resolved fluorescence (HTRF) technique or on a cellular level using cell lines equipped with a luciferase reporter. However, those assays do not display all the qualities and limitations that the PD-L1 antagonist can possess. Positron emission tomography (PET), as a non-invasive imaging technique, can be used not only as a diagnostic technique but also is very useful in a drug development process, as it allows for the tracer quantification with a high sensitivity, a monitoring of the tracer distribution, metabolism in living organisms, and a subsequent clinical translation. In the case of an isotopic exchange, the molecule structure and its binding properties are usually not affected, so its biodistribution and pharmacokinetics can be easily assessed by the gamma detector systems quantitatively.

Here, we present the design, synthesis, and biological evaluation of novel 2-hydroxy-4-phenylthiophene-3-carbonitrile-based PD-L1 inhibitors and compare them with the well-known biphenyl-based PD-L1 antagonist BMS1166.

## Results

### Molecular Design

**Figure 1:**
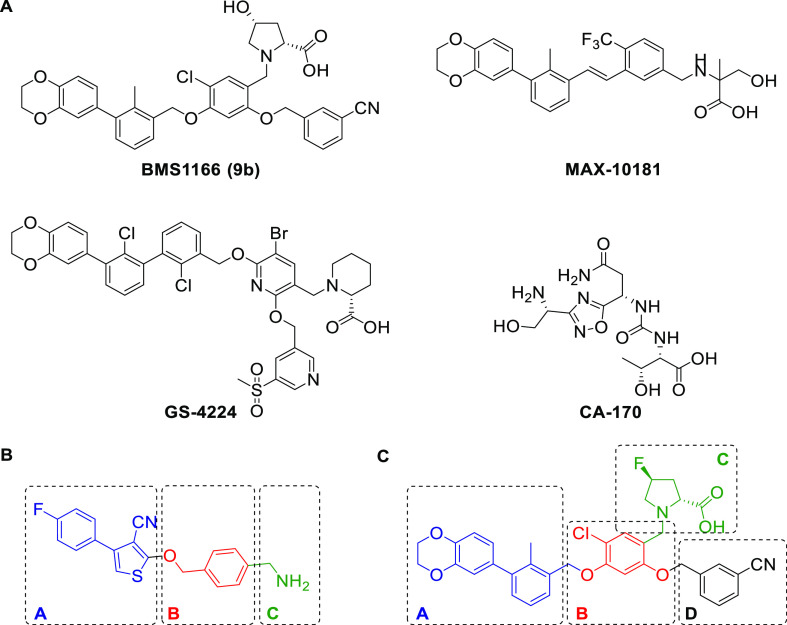
Structural design of PD-L1 inhibitors. (A) Examples of potent PD-L1 dimerizer taken from patent literature. (B) Our design of novel inhibitor 2-((4-(aminomethyl)benzyl)oxy)-4-(4-fluorophenyl)thiophene-3-carbonitrile accessible by Gewald-3CR (**4a**) and (C) structural comparison with fluorine derivative of BMS1166 (**9a**). Similar parts of the scaffolds are indicated by the different colored boxes; synthetized BMS1166 was used as a reference compound and is referred in this work as **9b**.

**Figure 2:**
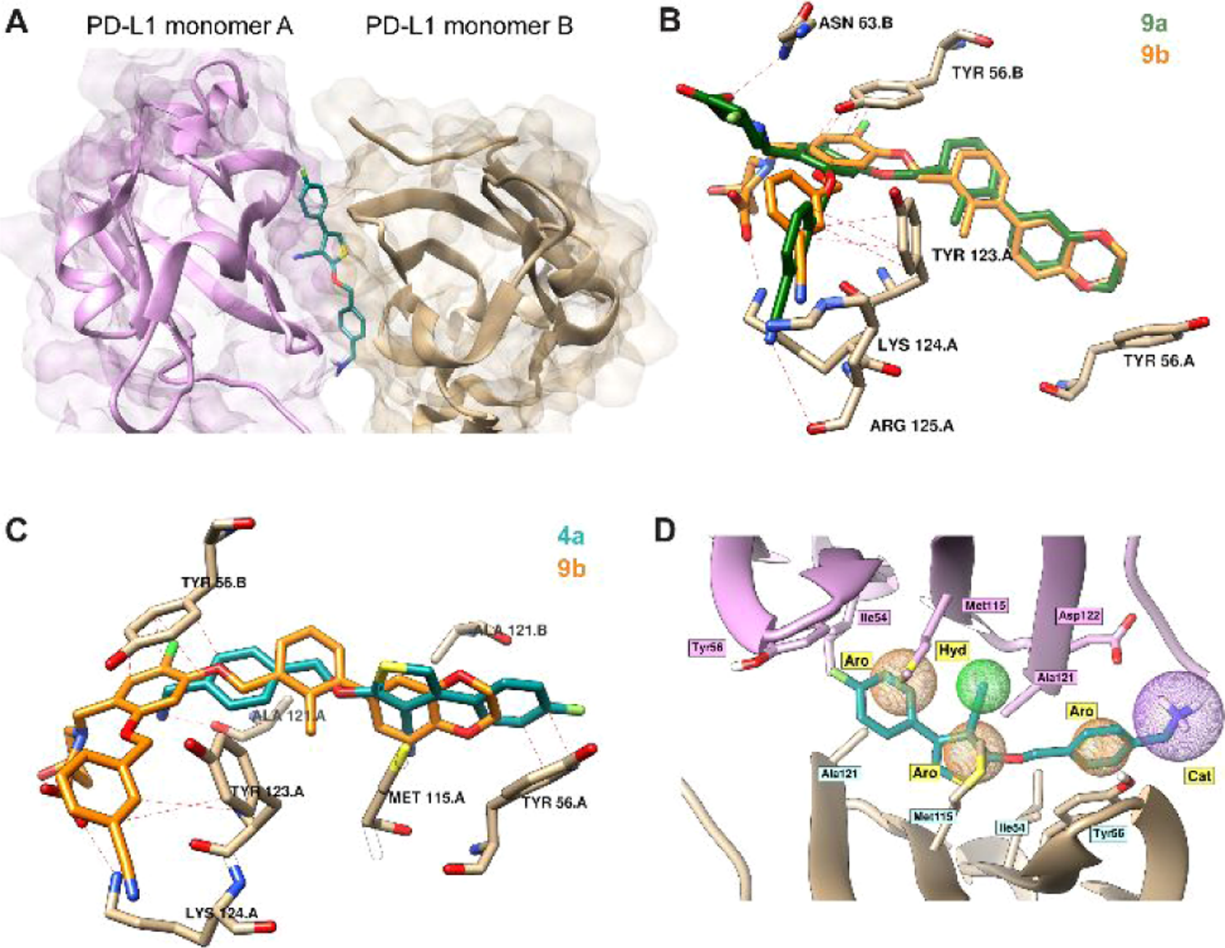
Docking of PD-L1 antagonist. (A) Docking results of an example lead compound **4a** (in green) to a PD-L1 dimer (from PDB ID: 6R3K). Blue and yellow represent two PD-L1 monomers (surface and ribbon models). (B,C) Comparison of binding modes of **4a**, **9a**, and **9b**. (B) Superimposition of **9b** from the co-crystal structure with an anticipated orientation of **9a** from the molecular docking. (C) Superimposition of **9b** from the co-crystal structure with an anticipated orientation of **4a** from the molecular docking. Additional π–π stacking interactions and hydrogen bonds are highlighted as red dashes (distance ≤ 3.3 Å),^20^ transparent amino acids denote residues anticipated to change their conformation upon binding of a superimposed ligand (**4a** and **9a**, respectively) to PD-L1 dimer. (D) Superimposition of ligand **4a** into the pharmacophore model based on the BMS1166/PD-L1 co-crystal structure (from PDB ID: 6R3K). Ph4 model shows 3 aromatic rings (orange sphere), a hydrophobic group (green sphere), and a positive charged group (purple sphere).

Following our previously introduced pharmacophore model of PD-1/PD-L1 small molecular weight antagonists, we designed a general structure based on a central thiophene ring accessible by the three-component Gewald reaction (Gewald-3CR, Figure 1B).^8,15,16^ By examining the computational docking of a novel class of small molecular inhibitors to PD-L1 and comparing it to the BMS1166 compound, we anticipated that a phenyl–thiophene moiety (Figure 1B,C, block A) resembles the biphenyl moiety from the BMS compound inducing PD-L1 dimerization and, therefore, binding in a similar manner to a site located between PD-L1 dimers, a narrow, but extended hydrophobic cavity (Figure 2A). The 3-cyano moiety helps to twist the thiopheno phenyl moiety out of plane to introduce a preformed receptor binding conformation. In the majority of other reported small molecule PD-1/PD-L1 antagonists, conformational prefix is achieved by the introduction of bulky 2-halogen substituents in the biphenyl moiety.^17,18^ Additionally, the phenyl ring following the bicyclic structure through an ether bond enabled additional strong, positioning π–π interactions at the borderline of the pocket (Figure 1B,C, block B). Our co-crystal structures of PD-L1 and BMS1166 (PDB ID: 6R3K) revealed that the amine residue (Figure 1B,C, block C) does not contribute to the binding and, therefore, can be modified.^19^ We enhanced the ligand solubility by removing the proline residue and leaving a “naked” primary amine tail acting as both H-bond donor and acceptor. Furthermore, co-crystal structures have shown that benzonitrile moiety (Figure 1C, block D) is located outside of the binding cavity, which creates strong π–π interactions with Tyr123 that might stabilize ligand inside the active center of the PD-L1 dimer. Nevertheless, another known PD-L1 antagonist—BMS202 lacks the benzonitrile moiety, while maintaining a good affinity toward the target. In our design, we decided to abstain from introducing block D and focus on a minimal version of a small molecule complying with Lipinsky’s rule of five.

**Scheme 1:**
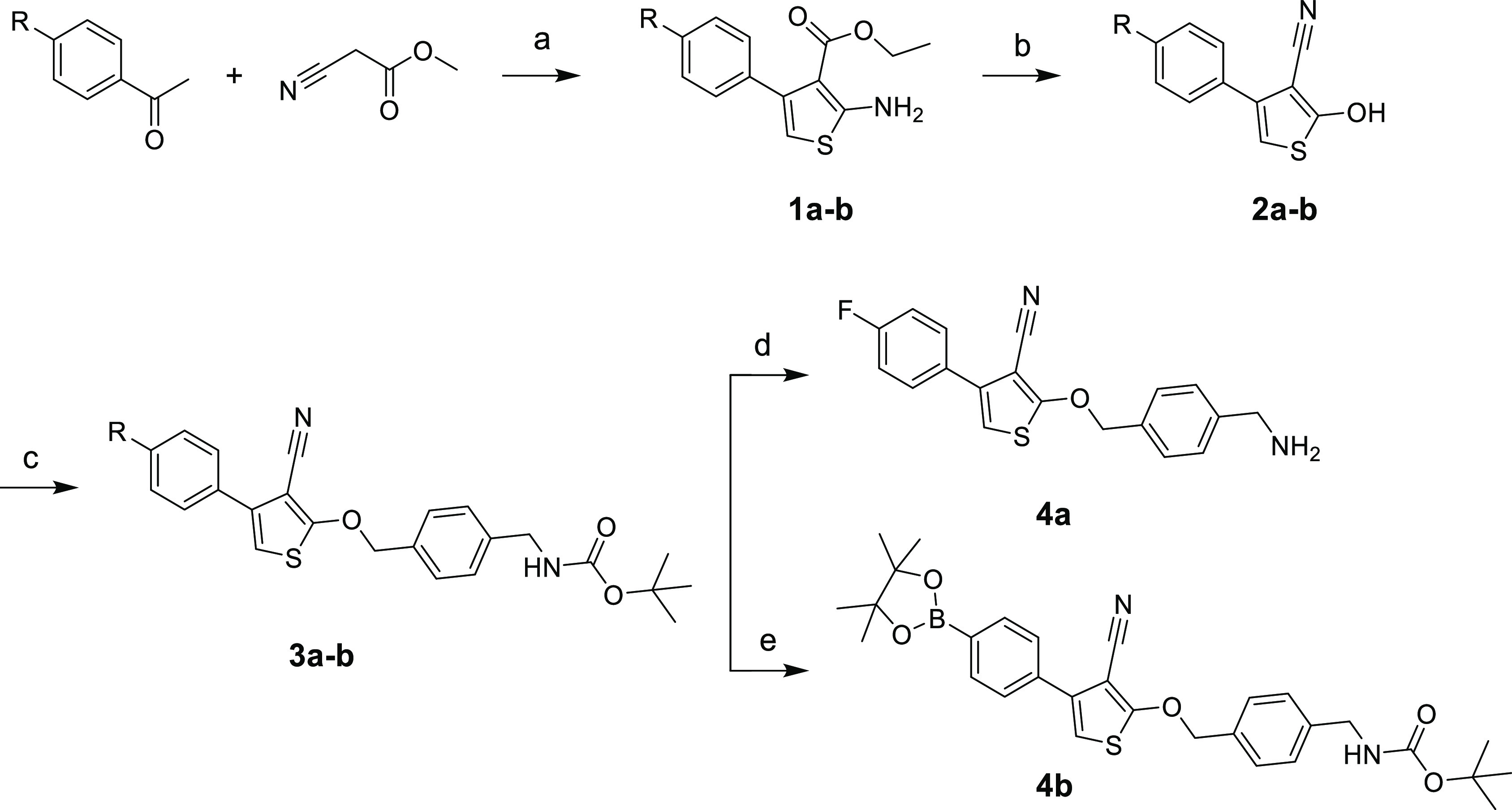
Synthetic Route of Compounds **4a–b** Reagents and conditions: (a) S_8_, DCM, 0 °C → RT, 24 h; (b) NaOEt, ethanol, reflux, 24 h; (c) K_2_CO_3_, ACN, 105 °C, 21 h; 17 h; (d) 7 N HCl in dioxane, RT, 4 h, (e) B_2_Pin_2_, Pd(dppf)Cl_2_·DCM, CH_3_COOK, dioxane, 95 °C; R = F, Br.

**Scheme 2:**
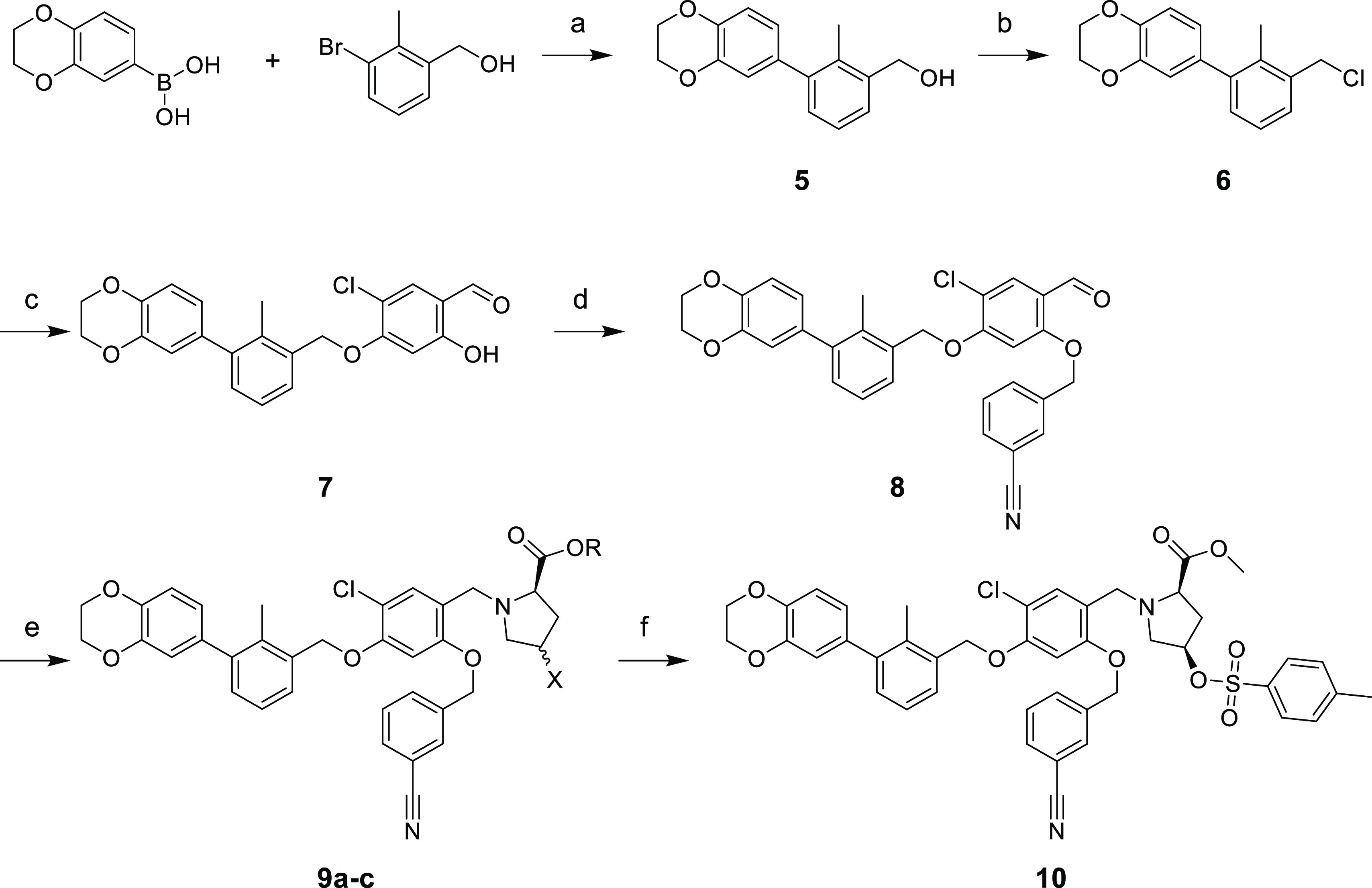
Synthetic Route of Compounds **9a–b** and **10** Reagents and conditions: (a) Pd(dppf)Cl_2_, 80 °C, 12 h; (b) SOCl_2_, DCM, 0 °C → RT, 20 h; (c) 5-chloro-2,4-dihydroxybenzaldehyde, NaHCO_3_, KI, ACN, DMF, 65 °C, 20 h; (d) K_2_CO_3_, ACN, 75 °C; (e) (2*S*,4*S*)-4-fluoropyrrolidine-2-carboxylic acid (**9a**), (2*S*,4*R*)-4-hydroxypyrrolidine-2-carboxylic acid (**9b**) or methyl (2*S*,4*R*)-4-hydroxypyrrolidine-2-carboxylate (**9c**), NaCNBH_3_ or NaBH(OAc)_3_, DMF, DCE; and (f) TosCl, Et_3_N, DCM.

Computational docking of novel ligands by the superposition onto the co-crystal structure of BMS1166 and PD-L1 dimer (PDB ID: 6R3K) as well as pharmacophore modelling of superimposed **4a** and **9b** confirmed similar binding modes of those two compounds (Figure 2). Although 2-((4-(aminomethyl)benzyl)oxy)-4-(4-fluorophenyl)thiophene-3-carbonitrile lacks a phenyl ring (Figure 1C, block D), which supports binding outside of the pocket through π–π interaction with Tyr123, its structure inside the pocket provides additional interactions. Block A of compound **4a** is involved in a strong, stabilizing π–π interaction through its phenyl group with Tyr56 and hydrophobic contacts with all surrounding hydrophobic residues. Primary amine from block C, which lays outside of the binding pocket, is likely involved in the charge–charge contact with Asp122. Computational docking allowed us to explore additional modifications to the core molecule and their effect on binding to the target. The modification most interesting for us would be an addition of fluorine that would grant the radiolabeling with fluorine-18, useful for further biological evaluation. Substitution with fluorine at the phenyl-thiophene ring of **4a** and the fluorine modification of 4-hydroxyproline of **9b** did not alter the binding mode as no major changes of the conformation were observed. We continued with the synthesis of compound derivatives utilizing reactions shown in Schemes 1 and 2.

### Ligands Synthesis

Our synthetic pathway toward the targeted compounds **4a–b**, started with the Gewald three component reaction (G-3CR), which gives us access to the 4-phenyl substituted thiophene ring.^21−25^ Indeed, *p*-substituted acetophenones (R = F for **1a** and Br for **1b**) reacted with methyl cyanoacetate and elemental sulfur to generate compounds **1a–b**, almost quantitatively, in 92 and 86% overall yields, respectively. Next, an rearrangement reaction was employed, and in the presence of sodium ethoxide, compounds **2a–b** in 30 and 52% were yielded, respectively.^21^ A Williamson ether synthesis reaction was used to couple *tert*-butyl (4-(hydroxymethyl)benzyl)carbamate in 45 and 76% for **3a–b**), followed by a quantitative Boc-deprotection using 4 M HCl in dioxane affording the fluorinated compound **4a**. Finally, for bromine-derivative **3b**, we opted to not perform a deprotection step, but performed a Miyaura borylation to obtain compound **4b** in 60% yield.

Accessing compounds **9a–b** and **10** was achieved following the procedure described below. First, a Suzuki coupling of (2,3-dihydrobenzo[*b*][1,4]dioxin-6-yl)boronic acid and 3-bromo-2-methylphenyl)methanol was accomplished to afford compound **5** in 90% yield. Then, a chlorination reaction of compound **5** was performed to afford compound **6** in quantitative yield. Subsequently, a nucleophilic substitution with 5-chloro-2,4-dihydroxybenzaldehyde was employed to obtain **7** (74% yield). An additional nucleophilic substitution involving compound **7** and 3-(chloromethyl)benzonitrile was carried out to provide quantitatively compound **8**. The final step toward **9a–c** was based on a reductive amination involving either (2*R*,4*S*)-4-fluoropyrrolidine-2-carboxylic acid (for **9a**), (2*R*,4*R*)-4-hydroxypyrrolidine-2-carboxylic acid (for **9b**), or methyl (2*R*,4*R*)-4-hydroxypyrrolidine-2-carboxylate (for **9c**) affording **9a–c** in 58–60% yield. An additional step was necessary involving compound **9c** and 4-toluenesulfonyl chloride, attaining compound **10** for further ^18^F-labeling with a good yield of 56%.

### Comparison of Ligands’ Biological Activity

**Figure 3:**
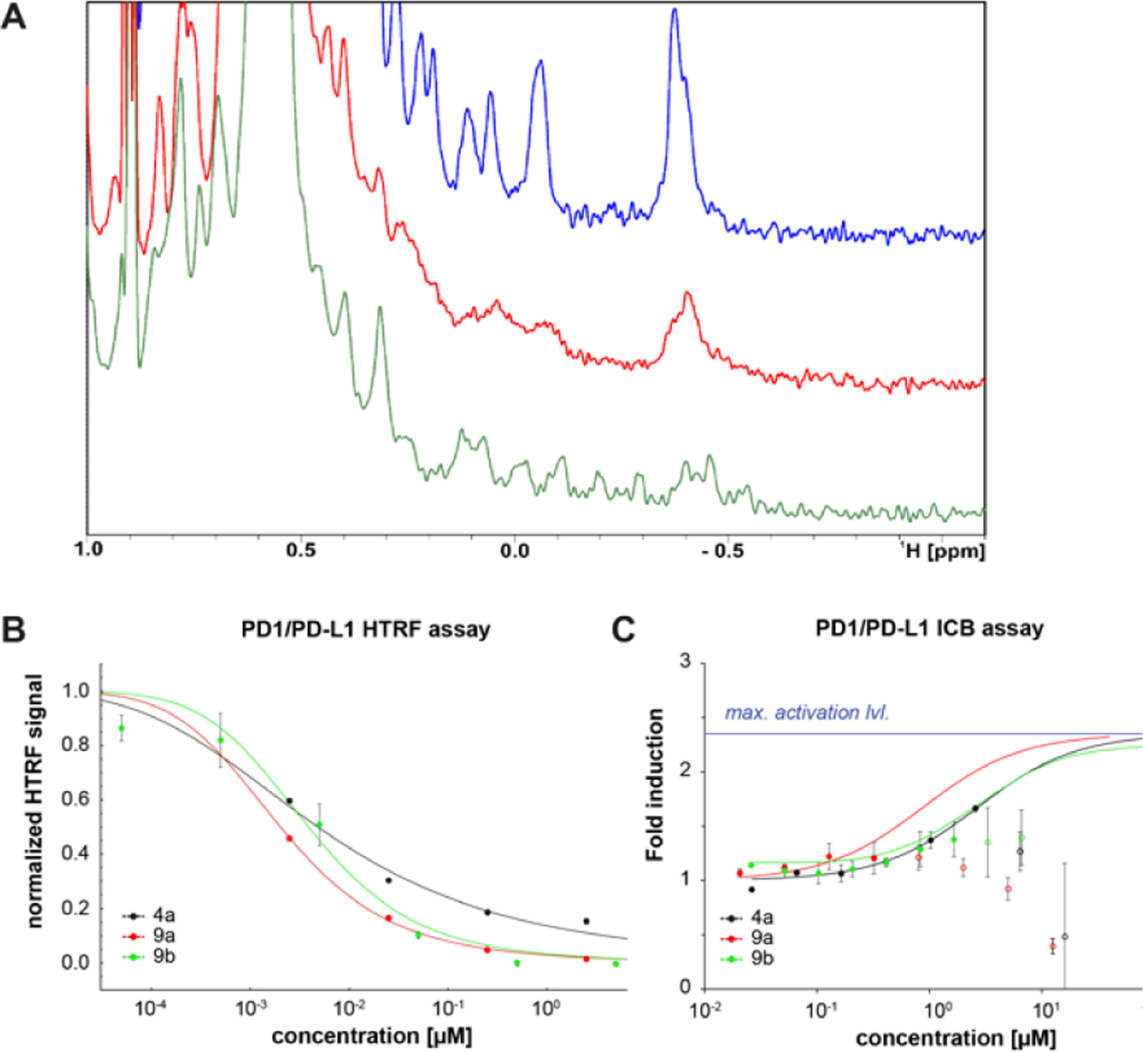
Biophysical characterization of the potency of tested compounds (A) aliphatic part of ^1^H NMR spectra of apo-PD-L1 (blue), PD-L1/**4a** (red), and PD-L1/**9b** (green) in the molar ratio 1:1. (B) IC_50_ determination using HTRF PD-1/PD-L1 assay. Measurements were done in two independent dilution series (C) PD-1/PD-L1 immune checkpoint blockade assay for **4a**, **9a**, and **9b**. The fitted function was estimated from Hill’s equation and points without a filled circle were excluded from the fitting due to high cytotoxicity.

To investigate if our synthesized analogs are capable of disrupting PD-1/PD-L1 binding, we performed our previously described nuclear magnetic resonance (NMR) assay.^16^ We collected both 1D and 2D spectra of PD-L1 apo (blue) in the presence of **4a** (red) and positive control BMS1166 (**9b**) (green) at molar ratio 1:1 (Figures 3A and S1A). Both ^1^H NMR and ^1^H–^15^N heteronuclear multiple quantum coherence (HMQC) signals show that the addition of **4a** as well as reference compound BMS1166 induces dimerization of PD-L1 that was also previously observed for the biphenyl compounds.^26^ The PD-L1 dimerization causes peaks of the ^1^H aliphatic part PD-L1 spectrum to flatten (peak at −0.4 ppm) or disappear in the case of peaks from ^1^H–^15^N HMQC spectrum. Dimerization of PD-L1, in turn, prevents its binding to the PD-1.

To determine the potency of synthesized inhibitors to disrupt the PD-1/PD-L1 complex, we used homogeneous time resolved fluorescence (HTRF), which is a well-established method for the inhibitory activity of PD-L1 assessment based on RT-FRET effect.^10,11,26,27^ In this binding assay, tagged PD-1 and PD-L1 are labelled with anti-tag reagent that generates FRET signal only when both proteins are bound (in spatial proximity). Presence of inhibitors (small molecules, proteins, and so forth) that disrupt the binding decreases the resulting signal allowing the assessment of the inhibitory activity of tested inhibitors via IC_50_ (the half maximal inhibitory concentration). Compared to the positive control BMS1166 (**9b**) IC_50_ of 3.78 nM, both tested compounds exhibit comparable inhibitory activities (**9a** 2.01 nM and **4a** 4.97 nM) proving their potency in disrupting the PD-1/PD-L1 complex (Figure 3B). Additionally, we have tested compound **4a** and **9a** for their affinities to human PD-L1. Reported values showed that **4a** binds to hPD-L1 with kD of ca. 27 nM, while **9a** with ca. 8 nM, which is consistent with low nanomolar inhibitory activities of those compounds in HTRF assay (Figure S1C).

To determine if the in vitro results translate to the cell-based assay, we performed the cellular assay, in which coculturing Jurkat reporter T cells and artificial antigen-presenting cells (aAPCs) provided the binding between PD-1 and PD-L1. The disruption of the binding between PD-1 and PD-L1 via inhibitor enhances a TCR-mediated activation of the Jurkat cells leading to the increase in the coupled luciferase signal intensity. Therefore, reporting luciferase activity can be used to track the activation status of the Jurkat T cells. In our cellular assay, tested compounds gave EC_50_ values of 2.70 μM for **4a**, 0.88 μM for **9a**, and 1.57 μM for **9b** (matching previously obtained value for the positive control).^27^ However, in higher concentrations, they exhibit cytotoxicity toward the Jurkat cell line, and the EC_50_ values, especially for **9a**, should be approached as an estimation (Figures 3C and S1B).

### Radiochemistry

For compounds **4a** and **9a** with a proven inhibitory activity toward PD-L1, we performed radiolabeling to explore other pharmacological properties, such as lipophilicity and short-term stability, as well as binding to PD-L1^+/–^ tissue sections.

**Scheme 3:**
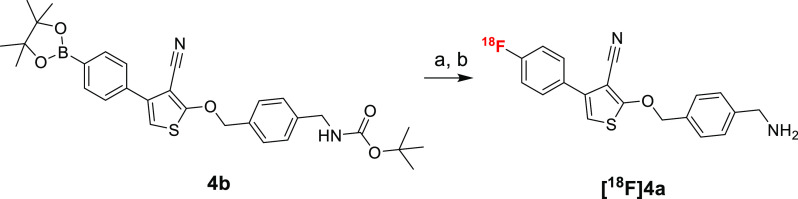
Copper-Mediated Fluorination of [^18^F]**4a** from Boronic Pinacol Ester Precursor **4b** Reagents and conditions: (a) [^18^F]F^–^, Et_4_N·HCO_3_, *n*BuOH, DMA, 110 °C, 15 min; (b) 6 N HCl_aq_ 110 °C, 10 min.

[^18^F]**4a** radiosynthesis was performed by the copper-mediated fluorination of boronate pinacol ester (**4b**) according to a procedure by Mossine et al. (Scheme 3).^28^ Optimal reaction time of 15 min was chosen based on TLC conversion measurements (54, 56, 57% at 15, 30, and 45 min, respectively, Figure S2A). Synthesis was accomplished with good radiochemical yield (up to 18% RCY) and very good molar activity (up to 138 669 GBq/mmol).

**Scheme 4:**
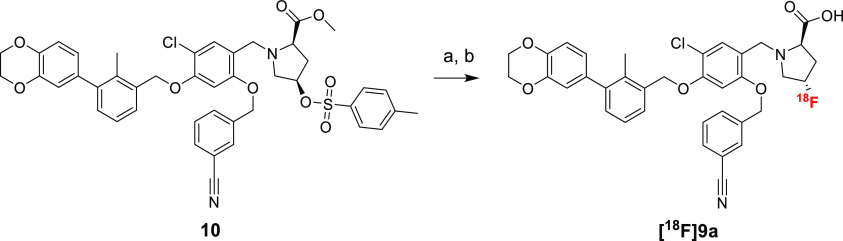
Fluorination of [^18^F]**9a** from Tosylate Precursor **10** Reagents and conditions: (a) [^18^F]F^–^, K_2_CO_3_, Kryptofix 222, DMSO, 100 °C, 10 min; (b) 1 M LiOH_aq_ MeOH, 100 °C, 10 min.

[^18^F]**9a** was accessed via nucleophilic substitution of **10** (tosylate) and subsequent esterolysis using 1 M LiOH_aq_ (Scheme 4). The usage of acetonitrile, the most common solvent for nucleophilic substitution with ^18^F, resulted in a low conversion (<3%) and was replaced by DMSO. The optimal reaction time of 10 min was chosen based on TLC conversion measurements (33, 37, 36% at 10, 20, and 35 min, respectively, Figure S2B). Two routes were explored for this radiosynthesis, with purification in between two steps (method A) and subsequent two steps performed as a one-pot reaction (method B). Method A, although resulting in a much cleaner profile of hydrolysis reaction, took twice as long and resulted in a lower radiochemical yield (ca. 180 vs 85 min, the maximal value of RCY 2 vs 5% d. c., Figure S2C). [^18^F]**9a** synthesis was accomplished by method B with moderate radiochemical yield (up to 5% RCY) and very good molar activity (up to 144 836 GBq/mmol).

Both tracers were obtained with very good radiochemical purity and molar activity as shown in Table 1 and were subjected to lipophilicity and stability measurements. Experimental lipophilicity listed in Table 1 matched predicted log *P* values (5.38 for [^18^F]**9a** and 3.7 for [^18^F]**4a**).^29^ The assay confirmed high lipophilicity of [^18^F]**9a** and moderate lipophilicity of [^18^F]**4a**. Stability in formulation solution after 4 h for both compounds maintained above 95% threshold, however, stability in human serum showed slightly higher degradation for [^18^F]**9a** than [^18^F]**4a** (minimal value 82 vs 93%, Figure S7).

**Table 1:** Comparison of properties of [^18^F]Labeled PD-L1 Tracers

tracer	[^18^F]**4a**	[^18^F]**9a**
radiochemical purity [%]	99.8 ± 0.5^b^	99.2 ± 1.1^c^
molar activity [GBq/mmol]	78 876 ± 84 561^b^	100 794 ± 48 716^c^
calculated log *P* (XLOGP3^29^)	3.70	5.38
experimental log *P*^a^	3.6 ± 0.4	5.2 ± 0.6
experimental log *D*_7.4_^a^	3.9 ± 0.9	6.5 ± 0.2
stability in formulation solution after 4 h [%]^a^	97.5 ± 0.5	97.3 ± 1.1
stability in serum after 4 h [%]^a^	93.9 ± 0.5	88.7 ± 5.7

a Experiment performed in triplicate.

b *n* = 4.

c *n* = 6.

### Autoradiography with PDL1^+/–^ Tissue Sections

**Figure 4:**
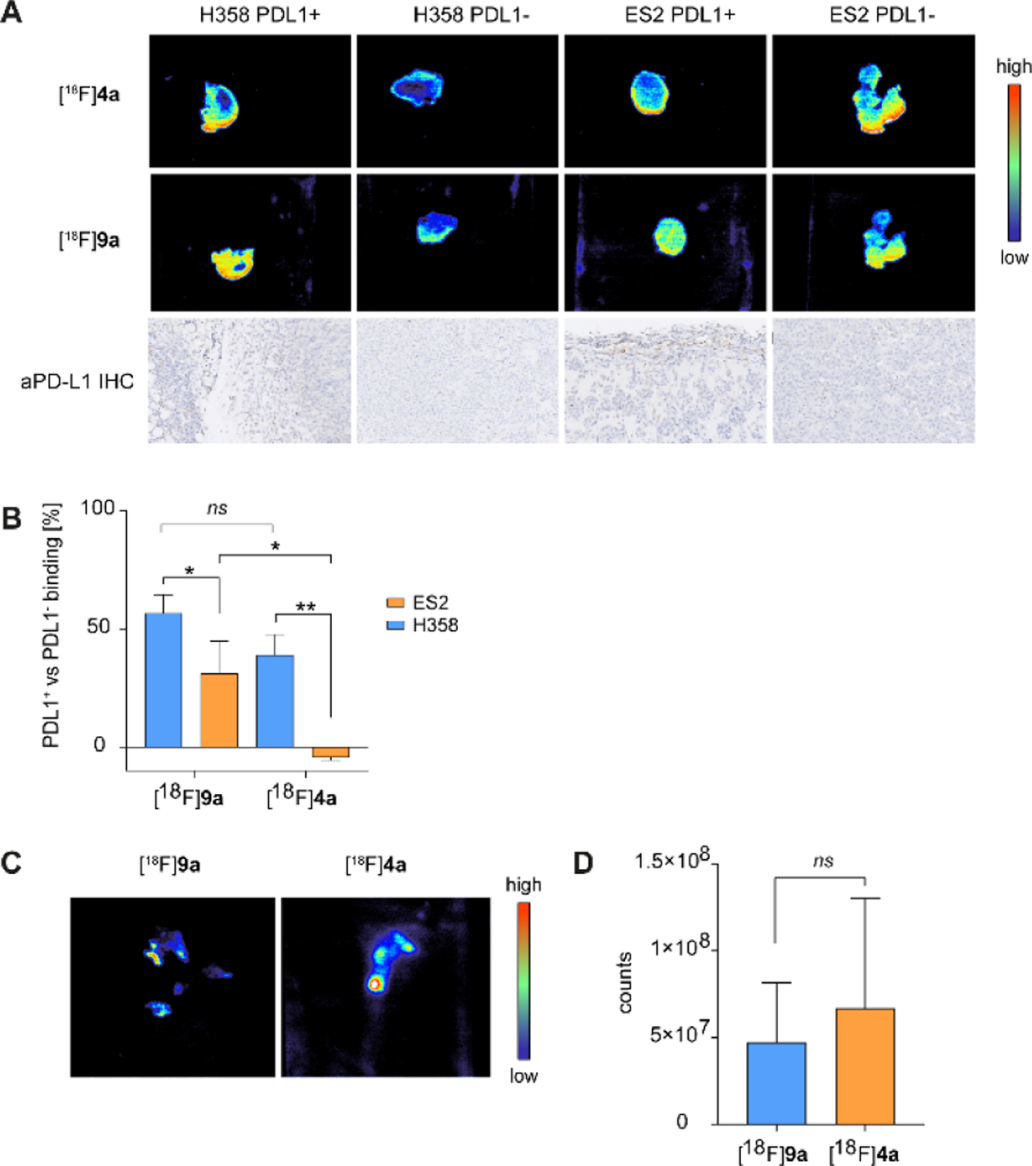
Autoradiography with PD-L1^+/–^ (H358 and ES2) tumor for [^18^F]**4a** and [^18^F]**9a**. (A) Images of tumor tissue slides incubated with [^18^F]**4a** and [^18^F]**9a** and comparison with immunohistochemistry staining with PD-L1 antibody. (B–D) Autoradiography quantification and tracer comparison (B). Autoradiography with human tonsils for [^18^F]**4a** and [^18^F]**9a** (C). Autoradiography quantification and tracer comparison (D); experiments performed in triplicate and groups compared with Mann–Whitney test (nonparametric, *p < 0.05, **p < 0.051), colorful scale signifies color coding from low to high tracer uptake.

Next, we explored the binding properties of the synthesized compounds in a human tissue setting. We selected available tumor tissue sections—ES2 (ovarian carcinoma) and H358 (lung adenocarcinoma). Tumors were prepared in two versions—wild type, expressing PD-L1 and PD-L1 knock-out, as shown in intra- and extracellular measurements of the above cell lines (Figure S8). We noted a 40–55% higher uptake of both, [^18^F]**4a** and [^18^F]**9a** compounds, in PD-L1^+^ H358 tumors than in wild-type counterparts. Uptake correlated with PD-L1 expression, which was confirmed by immunohistochemistry staining (Figure 4A). Interestingly, the uptake was localized mostly on the peripheral part of the tumor slide, similar to immunohistochemistry staining. For H358, both tracers performed similarly well (Figure 4B). Unfortunately, [^18^F]**4a** failed to discriminate between wild-type and knock-out for ES2 slides. Elevated, unspecific uptake in PD-L1^–^ tumors could be explained by the high lipophilicity of compounds and nature of paraffin-embedded tissues, which often result in upregulated unspecific uptake. Afterward, to eliminate potential issues with paraffin embedding, we examined binding to snap-frozen human tonsils. Tonsils are known to overexpress PD-L1, especially in structures called germinal centers.^30−32^ We were able to achieve a similar imaging pattern with both of the tracers. Quantification confirmed no difference in binding between those two molecules (Figure 4C,D).

## Discussion and Conclusions

Immune checkpoint blockade, especially the PD-1/PD-L1 axis, has been widely popularized due to its phenomenal results in cancer treatment. Although the PD-1/PD-L1 role in tumor immunology and its downstream signaling has been extensively studied, the PD-L1 blockade and its effect on PD-1 are not fully comprehended. Particularly, the mode of action for small molecular, non-peptidic PD-L1 antagonists is not fully understood. Only recently, few studies investigated the effects of small molecular weight PD-1/PD-L1 antagonists.^33−38^ This research revealed how vastly different outcomes can be achieved for PD-L1 inhibitors using different biological assays. Notably, Aurigene-1 (a close derivative of clinical trial compound CA-170) exhibits nanomolar potency in the splenocyte recovery in an assay that was used as a scouting method for PD-1/PD-L1 inhibitors.^33^ However, Aurigene-1 does not show binding in SPR binding assay, nor does it show activity in ELISA biochemical assay, and it does not induce IL-2 production.^35^ Moreover, our research proved that CA-170 is not a direct binder to either human or murine PD-L1 by means of NMR, HTRF, and cellular assays.^29^ Additionally, BMS small molecular compounds, while being able to disrupt the PD-1/PD-L1 axis in Jurkat cells modified with NFAT promoter, display strong cytotoxicity at higher concentrations.^35−38^ On account of those conflicting characteristics, further understanding of the mode of action is necessary for a rational design and the discovery of novel PD-L1 inhibitors.

In this work, we report the design, synthesis, and biological evaluation of an exemplary ligand from the novel, 2-hydroxy-4-phenylthiophene-3-carbonitrile-based class of compounds. Those compounds act as PD-L1 antagonists, and their structural analysis based on molecular docking reveals similarities to small molecular PD-L1 inhibitors developed by Bristol-Myers Squibb. We confirmed their biological activity not only in computational docking and biophysical studies but also in various in vitro assays. We have proven that tested compounds structurally interact with hPD-L1 as evident by examining the shifts in both protein-based 1D and 2D NMR spectra upon compound addition. Furthermore, we assessed the ability of those compounds to disrupt PD-1/PD-L1 binding in HTRF assay to be low single digit nanomolar. Additionally, the cell-based PD-1/PD-L1 immune checkpoint blockade assay has confirmed the activity of all compounds. We observed similar binding properties of our novel scaffold to known and potent PD-L1 antagonist—BMS1166. We were able to radiolabel 2-hydroxy-4-phenylthiophene-3-carbonitrile and BMS1166-based inhibitors via copper-mediated fluorination of boronic pinacol esters and nucleophilic substitution. Tracer production was performed in moderate to good yields and afforded pure compounds with a molar activity. Radiolabeling with fluorine-18 allowed us to expand our knowledge of certain properties of those compounds, such as lipophilicity and short-term stability, as well as performed biological assays on tissues with various PD-L1 expressions. We established similar patterns of binding for the synthesized compounds to H358 cell lines (PDL1^+/–^) and human tonsils. [^18^F]**4a** was unable to bind specifically to ES2 cell lines (PDL1^+/–^), however, it showed lower background uptake than [^18^F]**9a**, which could be explained by lower lipophilicity. Additionally, better stability in human serum was established for [^18^F]**4a**. These findings suggest that [^18^F]**4a** might be a promising PD-1/PD-L1 antagonist candidate for diagnostics as a tracer or therapy as a non-radioactive compound **4a**, however further evaluation needs to be performed. To prove utility of compound **4a**, a comparative in vivo PET study with **9a** must be performed utilizing immunocompromised mouse model inoculated with human tumor cell lines used in this work. This tracer study will reveal the optimal timeline for compound distribution, preferred targeted tumor cell line (characterized by different level of PD-L1 expression), and additional organ selectivity. Due to lipophilic nature of these compounds, a preferred pathway of degradation would be a biliary excretion. Therefore, an assessment of accumulation in the liver and hepatoxicity must be evaluated. Additionally, we observed toxicity in Jurkat cells at higher concentration and in vitro toxicity study would prove essential to further strategize usefulness of these compounds. Radiolabeling could be used not only to observe compound distribution in real time in a mouse model but also determine metabolic stability and formed metabolites. The design of this novel scaffold allows for several modifications that could further improve their binding, lipophilicity, and stability. The addition of a component representing block D (Figure 1) e.g., a phenyl, could provide increased affinity and we believe derivatization of this phenyl ring with hydrophilic moieties could lower the lipophilicity and increase compound’s water stability. We foresee that the synthesis and biological assessment of compounds based on **4a** could be beneficial in finding effective PD-L1 inhibitors for diagnostic or therapeutic purposes.

## Experimental Section

### General Information

Reagents were acquired from commercial suppliers (Sigma-Aldrich, ABCR, Acros and AK Scientific) and used without any purification unless otherwise noted. Thin layer chromatography was performed on Fluka precoated silica gel plates (0.20 mm thick, particle size 25 μm). Flash chromatography was performed on a Teledyne ISCO Combiflash Rf, using RediSep Rf Normal-phase Silica Flash Columns (Silica Gel 60 Å, 230–400 mesh) and on a Reveleris X2 Flash Chromatography, using Grace Reveleris Silica flash cartridges (40, 24, 12, and 3 g). All HTRF experiments were performed using a Cisbio Bioassays Human PD1/PD-L1 biochemical binding assay. Nuclear magnetic resonance spectra were recorded on a Bruker Avance 500 spectrometer. Chemical shifts for ^1^H NMR were reported in ppm relative to TMS (δ = 0 ppm) or the corresponding solvent peak (CDCl_3_ δ = 7.26 ppm, DMSO-*d*_6_ δ = 2.50 ppm, and CD_3_OD δ = 3.31 ppm) and coupling constants were reported in hertz (Hz). The following abbreviations were used for spin multiplicity: s = singlet, bs = broad singlet, d = doublet, dd = doublet of doublets, t = triplet, ddt = doublet of doublet of triplets, q = quartet, and m = multiplet. Chemical shifts for ^13^C NMR were reported in ppm relative to the solvent peak (CDCl_3_ δ = 77.2 ppm, DMSO-*d*_6_ δ = 39.5 ppm, and CD_3_OD δ = 49.0 ppm). High resolution mass spectra (HRMS) were recorded using an Orbitrap-Velos Pro at a resolution of 60,000. All compounds are >95% pure by HPLC analysis.

### Experimental Procedures and Analytical Data

#### Ethyl 2-Amino-4-(4-fluorophenyl)thiophene-3-carboxylate **1a**

To a solution of 4-fluoroacetophenone (2.8 g, 2.4 mL, 20 mmol) in 100 mL dry DCM was added ethyl cyanoacetate (2.7 g, 2.6 mL, 24 mmol). The reaction mixture was cooled to 0 °C and stirred for 0.5 h. Subsequently, TiCl_4_ (7.6 g, 4.4 mL, 40 mmol) was added dropwise and the reaction mixture was stirred for 1 h at 0 °C, followed by addition of pyridine in two portions (5.4 mL, 67 mmol). The reaction mixture was stirred for 18 h at RT. Subsequently, the reaction mixture was poured into 100 mL 3 N HCl and extracted twice with 200 mL of DCM. The combined organic layer was washed with brine, filtered over MgSO_4_, and concentrated under reduced pressure. The resulting oil was dissolved in 19 mL THF, followed by addition of elemental sulfur (0.8 g, 25 mmol) and diethylamine (2.8 g, 3.9 mL, 38 mmol). Reaction mixture was stirred for 18 h at RT. The crude was diluted with diethyl ether, washed twice with water and brine, filtered over MgSO_4_, and concentrated under reduced pressure to provide the desired product as a yellow solid (4.9 g, 18.5 mmol, 92%). ^1^H NMR (500 MHz, DMSO-*d*_6_): δ 7.42 (s, 2H), 7.26 (ddd, *J* = 8.9, 5.6, 2.7 Hz, 2H), 7.13–7.07 (m, 2H), 6.16 (s, 1H), 3.95 (q, *J* = 7.1 Hz, 2H), 0.91 (t, *J* = 7.1 Hz, 3H); ^13^C NMR (126 MHz, DMSO-*d*_6_): δ 161.7 (d, *J* = 242.6 Hz), 135.1 (d, *J* = 3.2 Hz), 130.9 (d, *J* = 8.2 Hz), 114.4 (d, *J* = 21.2 Hz), 105.7 (d, *J* = 7.3 Hz), 59.1, 14.2; MS (ESI) *m*/*z*: calcd for C_13_H_13_FNO_2_S [M + H]^+^, 266.0646; found [M + H]^+^, 266.0645.

#### Ethyl 2-Amino-4-(4-bromophenyl)thiophene-3-carboxylate **1b**

To a solution of the 4-bromoacetophenone (4.0 g, 20 mmol) in 100 mL dry DCM was added ethyl cyanoacetate (2.7 g, 2.6 mL, 24 mmol). The reaction mixture was cooled to 0 °C and stirred for 0.5 h. Subsequently, TiCl_4_ (7.6 g, 4.4 mL, 40 mmol) was added dropwise and the reaction mixture was stirred for 1 h at 0 °C, followed by addition of pyridine in two portions (5.4 mL, 67 mmol). Reaction mixture was stirred for 18 h at RT. The crude was poured into 100 mL 3 N HCl and extracted twice with 200 mL of DCM. Subsequently, the combined organic layer washed with brine, filtered over MgSO_4_, and concentrated under reduced pressure. The resulting oil was dissolved in 19 mL THF, followed by addition of elemental sulfur (0.8 g, 25 mmol) and diethylamine (2.8 g, 3.9 mL, 38 mmol). Reaction mixture was stirred for 18 h at RT. The crude was diluted with diethyl ether, washed twice with water and brine, filtered over MgSO_4_, and concentrated under reduced pressure. The crude mass was purified by silica gel flash chromatography using 10% EtOAc/PE as an eluent to provide the desired product as a yellow solid (5.6 g, 17 mmol, 86%). ^1^H NMR (500 MHz, CDCl_3_): δ 7.47–7.39 (m, 2H), 7.19–7.13 (m, 2H), 6.14 (s, 2H), 6.03 (s, 1H), 4.06 (q, *J* = 7.1 Hz, 2H), 0.98 (t, *J* = 7.1 Hz, 3H); ^13^C NMR (126 MHz, CDCl_3_): δ 165.6, 164.2, 140.3, 137.5, 130.7, 130.4, 120.9, 105.9, 105.7, 59.7, 13.9; HRMS (ESI) *m*/*z*: calcd for C_13_H_13_BrNO_2_S [M + H]^+^, 325.9845; found [M + H]^+^, 325.9842.

#### 4-(4-Fluorophenyl)-2-hydroxythiophene-3-carbonitrile **2a**

To a refluxing stirred solution of ethyl 2-amino-4-(4-fluorophenyl) thiophene-3-carboxylate (2.61 g, 9.83 mmol) in 20 mL of EtOH was added dropwise a solution consisting of sodium (452 mg, 19.7 mmol) in 20 mL EtOH. Reaction mixture was stirred for 4 h while refluxing. Subsequently, reaction mixture was allowed to cool to RT, and 80 mL water was added and stirred for 1 h at RT. The crude was filtered and the filtrate was acidified with 37% HCl_aq_ until a pH of 2 was reached. The resulting precipitate was filtered and air dried to provide the desired product as an off-white solid (647 mg, 2.95 mmol, 30%).^1^H NMR (500 MHz, DMSO-*d*_6_): δ 7.62–7.57 (m, 2H), 7.34–7.26 (m, 2H), 6.78 (s, 1H).

#### 4-(4-Bromophenyl)-2-hydroxythiophene-3-carbonitrile **2b**

To a refluxing stirred solution of ethyl 2-amino-4-(4-bromophenyl)thiophene-3-carboxylate (682 mg, 2.1 mmol) in 2.5 mL EtOH was added dropwise sodium ethoxide (285 mg, 4.2 mmol) in 2.5 mL EtOH. Reaction mixture was stirred for 4 h while refluxing. Subsequently, reaction mixture was allowed to cool to RT, and 20 mL water was added and stirred for 1.5 h at RT. The crude was filtered and the filtrate was acidified with 37% HCl_aq_ until a pH of 2 was reached. The resulting precipitate was filtered, air dried, and purified by silica gel flash chromatography using 0–4% MeOH/DCM as eluent to provide the desired product as an off-white solid (303 mg, 1.1 mmol, 52%).^1^H NMR (500 MHz, DMSO-*d*_6_): δ 7.64 (d, *J* = 8.0 Hz, 2H), 7.50 (d, *J* = 8.0 Hz, 2H), 6.82 (s, 1H); ^13^C NMR (126 MHz, DMSO-*d*_6_): δ ^13^C NMR (126 MHz, DMSO): δ 175.1, 136.0, 133.5, 132.0, 131.8, 129.1, 121.5, 115.4, 108.1, 87.7.

#### *tert*-Butyl (4-(((3-Cyano-4-(4-fluorophenyl)thiophen-2-yl)oxy)methyl)benzyl)carbamate **3a**

A solution consisting of 4-(4-fluorophenyl)-2-hydroxythiophene-3-carbonitrile (647 mg, 2.95 mmol), *tert*-butyl (4-(bromomethyl)benzyl)carbamate (976 mg, 3.25 mmol), and potassium carbonate (450 mg, 3.25 mmol) in 10 mL ACN was stirred for 18 h at 80 °C. The reaction mixture was concentrated under reduced pressure. The crude mass was purified by silica gel flash chromatography with 0–100% EtOAc/PE to provide the desired product as a colorless oil (580 mg, 1.3 mmol, 45%). ^1^H NMR (500 MHz, CDCl_3_): δ 7.59–7.50 (m, 2H), 7.43 (d, *J* = 7.8 Hz, 2H), 7.33 (d, *J* = 7.8 Hz, 2H), 7.14–7.07 (m, 2H), 6.53 (s, 1H), 5.26 (s, 2H), 4.90 (s, 1H), 4.34 (d, *J* = 6.1 Hz, 2H), 1.46 (s, 10H); ^13^C NMR (126 MHz, CDCl_3_): δ 163.9, 138.7, 133.0, 130.0, 129.3, 129.2, 128.5, 127.9, 116.0, 115.8, 114.2, 107.7, 44.3, 28.4 ppm.

#### *tert*-Butyl (4-(((4-(4-Bromophenyl)-3-cyanothiophen-2-yl)oxy)methyl)benzyl)carbamate **3b**

A solution consisting of 4-(4-bromophenyl)-2-hydroxythiophene-3-carbonitrile (380 mg, 1.73 mmol), *tert*-butyl (4-(bromomethyl)benzyl)carbamate (572 mg, 1.9 mmol), and potassium carbonate (263 mg, 1.9 mmol) in 10 mL ACN was stirred at 80 °C for 18 h. The reaction mixture was concentrated under reduced pressure. The crude mass was purified by silica gel flash chromatography with 10–20% EtOAc/PE to provide the desired product as a brown solid (654 mg, 0.35 mmol, 76%). ^1^H NMR (500 MHz, CDCl_3_): δ 7.55 (dd, *J* = 8.6 Hz, 2.5 Hz, 2H), 7.43 (dd, *J* = 8.4 Hz, 5.1 Hz, 4H), 7.33 (d, *J* = 10 Hz, 2H), 6.55 (s, 1H), 5.26 (s, 2H), 4.92 (s, 1H), 4.34 (s, 2H), 1.46 (s, 9H); ^13^C NMR (126 MHz, CDCl_3_): δ 140.3, 138.6, 133.1, 132.9, 132.2, 129.1, 128.6, 128,0, 122.9, 114.2, 108.3, 80.2, 77.0, 44.4, 28.5; HRMS (APCI) *m*/*z*: calcd for C_24_H_24_O_3_N_2_BrS [M – H]^+^, 499.0686; found [M + H]^+^, 499.0683.

#### 2-((4-(Aminomethyl)benzyl)oxy)-4-(4-fluorophenyl)thiophene-3-carbonitrile Hydrochloride **4a**

*tert*-Butyl (4-(((3-cyano-4-(4-fluorophenyl)thiophen-2-yl)oxy)methyl)benzyl)carbamate (68 mg, 0.16 mmol) was dissolved in 4 mL HCl (4 M in dioxane) and stirred at RT for 4 h. Reaction mixture was concentrated under reduced pressure to provide the desired product as a yellow solid (52 mg, 0.15 mmol, 99%).^1^H NMR (500 MHz, methanol-*d*_4_): δ 7.61 (tdd, *J* = 8.8, 5.6, 1.9 Hz, 4H), 7.56–7.51 (m, 2H), 6.91–6.86 (m, 1H), 5.41 (d, *J* = 2.6 Hz, 2H), 4.17–4.13 (m, 2H); 13C NMR (CDCl_3_, 126 MHz): ^13^C NMR (126 MHz, methanol-*d*_4_): δ 172.9, 162.3, 160.4, 136.4, 134.3, 132.4, 127.6, 127.5, 127.5, 127.3, 113.8, 113.7, 112.1, 107.2, 74.7, 41.0; HRMS (APCI) *m*/*z*: calcd for C_19_H_16_ON_2_FS [M – H]^+^: 339.0962; found [M – H]^+^: 339.0958.

#### *tert*-Butyl (4-(((3-Cyano-4-(4-(4,4,5,5-tetramethyl-1,3,2-dioxaborolan-2-yl)phenyl)thiophen-2-yl)oxy)methyl)benzyl)carbamate **4b**

A solution of (4-(((4-(4-bromophenyl)-3-cyanothiophen-2-yl)oxy)methyl)benzyl)carbamate (554 mg, 1.1 mmol) in 10 mL anhydrous dioxane was placed under nitrogen and degassed for 10 min. Bispinacolato diboron (338 mg, 1.3 mmol), potassium acetate (327 mg, 3.3 mmol), and [1,1′-bis(diphenylphosphino)ferrocene]palladium(II) dichloride (73 mg, 0.1 mmol) was added. Reaction mixture was stirred at 95 °C for 17 h. The crude was extracted thrice with EtOAc. The combined organic layer was washed with brine, filtered over MgSO_4_, and concentrated under reduced pressure. Silica gel flash chromatography using 10–30% EtOAc/PE as eluent provided the desired product as a yellow solid (186 mg, 0.34 mmol, 42%).^1^H NMR (CDCl_3_, 500 MHz): δ 1H NMR (500 MHz, CDCl3): δ 7.87–7.84 (m, 2H), 7.59–7.56 (m, 2H), 7.44–7.41 (m, 2H), 7.32 (d, *J* = 7.9 Hz, 2H), 6.60 (s, 1H), 5.26 (s, 2H), 4.33 (d, *J* = 6.1 Hz, 2H), 1.46 (s, 9H), 1.35 (s, 12H), 1.23 (s, 8H).; 13C NMR (CDCl_3_, 126 MHz): δ 151.3 (Cq), 149.3 (CH), 123.7 (CH), 37.0 (CH2), 23.2 (CH2),13.5 (CH3) ppm; HRMS (APCI) *m*/*z*: calcd for C_30_H_36_O_5_N_2_BS [M – H]^+^, 546.2469; found [M + H]^+^, 546.2466.

#### (3-(2,3-Dihydrobenzo[*b*][1,4]dioxin-6-yl)-2-methylphenyl)methanol **5**

A mixture of 3-bromo-2-methylphenyl)methanol (5.2 g, 25.9 mmol) and (2,3-dihydrobenzo[*b*][1,4]dioxin-6-yl)boronic acid in toluene/ethanol/sat. aq sodium bicarbonate solution (5:1:5, 0.3 M) was placed under nitrogen and degassed for 10 min. [1,1′-Bis(diphenylphosphino)ferrocene]palladium(II) chloride (95 mg, 0.1 mmol) was added, and the reaction mixture was heated to 85 °C for 12 h. Ethyl acetate and water were added to the reaction mixture. The organic phase was washed with 1 M NaOH solution and brine. The organic layer was filtered over MgSO_4_ and concentrated under reduced pressure. The crude product was purified by silica gel flash chromatography using 0–100% EtOAc/PE as eluent and provided the desired product as a colorless solid (6.0 g, 23.4 mmol, 90%); ^1^H NMR (CDCl_3_, 500 MHz): δ 7.35 (dd, *J* = 7.6, 1.6 Hz, 1H), 7.21 (t, *J* = 7.6 Hz, 1H), 7.16 (dd, *J* = 7.6, 1.6 Hz, 1H), 6.89 (d, *J* = 8.2 Hz, 1H), 6.80 (d, *J* = 2.1 Hz, 1H), 6.75 (dd, *J* = 8.2, 2.1 Hz, 1H), 4.73 (s, 2H), 4.28 (s, 4H), 2.24 (s, 3H); ^13^C NMR (CDCl_3_, 126 MHz): δ 143.1, 142.6, 142.3, 139.3, 135.5, 133.7, 129.6, 126.6, 125.6, 122.6, 118.3, 116.9, 64.5, 64.5, 64.0, 16.0; HRMS (APCI) *m*/*z*: calcd for C_16_H_15_O_2_ [M + H–H_2_O]+: 239.1067, found [M + H–H_2_O]^+^: 239.1066.

#### 6-(3-(Chloromethyl)-2-methylphenyl)-2,3-dihydrobenzo[*b*][1,4]dioxine **6**

3-(2,3-Dihydrobenzo[*b*][1,4]dioxin-6-yl)-2-methylphenyl)methanol (1.1 g, 4.3 mmol) was dissolved in 10 mL DCM and cooled to 0 °C. Thionyl chloride (3.1 mL, 42.5 mmol) was added dropwise and reaction mixture was allowed to warm to RT and stirred for 18 h. After reaction completion, crude was extracted thrice with EtOAc and sodium bicarbonate. Combined organic layer was filtered over MgSO_4_ and concentrated under reduced pressure to provide the desired product as an orange oil (1.2 g, 4.3 mmol, quant. Yield) ^1^H NMR (500 MHz, CDCl_3_): δ 7.30 (t, *J* = 4.6 Hz, 1H), 7.21–7.19 (m, 2H), 6.90 (d, *J* = 8.2 Hz, 1H), 6.81 (d, *J* = 2.1 Hz, 1H), 6.75 (dd, *J* = 8.2, 2.1 Hz, 1H), 4.67 (s, 2H), 4.29 (s, 4H), 2.33 (s, 3H); ^13^C NMR (126 MHz, CDCl_3_): δ 143.2, 142.8, 142.8, 136.2, 135.3, 135.1, 130.8, 129.0, 125.9, 122.6, 118.3, 117.0, 64.5, 45.7, 16.2; HRMS (APCI) *m*/*z*: calcd for C_16_H_16_ClO_2_ [M + H]^+^, 275.0833; found [M + H]^+^, 275.0832.

#### 5-Chloro-4-((3-(2,3-dihydrobenzo[*b*][1,4]dioxin-6-yl)-2-methylbenzyl)oxy)-2-hydroxybenzaldehyde **7**

A mixture of 6-(3-(chloromethyl)-2-methylphenyl)-2,3-dihydrobenzo[*b*][1,4]dioxine (2.7 g, 9.8 mmol), 5-chloro-2,4-dihydroxybenzaldehyde (1.7 g, 9.8 mmol), potassium iodide (1.6 g, 9.8 mmol), and sodium bicarbonate (0.8 g, 9.8 mmol) was dissolved in 40 mL ACN. The reaction mixture was stirred at 65 °C for 2 days. After reaction completion, reaction mixture was concentrated and crude was extracted thrice with DCM and water. Combined organic layer was concentrated under reduced pressure. Crude was suspended in THF, filtered, and washed with water to provide the desired product as a colorless solid (3.0 g, 7.3 mmol, 74%).^1^H NMR (500 MHz, CDCl_3_): δ 11.46 (s, 1H), 9.72 (s, 1H), 7.57 (s, 1H), 7.47 (dd, *J* = 6.6, 2.5 Hz, 1H), 7.32–7.23 (m, 3H), 6.94 (d, *J* = 8.2 Hz, 1H), 6.86 (d, *J* = 2.0 Hz, 1H), 6.81 (dd, *J* = 8.2, 2.0 Hz, 1H), 6.66 (s, 1H), 5.22 (s, 2H), 4.34 (s, 4H), 2.29 (s, 3H), 1.65 (s, 3H); ^13^C NMR (126 MHz, CDCl_3_): δ 193.9, 163.1, 161.1, 143.2, 142.8, 142.6, 135.2, 134.3, 134.2, 133.6, 130.6, 127.4, 125.8, 122.7, 118.4, 117.0, 115.1, 115.0, 101.7, 70.5, 64.6, 64.6, 25.7, 16.4; HRMS (ESI) *m*/*z*: calcd for C_23_H_20_ClO_5_ [M + H]^+^, 411.0994; found [M + H]^+^, 411.0993.

#### 3-((4-Chloro-5-((3-(2,3-dihydrobenzo[*b*][1,4]dioxin-6-yl)-2-methylbenzyl)oxy)-2-formy)lphenoxy)methyl)benzonitrile **8**

A mixture of 5-chloro-4-((3-(2,3-dihydrobenzo[*b*][1,4]dioxin-6-yl)-2-methylbenzyl)oxy)-2-hydroxybenzaldehyde (1.80 g, 4.39), 3-(chloromethyl)benzonitrile (729 mg, 4.81 mmol), and potassium carbonate (665 mg, 4.81 mmol) was dissolved in 30 mL ACN and 1 mL DMF. Reaction mixture was stirred at 80 °C for 16 h. After reaction completion, crude was extracted thrice with DCM and water. Combined organic layer was filtered over MgSO_4_ and concentrated under reduced pressure. The crude product was purified by silica gel flash chromatography using 0–100% EtOAC/PE) to provide the desired product as a pink solid (4.31 g, 4.32 mmol, 98%).^1^H NMR (500 MHz, CDCl_3_): δ 10.34 (s, 1H), 7.93 (s, 1H), 7.75 (d, *J* = 1.9 Hz, 1H), 7.70 (ddt, *J* = 7.8, 4.9, 1.9 Hz, 2H), 7.57 (t, *J* = 7.8 Hz, 1H), 7.42 (dd, *J* = 5.6, 3.5 Hz, 1H), 7.28 (dd, *J* = 5.8, 2.3 Hz, 2H), 6.94 (d, *J* = 8.2 Hz, 1H), 6.84 (d, *J* = 2.1 Hz, 1H), 6.79 (dd, *J* = 8.2, 2.1 Hz, 1H), 6.64 (s, 1H), 5.23 (s, 2H), 5.21 (s, 2H), 4.34 (s, 4H), 2.31 (s, 3H). ^13^C NMR (126 MHz, CDCl_3_): δ 186.8, 160.7, 160.1, 143.3, 142.9, 142.8, 137.3, 135.0, 134.4, 133.7, 132.3, 131.5, 130.8, 130.7, 130.4, 129.9, 127.5, 125.8, 122.6, 119.5, 118.4, 118.3, 117.2, 117.1, 113.3, 98.8, 70.6, 69.9, 64.6, 64.6, 16.5.; HRMS (APCI) *m*/*z*: calcd for C_31_H_25_ClNO_5_ [M + H]^+^, 526.1416; found [M + H]^+^, 526.1417.

#### (2*R*,4*S*)-1-(5-Chloro-2-((3-cyanobenzyl)oxy)-4-((3-(2,3-dihydrobenzo[*b*][1,4]dioxin-6-yl)-2-methylbenzyl)oxy)benzyl)-4-fluoropyrrolidine-2-carboxylic Acid **9a**

To a stirred solution of 3-((4-chloro-5-((3-(2,3-dihydrobenzo[*b*][1,4]dioxin-6-yl)-2-methylbenzyl)oxy)-2-formylphenoxy)methyl)benzonitrile (150 mg, 0.3 mmol) and (2*S*,4*S*)-4-fluoropyrrolidine-2-carboxylic acid (193 mg, 1.5 mmol) in 3 mL DMF, 3 drops of acetic acid were added and stirring continued for 30 min at RT. Subsequently, sodium cyanoborohydride (105 mg, 1.7 mmol) was added and reaction mixture was heated to 80 °C for 3 h. The crude product was purified by silica gel flash chromatography using 0–12% MeOH/DCM to provide the desired product as a colorless oil (112 mg, 0.2 mmol, 60%).^1^H NMR (500 MHz, CDCl_3_): δ 7.68 (s, 1H), 7.62 (d, *J* = 7.4 Hz, 2H), 7.50 (t, *J* = 7.4 Hz, 1H), 7.40 (s, 1H), 7.35 (dd, *J* = 6.1, 3.0 Hz, 1H), 7.24–7.19 (m, 2H), 6.90 (d, *J* = 8.1 Hz, 1H), 6.80 (d, *J* = 1.9 Hz, 1H), 6.75 (dd, *J* = 8.1, 1.9 Hz, 1H), 6.57 (s, 1H), 5.14 (s, 2H), 5.06 (s, 2H), 4.30 (s, 4H), 4.23 (d, *J* = 12.8 Hz, 1H), 4.03 (d, *J* = 12.8 Hz, 1H), 3.93 (dd, *J* = 9.9, 7.7 Hz, 1H), 3.59 (ddd, *J* = 35.8, 13.5, 3.8 Hz, 1H), 3.20 (dd, *J* = 24.3, 13.5 Hz, 1H), 2.63 (ddd, *J* = 20.9, 15.1, 7.7 Hz, 1H), 2.24 (s, 3H),; ^13^C NMR (CDCl_3_, 126 MHz): δ 13C NMR (126 MHz, CDCl_3_): δ 172.3, 156.1, 155.6, 143.2, 142.8, 142.6, 137.6, 135.1, 134.3, 134.2, 133.1, 132.2, 131.7, 130.8, 130.5, 129.9, 127.4, 125.7, 122.7, 118.5, 118.3, 117.7, 115.7, 113.2, 100.1, 94.0, 92.6, 70.6, 69.8, 66.3, 64.6, 64.6, 58.9, 58.7, 54.5, 37.3, 37.2, 16.4.; HRMS (ESI) *m*/*z*: calcd for C_36_H_33_ClFN_2_O_6_ [M + H]^+^, 643.2006; found [M + H]^+^, 643.2004.

#### (2*R*,4*S*)-1-(5-Chloro-2-((3-cyanobenzyl)oxy)-4-((3-(2,3-dihydrobenzo[*b*][1,4]dioxin-6-yl)-2-methylbenzyl)oxy)benzyl)-4-hydroxypyrrolidine-2-carboxylic Acid **9b**

To a stirred solution of 3-((4-chloro-5-((3-(2,3-dihydrobenzo[*b*][1,4]dioxin-6-yl)-2-methylbenzyl)oxy)-2-formylphenoxy)methyl)benzonitrile (350 mg, 0.7 mmol) and methyl (2*S*,4*R*)-4-hydroxypyrrolidine-2-carboxylic acid (292 mg, 2.0 mmol) in 4 mL DCE/DMF (3:1), 2 drops of acetic acid were added and stirring continued for 30 min at RT. Subsequently, reaction mixture was cooled to 0 °C, sodium triacetoxyborohydride (568 mg, 2.7 mmol) was added and reaction mixture was stirred for 12 h at RT. After reaction completion, the crude was extracted with EtOAc and sat. sodium bicarbonate aqueous solution. Organic layer was washed with brine, filtered over MgSO_4_, and concentrated under reduced pressure. Compound was purified by silica gel flash chromatography using 30–100% EtOAC/PE to provide the desired product as a colorless oil (246 mg, 0.4 mmol, 58%). ^1^H NMR (CDCl_3_, 500 MHz): δ 7.75 (s, 1H), 7.65 (ddt, *J* = 17.5, 7.8, 1.4 Hz, 2H), 7.51 (t, *J* = 7.8 Hz, 1H), 7.40 (dd, *J* = 5.6, 3.5 Hz, 1H), 7.34 (s, 1H), 7.22 (d, *J* = 2.0 Hz, 1H), 7.22 (s, 1H), 6.91 (d, *J* = 8.2 Hz, 1H), 6.81 (d, *J* = 2.0 Hz, 1H), 6.76 (dd, *J* = 8.2, 2.0 Hz, 1H), 6.56 (s, 1H), 5.10 (s, 2H), 5.06 (s, 2H), 4.30 (s, 4H), 4.27–4.22 (m, 1H), 3.82 (d, *J* = 13.3 Hz, 1H), 3.78 (d, *J* = 13.3 Hz, 1H), 3.34 (dd, *J* = 10.1, 4.0 Hz, 1H), 3.04 (dt, *J* = 9.8, 1.5 Hz, 1H), 2.66 (dd, *J* = 9.8, 4.0 Hz, 1H), 2.38 (ddd, *J* = 14.1, 10.1, 5.7 Hz, 1H), 2.28 (s, 3H), 1.95–1.88 (m, 1H). ^13^C NMR (H_2_O + D_2_O, 126 MHz): δ 158.1, 157.9, 144.6, 144.2, 143.9, 139.3, 136.4, 135.9, 135.5, 134.1, 133.9, 133.3, 132.7, 131.3, 131.2, 128.6, 126.6, 123.5, 119.6, 119.3, 118.0, 116.2, 113.9, 113.2, 101.1, 79.5, 79.2, 78.9, 71.5, 71.1, 70.0, 68.7, 65.7, 62.5, 54.7, 39.0, 30.8, 16.7; HRMS (ESI) *m*/*z*: calcd for C_36_H_34_ClN_2_O_7_ [M + H]^+^, 641.2049; found [M + H]^+^, 641.2045.

#### Methyl (2*R*,4*R*)-1-(5-Chloro-2-((3-cyanobenzyl)oxy)-4-((3-(2,3-dihydrobenzo[*b*][1,4]dioxin-6-yl)-2-methylbenzyl)oxy)benzyl)-4-hydroxypyrrolidine-2-carboxylate **9c**

To a stirred solution of 3-((4-chloro-5-((3-(2,3-dihydrobenzo[*b*][1,4]dioxin-6-yl)-2-methylbenzyl)oxy)-2-formylphenoxy)methyl)benzonitrile (296 mg, 0.6 mmol) and methyl (2*S*,4*R*)-4-hydroxypyrrolidine-2-carboxylate (390 mg, 2.7 mmol) in 4 mL DCE/DMF (3:1), 2 drops of acetic acid were added and stirring continued for 30 min at RT. Subsequently, reaction mixture was cooled to 0 °C, sodium triacetoxyborohydride (475 mg, 1.7 mmol) was added and reaction mixture was stirred for 12 h at RT. After reaction completion, the crude was extracted with EtOAc and sat. sodium bicarbonate aqueous solution. Organic layer was washed with brine, filtered over MgSO_4_, and concentrated under reduced pressure. Compound was purified by silica gel flash chromatography using 30–100% EtOAC/PE to provide the desired product as a colorless oil (215 mg, 0.3 mmol, 58%). ^1^H NMR (CDCl_3_, 500 MHz): δ 7.75 (d, *J* = 1.8 Hz, 1H), 7.65 (ddt, *J* = 14.3, 7.7, 1.3 Hz, 2H), 7.51 (t, *J* = 7.7 Hz, 1H), 7.40 (dd, *J* = 5.6, 3.4 Hz, 1H), 7.34 (s, 1H), 7.25–7.19 (m, 2H), 6.91 (d, *J* = 8.2 Hz, 1H), 6.81 (d, *J* = 2.1 Hz, 1H), 6.77 (dd, *J* = 8.2, 2.1 Hz, 1H), 6.56 (s, 1H), 5.10 (s, 2H), 5.06 (s, 2H), 4.31 (s, 4H), 4.27–4.23 (m, 1H), 3.86–3.76 (m, 2H), 3.56 (s, 3H), 3.34 (dd, *J* = 10.1, 4.0 Hz, 1H), 3.05 (dt, *J* = 9.8, 1.5 Hz, 1H), 2.66 (dd, *J* = 9.8, 4.0 Hz, 1H), 2.38 (ddd, *J* = 14.2, 10.1, 5.8 Hz, 1H), 2.28 (s, 3H), 2.06 (d, *J* = 16.3 Hz, 1H), 1.92 (ddt, *J* = 14.2, 3.1, 1.5 Hz, 1H); ^13^C NMR (CDCl_3_, 126 MHz): δ 175.5, 155.6, 154.1, 143.1, 142.7, 142.4, 138.3, 135.1, 134.6, 134.2, 132.2, 131.7, 131.4, 130.6, 130.3, 129.5, 127.43, 125.6, 122.6, 120.4, 118.6, 118.2, 116.9, 115.5, 112.9, 100.5, 71.0, 70.6, 69.7, 64.6, 64.5, 63.2, 62.0, 52.0, 51.0, 39.3, 16.3; HRMS (ESI) *m*/*z*: calcd for C_37_H_36_ClN_2_O_7_ [M + H]^+^: 655.2206; found [M + H]^+^: 655.2204.

#### Methyl (2*R*,4*R*)-1-(5-Chloro-2-((3-cyanobenzyl)oxy)-4-((3-(2,3-dihydrobenzo[*b*][1,4]dioxin-6-yl)-2-methylbenzyl)oxy)benzyl)-4-(tosyloxy)pyrrolidine-2-carboxylate **10**

Methyl (2*R*,4*R*)-1-(5-chloro-2-((3-cyanobenzyl)oxy)-4-((3-(2,3-dihydrobenzo[*b*][1,4]dioxin-6-yl)-2-methylbenzyl)oxy)benzyl)-4-hydroxypyrrolidine-2-carboxylate (215 mg, 0.3 mmol) was dissolved in 6 mL pyridine and cooled to 0 °C. 4-Toluenesulfonyl chloride (76 mg, 0.4 mmol) was added and after 1 h reaction time allowed to warm up to RT. The reaction mixture was stirred 2 days at RT. After reaction completion, crude was extracted twice with ethyl acetate and water. The combined organic layer was washed with brine, filtered over MgSO_4_, and concentrated under reduced pressure. Crude was purified by silica gel flash chromatography using 0–100% EtOAC/PE to provide the desired product as a colorless solid (148 mg, 0.2 mmol, 56%).^1^H NMR (CDCl_3_, 500 MHz): δ (d, *J* = 5.9 Hz, 2H), ppm; ^13^C NMR (CDCl_3_, 126 MHz): δ 7.75 (d, *J* = 8.4 Hz, 2H), 7.72 (d, *J* = 1.6 Hz, 1H), 7.66 (dt, *J* = 7.8, 1.4 Hz, 1H), 7.62 (dt, *J* = 7.8, 1.4 Hz, 1H), 7.51 (t, *J* = 7.7 Hz, 1H), 7.40 (dd, *J* = 5.5, 3.7 Hz, 1H), 7.32–7.28 (m, 3H), 7.24–7.21 (m, 2H), 6.91 (d, *J* = 8.2 Hz, 1H), 6.81 (d, *J* = 2.0 Hz, 1H), 6.77 (dd, *J* = 8.2, 2.0 Hz, 1H), 6.55 (s, 1H), 5.09 (s, 2H), 5.04 (d, *J* = 2.0 Hz, 2H), 4.99–4.95 (m, 1H), 4.31 (s, 4H), 3.85 (d, *J* = 13.6 Hz, 1H), 3.70–3.65 (m, 1H), 3.57 (s, 3H), 3.25 (t, *J* = 7.8 Hz, 1H), 3.15 (d, *J* = 11.4 Hz, 1H), 2.64 (dd, *J* = 11.4, 5.7 Hz, 1H), 2.52–2.44 (m, 1H), 2.44 (s, 3H), 2.28 (s, 3H), 2.24–2.15 (m, 1H); ^13^C NMR (CDCl_3_, 126 MHz): δ 172.7, 155.7, 154.3, 144.9, 143.2, 142.8, 142.5, 138.3, 135.2, 134.7, 134.3, 133.9, 132.2, 131.9, 131.7, 130.7, 130.4, 130.0, 129.9, 129.7, 127.9, 127.5, 125.7, 122.7, 118.7, 118.4, 117.00, 115.6, 112.9, 100.6, 78.7, 70.7, 69.8, 64.6, 64.5, 63.4, 58.4, 52.0, 50.5, 36.8, 21.8, 16.4; HRMS (ESI) *m*/*z*: calcd for C_44_H_42_ClN_2_O_9_S [M + H]^+^, 809.2294; found [M + H]^+^, 809.2294.

### Tracer Production

Radiolabeling of [^18^F]**4a** was based on a recent publication (Mossine et al. 2015) that reports the usage of boronic acids or boronic esters via copper-mediated fluorination.^28^^18^F-fluorine was eluted from an activated QMA cartridge with 2.7 mg tetraethylammonium bicarbonate in 0.4 mL *n*Bu-OH to a reaction vial containing precursor (5–15 mg, 9–28 μmol) and 13.6 mg Cu(OTf)_2_(py)_4_ (13.6 mg, 20 μmol) that were dissolved in 0.8 mL DMA. The reaction proceeded for 15 min at 110 °C with constant stirring. After completion, 100 μL 6 M HCl was added and left stirring for 10 min in the same conditions. The reaction was passed through an activated Sep-Pak C18 light SPE cartridge and purified further with RP HPLC (40% ACN/25 mM PBS; XBridge BEH Shield OBD Prep RP18 5 μm 10 × 250 mm). The formulation was performed by loading the product diluted in 60 mL of water on activated Sep-Pak C18 light SPE cartridge and elution with 0.8 mL EtOH. The organic solvent was diluted with 5 mL of PBS to achieve less than 14% of ethanol in the final solution.

Radiolabeling of [^18^F]**9a** was performed by standardly used nucleophilic fluorination. Briefly, [^18^F]F^–^ from cyclotron was passed through a conditioned QMA light cartridge, dried with air, and eluted with 3.5 mg K_2_CO_3_ and 20 mg K_2.2.2_ in 0.7 mL ACN/0.2 mL water. [^18^F]F^–^ was dried azeotropically (3 × 0.5 mL anhydrous ACN) and precursor was added in anhydrous ACN or DMSO (5 mg, 6.2 μmol, 0.3 mL). The reaction was monitored using TLC plates (eluent and purified further with RP HPLC (50% ACN/0.1 M sodium acetate; XBridge BEH Shield OBD Prep RP18 5 μm 10 × 250 mm). The formulation was performed by loading the product diluted in 60 mL of water on activated Sep-Pak C18 light SPE cartridge and elution with 0.8 mL EtOH. The organic solvent was diluted with 5 mL of water + 6 μL Tween-80 to achieve less than 14% of ethanol in the final solution. For lipophilicity, stability, and ex vivo study, synthesis of [^18^F]BMS1166 was performed in Synthera IBA automation module and purification was performed with a formulation in E&Z automation module.

The identity, purity, and purification of tracers were determined by high-performance liquid chromatography (HPLC). The Waters ACQUITY HPLC system was equipped with a dual wavelength absorbance detector, in-line radioactivity detector, and HPLC column: XBridge Prep Shield and XBridge BEH Shield OBD Prep column (both RP18 5 μm 10 × 250 mm, Waters, USA). Secondary quality control was performed using ultra performance liquid chromatography (UPLC). The Waters ACQUITY HPLC H-class UPLC system was equipped with a dual wavelength absorbance detector in line with LB513 radioactivity detector with an MX50-2 cell (Berthold) and UPLC column: BEH Shield and BEH Phenyl (both RP18 1.7 μm 3.0 × 50 mm, Acquity Waters, USA). For in vitro tracer stability, the radiochemical purity of labeled products was measured using thin-layer chromatography (TLC) (silica TLC plates on aluminum, MerckMilipore, USA). The TLCs were developed with an appropriate MeOH/DCM eluent system. The distribution of the radioactivity among the TLC plate was measured on GE Amersham Typhoon Scanner using phosphorus plates and ImageQuant TL 1D software for data processing (both GE Healthcare Life Science, USA).

The radioactivity was measured using a dose calibrator (VDC-505, Commecer, Netherlands) and 2480 Wizard Detector Gamma Counter (PerkinElmer, Netherlands).

### In Vitro Stability

In vitro stability of [^18^F]**4a** and [^18^F]**9a** was evaluated in Tween-80 or PBS (original diluent) and serum. After purification, samples of tracers were mixed with equal volumes of the formulated tracer and solution in which stability was evaluated (100 μL tracer solution with 100 μL Tween-80/PBS) and measured every 30 min for a 4 h period. The radiochemical purity (RCP) of labeled products was measured using thin-layer chromatography (TLC) (silica TLC plates on aluminum, MerckMilipore, USA). The TLCs were developed with appropriate MeOH/DCM eluent system; 10% MeOH/DCM + NH_3_ for [^18^F]**4a** and 10% MeOH/DCM for [^18^F]**9a**. The distribution of the radioactivity among the TLC plate was measured on GE Amersham Typhoon Scanner using phosphorus plates and ImageQuant TL 1D software for data processing (both GE Healthcare Life Science, USA). The experiment was performed in triplicate.

### Cell Lines

The human tumor cell line H358 (lung adenocarcinoma) was obtained from the American Type Culture Collection and ES2 (human ovarian clear cell carcinoma) cell line was a gift from Dr. Els Berns (Erasmus MC, The Netherlands). Tumor cells were cultured in RPMI 1640 medium (Gibco, Paisley, UK) supplemented with 10% fetal bovine serum (FBS, Bodinco BV, The Netherlands) and maintained in a 5% CO_2_ 37 °C incubator. Cells were tested negative for mycoplasma contamination by PCR analysis and proven their authenticity in short tandem repeat profiling.

CHO-K1 artificial antigen-presenting cells (PD-L1+ aAPC/CHO-K1 cells, called aAPCs) overexpressing TCR ligand and PD-L1, and a modified Jurkat T cell line overexpressing PD-1 with a luciferase reporter under the control of NFAT promoter (PD-1 effector cells, called ECs) were obtained from Promega. PD-L1-expressing aAPC–CHO-K1 cells and PD-1 effector cells were cultured in RPMI 1640 medium (BioWest) supplemented with 10% FBS and 2 mM l-glutamine. Additionally, aAPCs and ECs were propagated in a constant presence of hygromycin B (50 μg/mL) and G418 (250 μg/mL) to provide a stable expression of the introduced genetic constructs. The two latter antibiotics were omitted in the experiments.

### Flow Cytometry

To assess the extracellular expression of PD-L1 on used samples, cells were collected and stained with CD279 (PD-1)-APC and CD274 (PD-L1)-PE antibodies. After incubation at 4 °C for 30 min, cells were washed thrice with PBS + 2% FBS, and their surface expression was measured using BD Accuri c6 flow cytometer (BD Biosciences) and BD FACSVerse flow cytometer (BD Biosciences). Data analysis was performed with the use of Cytobank (www.cytobank.org).

### Western Blotting

To assess the intracellular expression of PD-L1 in used samples, cells were collected and lysed in MPER buffer + 1% protease and 1% phosphatase inhibitor for 30 min on ice followed by pelleting of insoluble material by centrifugation. Sample concentrations were determined using Bradford assay (Bradford, 1976) and suitable amounts were heated to 100 °C in SDS sample buffer + 10% β-mercaptoethanol for 10 min.^39^ Lysates were separated by SDS–PAGE and transferred to the PVDF membrane (Immobilon P, Millipore). Membranes were blocked in 5% milk in TBS + 0.05% Tween-20, probed with the indicated antibodies, and reactive bands visualized using Image Lab software (Bio-Rad).

### Generation of PD-L1 Knock-Out Cell Lines

PD-L1 knock-out cell line was constructed using the CRISPR-Cas9 engineering technique on H358 and ES2. The guide was prepared using primers CACCGTCTTTATATTCATGACCTAC (54 °C) and AAACGTAGGTCATGAATATAAAGAC (51 °C) reported in the literature (Liao et al., 2017).^40^ Guide duplex was ligated with thermocycler, and fused into pSpCas9(BB)-2A-GFP plasmid (pSpCas9(BB)-2A-GFP (PX458) was a gift from Feng Zhang; Addgene plasmid #48138; http://n2t.net/addgene:48138; RRID:Addgene_48138). The plasmid construct was subjected to bacterial transformation on agar Petri dishes containing ampicillin, lysed the next day, and purified using a DNA purification kit (Qiagen). As final validation of obtaining the right plasmid, samples were sent for sequencing to GATC Biotech. Transfection with the specific PD-L1-Cas9-GFP vector was performed using FuGENE HD Transfection Reagent (Promega) according to manufacturer protocol. For the experiment, cells were plated one day before transfection (6-well plate, 0.25–1 × 10^6^ cells in 2 mL of growth medium without antibiotics, 80–90% confluent at the time of transfection). Cells were incubated at 37 °C in a CO_2_ incubator and visualized for positive transfection by green fluorescence positivity under a fluorescent microscope (EVOS FL imaging system, Thermo Fisher). After 4 days, GFP-positive cells were sorted into 96-well plates as single-cell clones using SH800S Sony sorter (Sony, USA). Expanded clones were analyzed by flow cytometry using CD274-PE antibody and selected ones characterized by Western blotting. The clones confirmed with a constitutive knockout of PD-L1 expression were used for the following functional assays.

### Ex Vivo Tumor Cell Analysis

Formalin-fixed paraffin-embedded xenograft tumors (ES2 PD-L1^+/–^ and H358 PD-L1^+/–^) were cut into 4 μm slices and placed on the glass slides. Before each immunohistochemistry and autoradiography experiment, tissue slides were deparaffinized by oven incubation (min 1 h at 60 °C) and xylene washes, then rehydrated through graded alcohol and distilled water. Additionally, heat-induced antigen retrieval with 10 mM citrate buffer pH = 6.0 was performed (15 min 400 W). Snap-frozen human tonsil tissue was a gift from the Pathology Department (UMCG). Before each experiment, they were incubated in cold acetone for 10 min.

For immunohistochemistry, antigen retrieval was followed by endogenous peroxidase block (S2003; Dako), endogenous IgG block (X0909; Dako), and incubation with anti-PD-L1 antibody (5 μg/mL, clone 28–8; Abcam) for 60 min. Next, sections were incubated with anti-rabbit Dako EnVision+ polymer for 30 min (K4010; Dako). The staining was visualized using 3,3′-diaminobenzidine + substrate (K3468; Dako) and counterstained using hematoxylin. The image was recorded on NanoZoomer Digital Slide Scanner and visualized by NDP View software (both Hamamatsu Photonics, Japan).

For autoradiography, antigen retrieval and acetone wash were followed by 30 min of incubation with a freshly formulated tracer at a concentration of 0.2 MBq/mL. After washing twice with cold PBS and once with water, slides were left to dry and exposed to phosphorus plates. The image was recorded on GE Amersham Typhoon Biomolecular Imager and analyzed with ImageQuant TL 1D software (both GE Healthcare Life Science, USA).

### PD-L1 Expression

The PD-L1 protein (18–134) was expressed in the *Escherichia coli* BL21 (DE3) and purified as described previously (Zak et al., 2016).^8^ Protein expression was induced with 1 mM isopropyl β-d-1-thiogalactopyranosid (IPTG) at OD_600_ of 0.8 and the cells were cultured overnight at 37 °C. Afterward protein was refolded by drop-wise dilution into solution containing 0.1 M Tris pH 8.0, 1 M l-arginine hydrochloride, 0.25 mM oxidized glutathione, and 0.25 mM reduced glutathione. After refolding, protein was dialyzed 3 times against buffer containing 10 mM Tris pH 8.0 and 20 mM NaCl. Finally, PD-L1 was purified by SEC (size-exclusion chromatography) on HiLoad 26/600 Superdex 75 column (GE Healthcare) equilibrated with PBS pH 7.4.

### NMR Binding Assay

For NMR measurements, the buffer was exchanged by gel filtration to PBS pH 7.4. 10% (v/v) of D_2_O was added to the samples to provide the lock signal. All spectra were recorded at 300 K using a Bruker Avance III 600 MHz spectrometer equipped with the nitrogen cryo-probe head.

Determination of binding of compounds to PD-L1 was carried out with the ^1^H NMR. The line width broadening in the proton NMR of PD-L1 suggests that the compounds induce protein oligomerization. In all the cases, the well-resolved narrow resonance peaks in the aliphatic region of ^1^H NMR spectrum of apo-PD-L1 exhibited significant broadening upon the addition of each compound indicating a significant increase in the molecular weight of the complex. The molecular weight of each complex was estimated from relaxation time analysis, which can only be explained by the compound-induced PD-L1 dimerization. No significant changes were observed upon the addition of the PD-1 with the tested compounds.

### Microscale Thermophoresis Assay

PD-L1 was expressed as for NMR experiment. It was diluted to 10 μM and labelled with Protein Labeling Kit RED-NHS 2nd Generation from Nanotemper according to the manufacturer’s guidelines. The degree of labelling for PD-L1 was 0.74, which is with agreement with the protocol. Labelled PD-L1 at 100 nM concentration was then mixed with independent series of **4a** and **9a** compound dilution series (*n* = 4) and incubated for 30 min at RT. Next, samples were transferred to dedicated 384-well plate and measured on Diantus equipped with pico detector using auto-excitation and 5 s pulse (Nanotemper) for the affinity measurements. The results were exported, averaged, and fitted in Mathematica 12 using following equations:
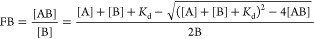
where: FB—fraction bound, [A]—concentration of unlabeled titrated partner, [B]—concentration of fluorescent labeled partner that is fixed, [AB]—concentration of bound complex of A and B, *K*_d_—equilibrium dissociation constant.

### Homogeneous Time-Resolved Fluorescence Assay

The HTRF assay was performed using the certified Cis-Bio assay kit at 20 μL final volume using their standard protocol as described by Musielak et al.^34^ Measurements were performed on individual dilution series to determine the half maximal inhibitory concentration (IC_50_) of tested compounds. After mixing all components according to the Cis-Bio protocol, the plate was incubated for 2 h at RT. TR-FRET measurement was performed on the Tecan Spark 20 M. Collected data was background-subtracted on the negative control, normalized on the positive control, averaged, and fitted with normalized Hill’s equation to determine the IC_50_ value using Mathematica 12. For the compounds with IC_50_ values that were too high due to, e.g., solubility issues, a “dissociation value” at the concentration of 50 μM is presented for the sake of comparability to other inhibitors. The dissociation value represents the percentage of the PD-1/PD-L1 complex that is undissolved.

### Promega PD-1/PD-L1 Blockade Assay

The aAPCs were seeded in white flat bottom 96-well plates at the density of 10 000 cells/well in the culture medium 24 h before the assay and grown overnight in a 5% CO_2_ 37 °C incubator. On the day of the experiment, serial dilutions of tested compounds were prepared first in DMSO and then formulated in RPMI 1640-containing 1% FBS to keep the constant DMSO concentration of 0.1% (v/v). As a negative control, DMSO was used in assay buffer at the concentration of 0.1% (v/v). As a positive control, a serial dilution of durvalumab (an anti-PD-L1 antibody) in assay buffer was used. Meanwhile, the medium of the aAPCs cells was removed, and the compounds’ dilutions were added to the plate (40 μL/well). PD-1 effector Jurkat cells were pelleted and resuspended in the assay buffer. The cell suspension was distributed over the inner 60 wells of the assay plate (20 000 cells/well, 40 μL). The plates were incubated for 6 h in a 5% CO_2_ incubator at 37 °C. Once the assay time was over, the plate was taken out of the incubator to equilibrate to room temperature. Before measurements, a 75 μL per well of Bio-Glo luciferase substrate was added to the inner 60 wells of the plate. 75 μL was also added to selected wells for background measurement. The measurement was done on a Spark microplate reader (Tecan), using the standard settings for luminescence reading.

### Cytotoxicity Assay

5000 ECs (Jurkat T cell line overexpressing PD-1 with a luciferase reporter under the control of NFAT promoter) were seeded on transparent 96-well plates and cultured for 48 h in the presence of increasing concentrations of the compounds or DMSO as a control (the concentration of DMSO was kept constant in all samples). Following the treatment, a metabolic activity test was performed with the use of Biolog Redox Dye Mix MB (BioLog), according to the manufacturer’s instructions.

### Molecular Docking

The structure of **4a**, **9a**, and **9b** compounds was prepared and minimized in UCSF Chimera software with AutoDock Vina.^41,42^ The structure of the compound and PD-L1 dimer (PDB: 6R3K) were prepared in PyMol and UCSF Chimera. All water molecules and original ligand (BMS1166) were removed and polar hydrogens atoms were added to the receptor. A grid box of dimensions 20 × 20 × 20 Å and following coordinates *x*: −8.746, *y* = 18.049, and *z* = −21.934 was placed at the interface of PD-L1 homodimer. Docking was carried out with exhaustiveness = 8.^11^ The obtained binding poses were carefully visually inspected in PyMol and UCSF Chimera.

### Statistics

All values were expressed as mean ± SD unless stated otherwise. Each value is the mean of at least three independent experiments in each group. Statistical analyses were performed in GraphPad Prism version 7.0 (GraphPad Software) using the Mann–Whitney test (2 groups, nonparametric) or one-way analysis of variance (ANOVA) with Tukey Post-Hoc test (>2 groups, nonparametric). The asterisk (*) indicates the values that are significantly different from control (*p < 0.05, **p < 0.051 and ***p < 0.001).

## Supporting Information Available

^1^H, ^13^C NMR spectra, HRMS, and UPLC chromatograms for the synthesized compounds; ^1^H–^15^N HMQC NMR spectra of PD-L1 and PD-L1 with inhibitor, Jurkat cell line viability in PD-1/PD-L1 ICB assay, and MST affinity analysis for **4a**, **9a**, **9b**; labeling optimization, HPLC chromatograms, and stability in solution and serum for [^18^F]**4a** and [^18^F]**9a**; and flow cytometry and Western blot for wild-type and PD-L1 knock-out of H358 and ES2 cancer cell lines, ex vivo autoradiography of cancer cell lines, and tonsils incubated with [^18^F]**4a** and [^18^F]**9a** (PDF)
Molecular formula strings for the synthesized compounds (CSV)

## Abbreviations

aAPCs: antigen-presenting cells
CDR: complementarity-determining region
CTLA-4: cytotoxic T cell antigen 4
G-3CR: Gewald three component reaction
HTRF: homogeneous time resolved fluorescence
IL-2: interleukin-2
MST: microscale thermophoresis
NFAT: nuclear factor of activated T-cells
PD-1: programmed cell death protein 1
PD-L1: programmed death-ligand 1
SMI: small molecular weight inhibitor
SPR: surface plasmon resonance.
