# Supplementary material for: Design, Synthesis, and Biological Evaluation of 2-Hydroxy-4-phenylthiophene-3-carbonitrile as PD-L1 Antagonist and Its Comparison to Available Small Molecular PD-L1 Inhibitors
Source: J Med Chem. 2023 Jul 14;66(14):9577–91. doi: 10.1021/acs.jmedchem.3c00254 (PMC10388299; doi:10.1021/acs.jmedchem.3c00254)
Supplement: Supplementary file 1 — jm3c00254_si_001.pdf [file jm3c00254_si_001.pdf]

Supporting Information

Design, synthesis, and biological evaluation of 2-hydroxy-4-phenylthiophene-3-carbonitrile as PD-L1 antagonist and its comparison to available small molecular PD-L1 inhibitors

Marta A. Ważyńska[†1,] Roberto Butera[†2], Marta Requesens,[1] Annechien Plat,[1] Tryfon Zarganes-Tzitzikas[3], Constantinos G. Neochoritis[4], Jacek Plewka[5], Lukasz Skalniak[5], Justyna Kocik-Krol[5,6], Bogdan Musielak[5], Katarzyna Magiera-Mularz[5], Ismael Rodriguez[5,6], Simon N. Blok[7], Marco de Bruyn[1], Hans W. Nijman[1], Philip H. Elsinga[7], Tad A. Holak[5], and Alexander Dömling*[2,8]

[1]University of Groningen, University Medical Center Groningen, Department of Obstetrics and Gynecology, Hanzeplein 1, 9713 GZ Groningen, The Netherlands

[2]University of Groningen, Department of Drug Design, A. Deusinglaan 1, 9713 AV Groningen, The Netherlands

[3]Alzheimer's Research UK Oxford Drug Discovery Institute, Centre for Medicines Discovery, Nuffield Department of Medicine, NDM Research Building, Roosevelt Drive, Oxford, OX3 7FZ, United Kingdom

[4]Department of Chemistry, University of Crete, Voutes, 70013, Heraklion, Greece

[5]Department of Organic Chemistry, Faculty of Chemistry, Jagiellonian University, Gronostajowa 2, 30-387 Krakow, Poland

[6]Jagiellonian University, Doctoral School of Exact and Natural Sciences, Prof. St. Łojasiewicz St 11, 30-348, Krakow, Poland

[7]University of Groningen, University Medical Center Groningen, Department of Nuclear Medicine and MolecularImaging, Hanzeplein 1, 9713 GZ Groningen, The Netherlands

[8]Institute of Molecular and Translational Medicine, Faculty of Medicine and Dentistry and Czech Advanced Technology and Research Institute, Palacky University in Olomouc, Olomouc, Czech Republic. alexander.domling@upol.cz

† authors contributed equally
* corresponding author

Table of content

NMR spectra 3

HRMS 19

UPLC 23

Supplementary Figures and Tables 24

NMR spectra

Ethyl 2-amino-4-(4-fluorophenyl)thiophene-3-carboxylate ***1a***

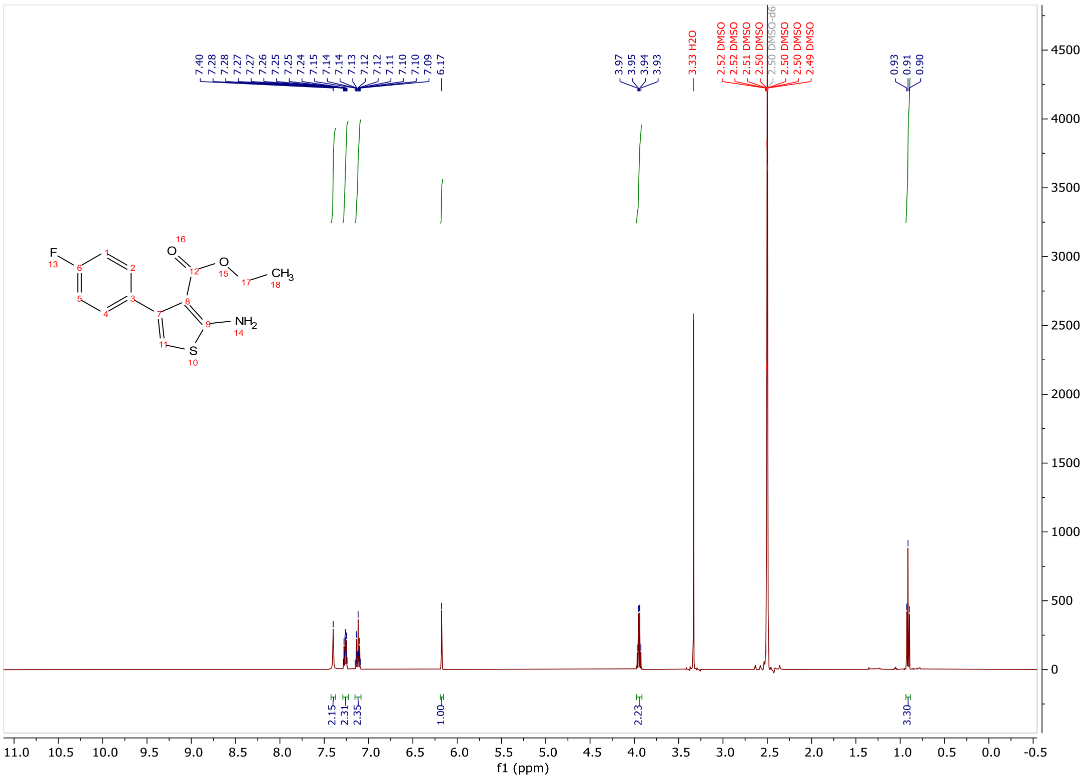

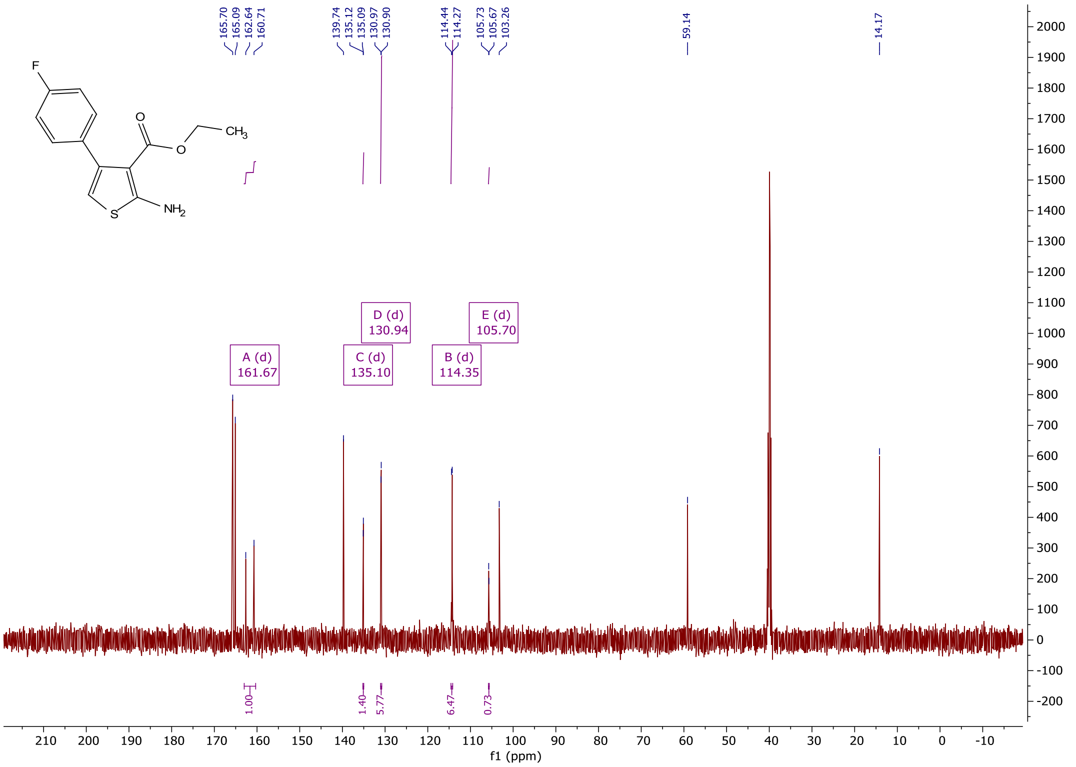

Ethyl 2-amino-4-(4-bromophenyl)thiophene-3-carboxylate ***1b***

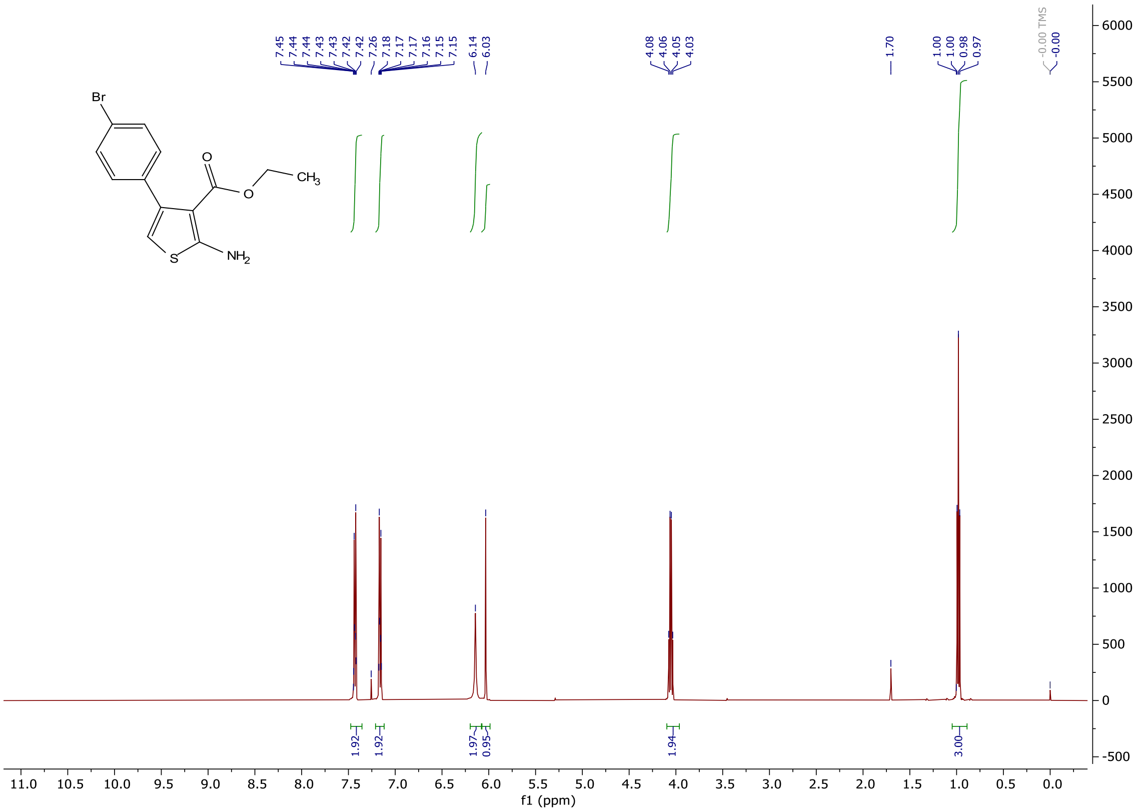

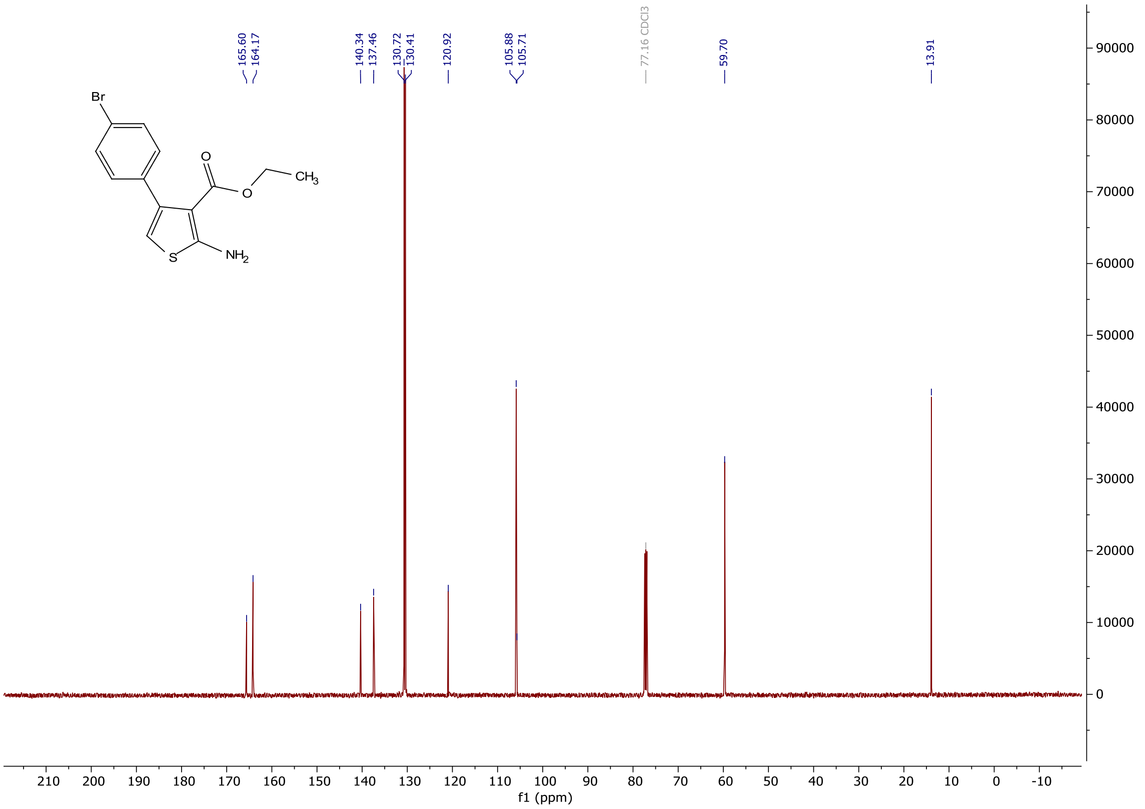

*4-(4-fluorophenyl)-2-hydroxythiophene-3-carbonitrile **2a***

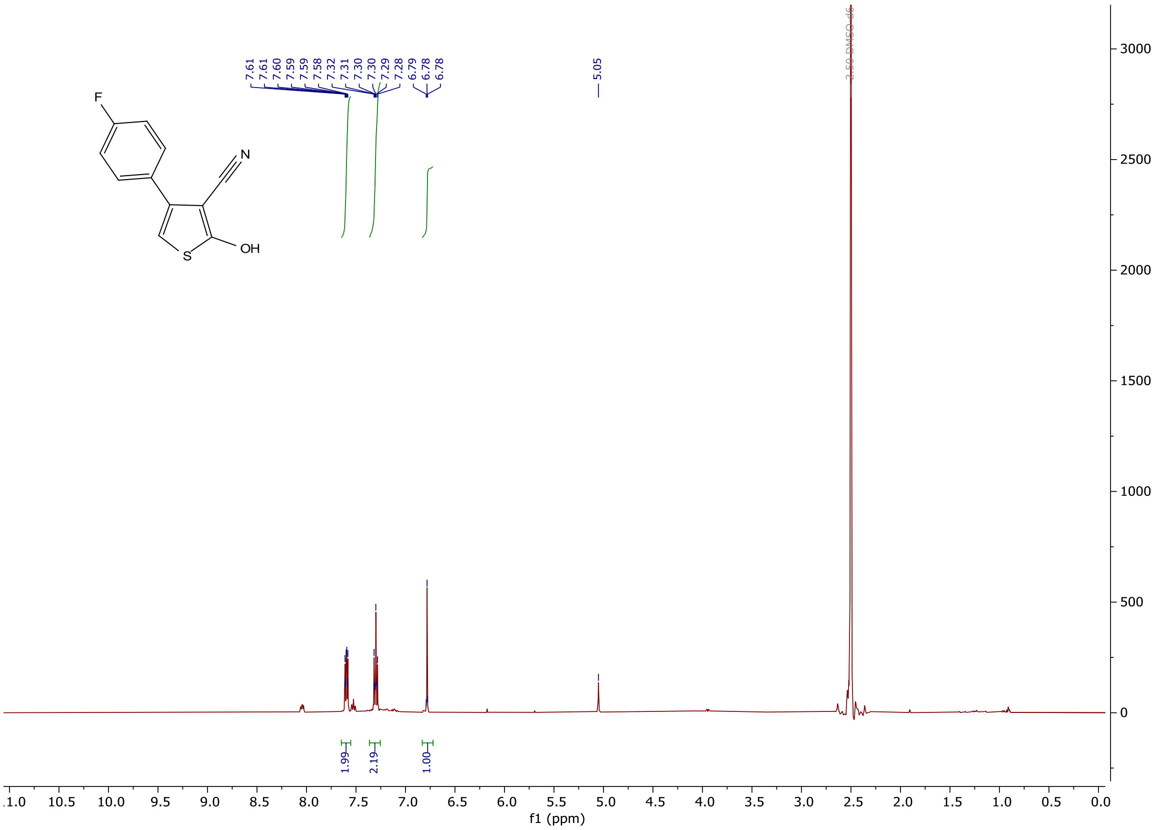

4-(4-bromophenyl)-2-hydroxythiophene-3-carbonitrile ***2b***

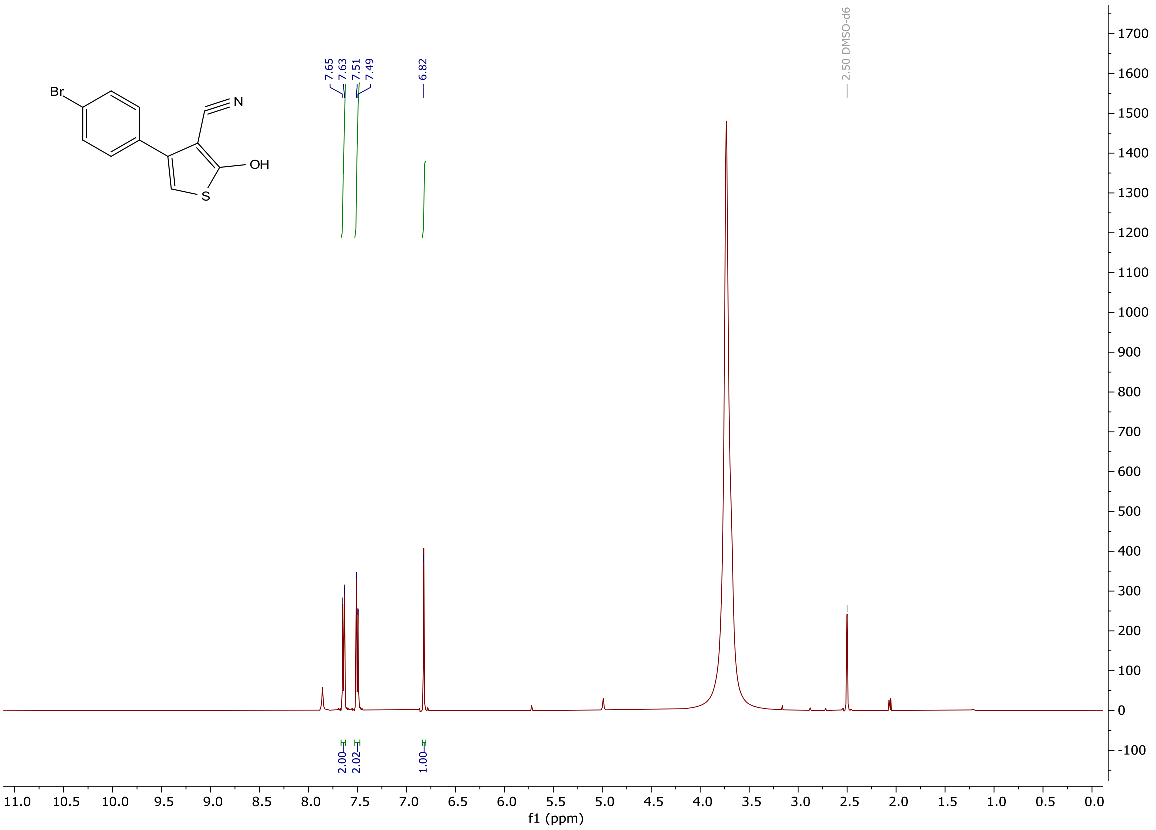

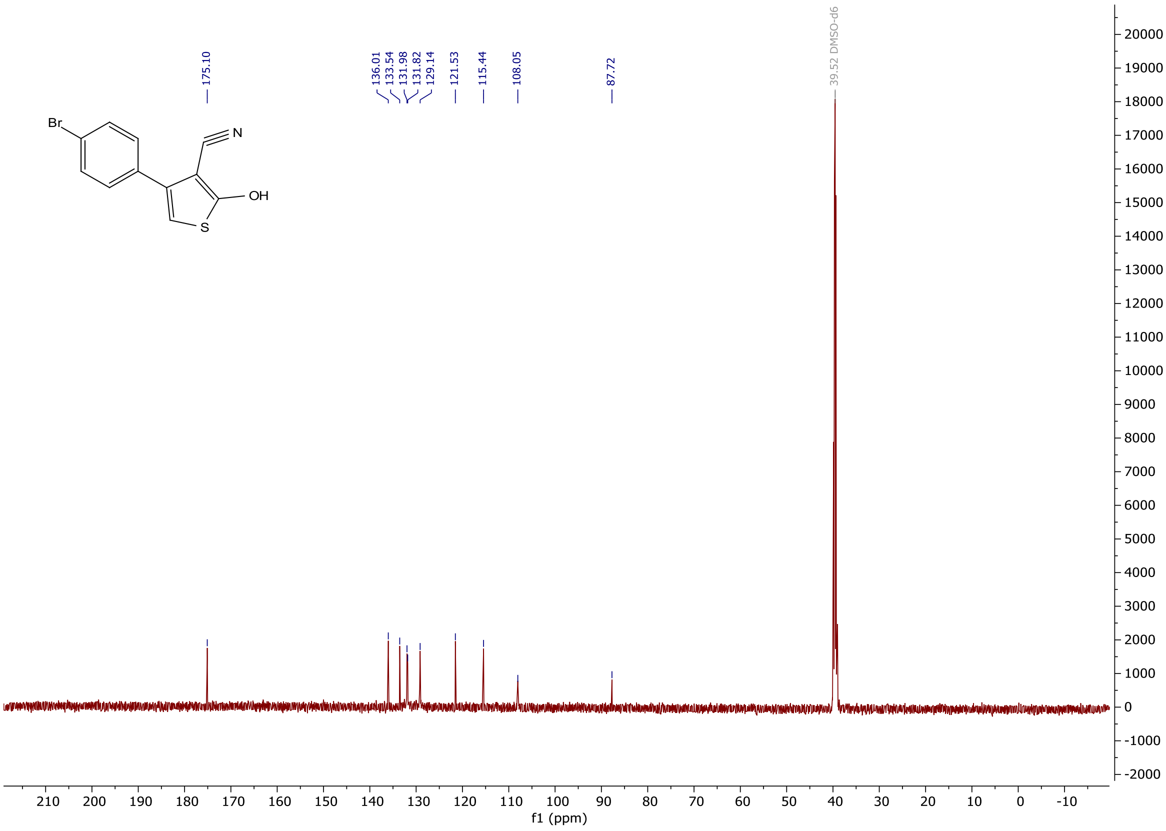

Tert-butyl (4-(((3-cyano-4-(4-fluorophenyl)thiophen-2-yl)oxy)methyl)benzyl)carbamate ***3a***

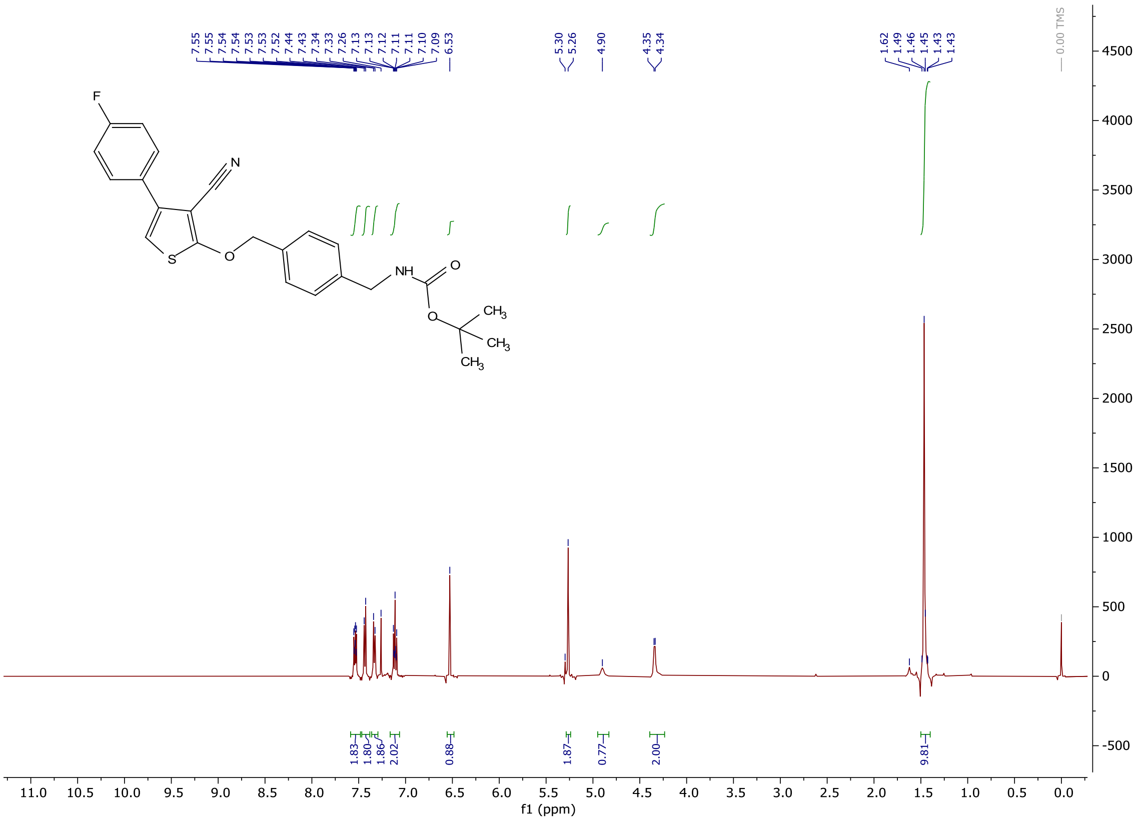

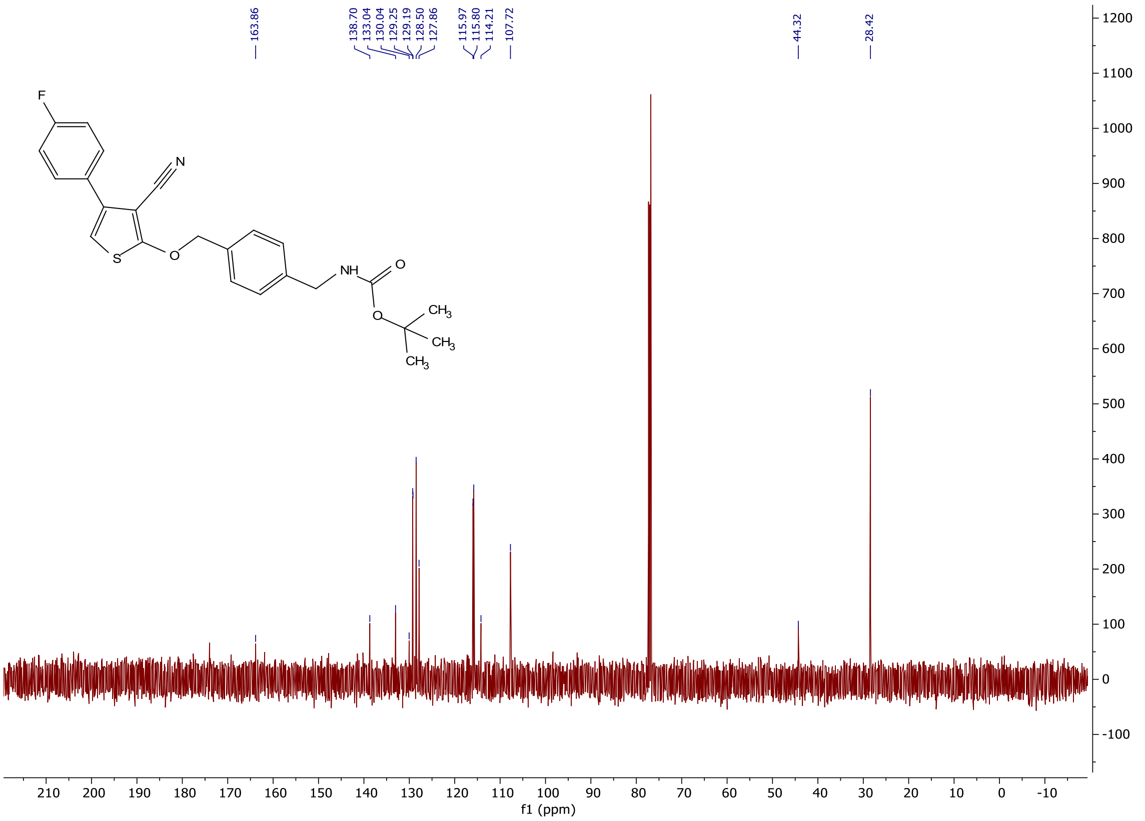

Tert-butyl (4-(((4-(4-bromophenyl)-3-cyanothiophen-2-yl)oxy)methyl)benzyl)carbamate ***3b***

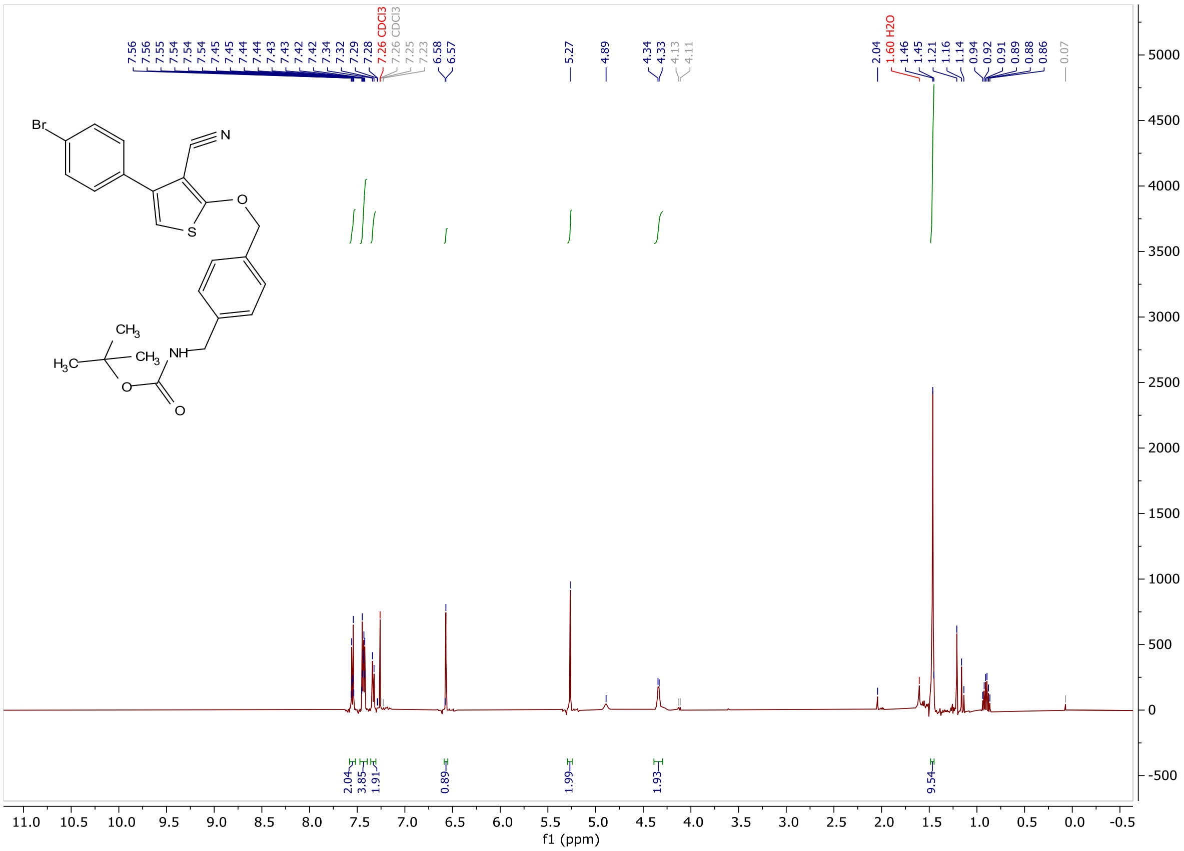

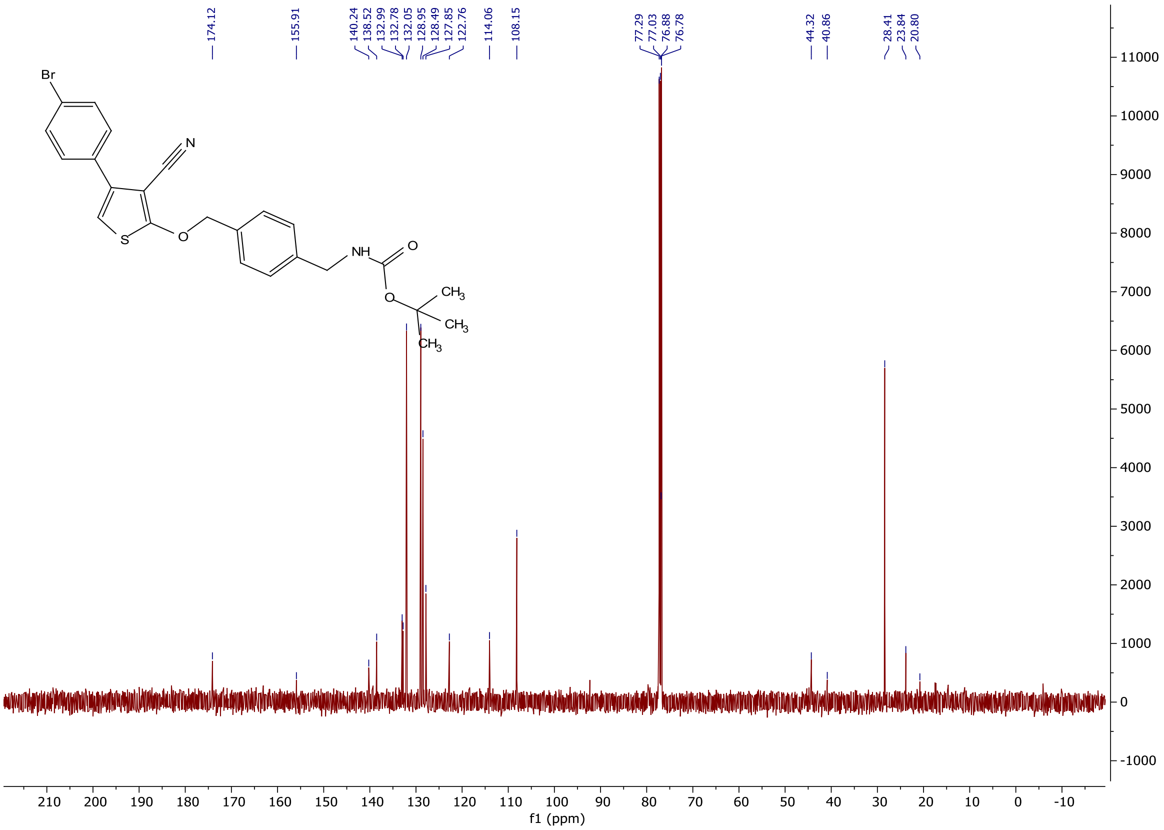

2-((4-(aminomethyl)benzyl)oxy)-4-(4-fluorophenyl)thiophene-3-carbonitrile hydrochloride ***4a***

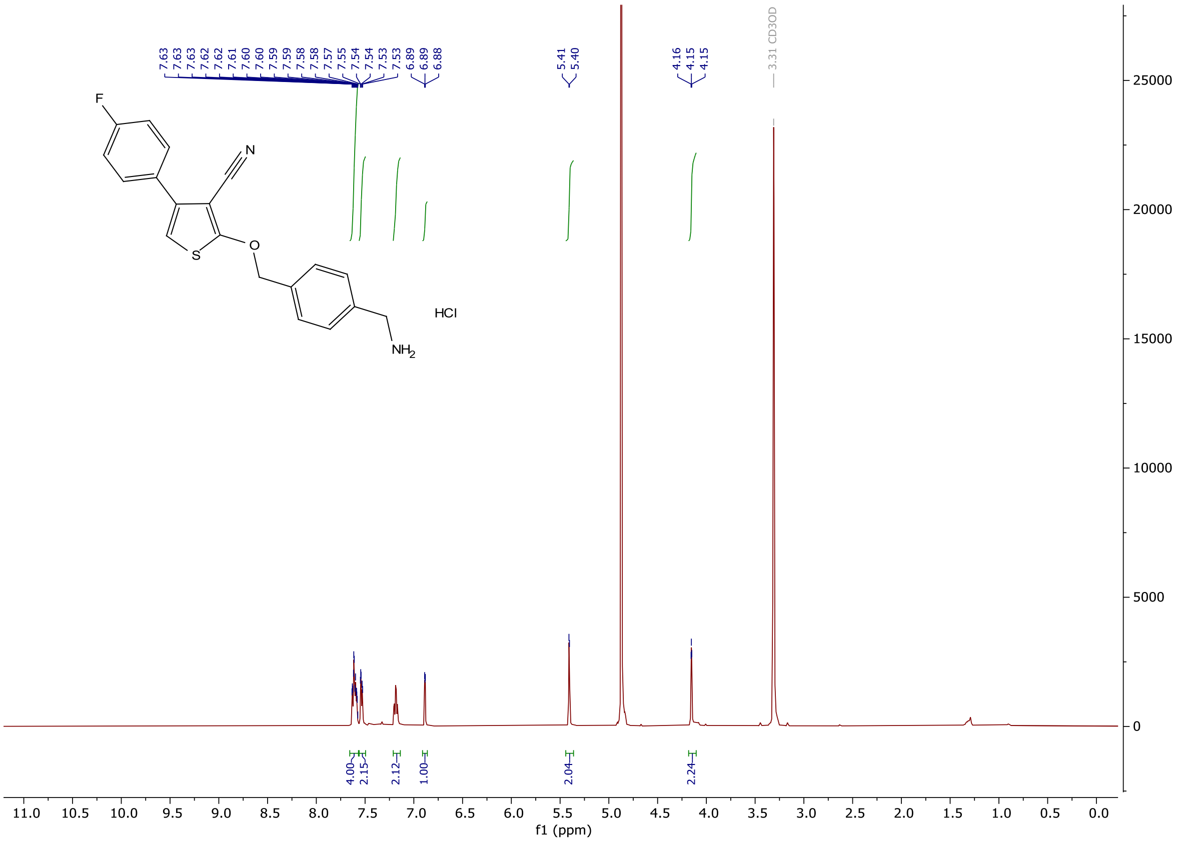

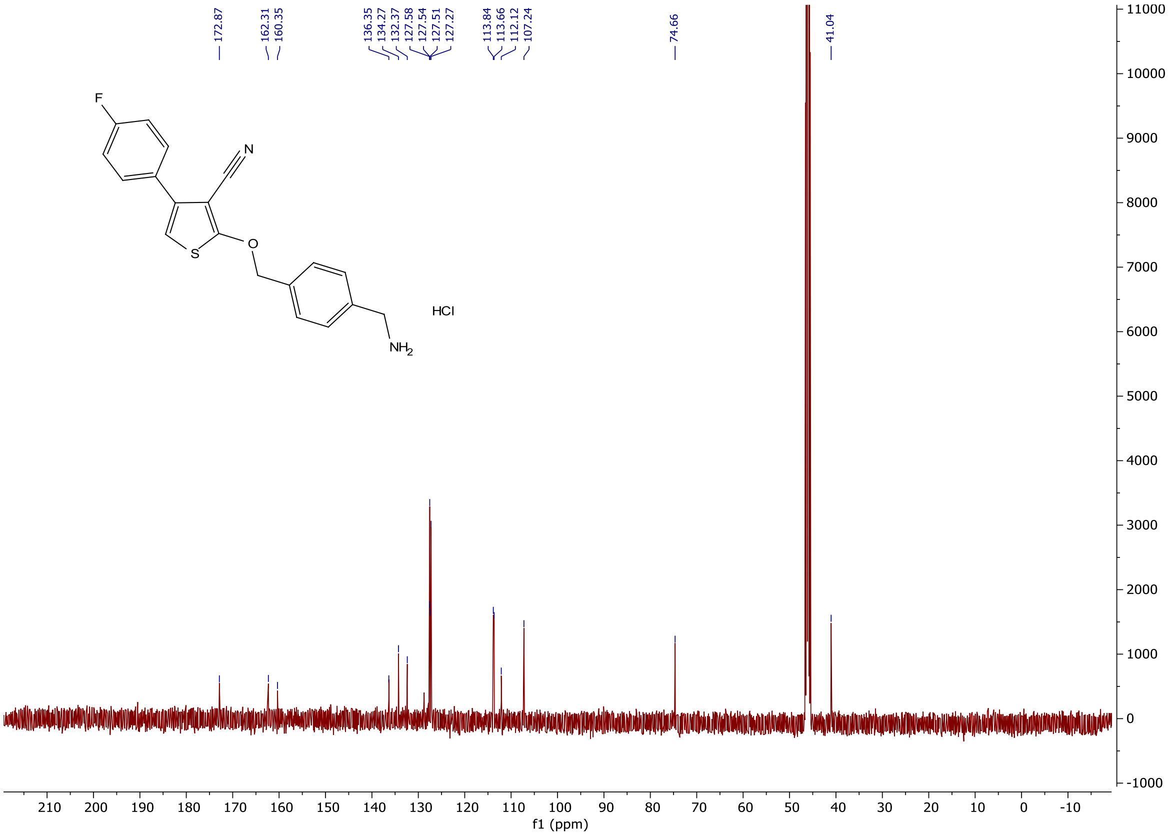

Tert-butyl (4-(((3-cyano-4-(4-(4,4,5,5-tetramethyl-1,3,2-dioxaborolan-2-yl)phenyl)thiophen-2-yl)oxy)methyl)benzyl)carbamate ***4b***

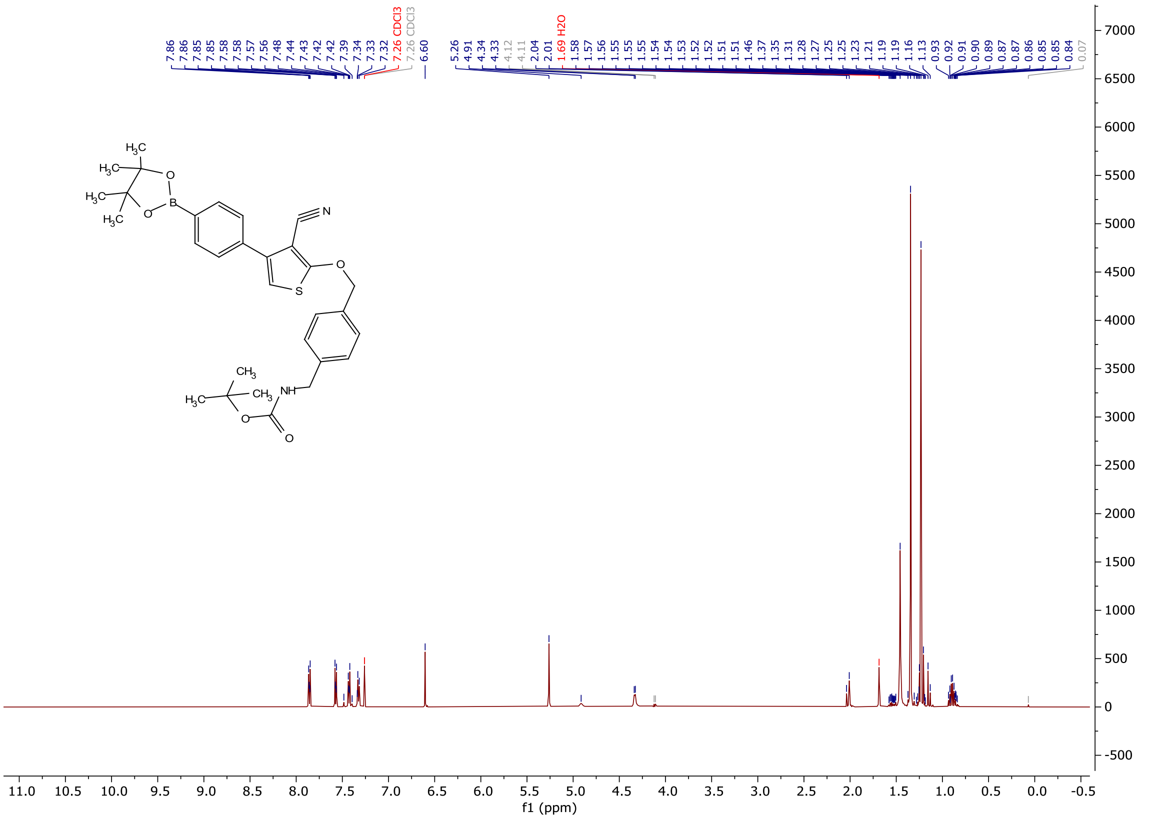

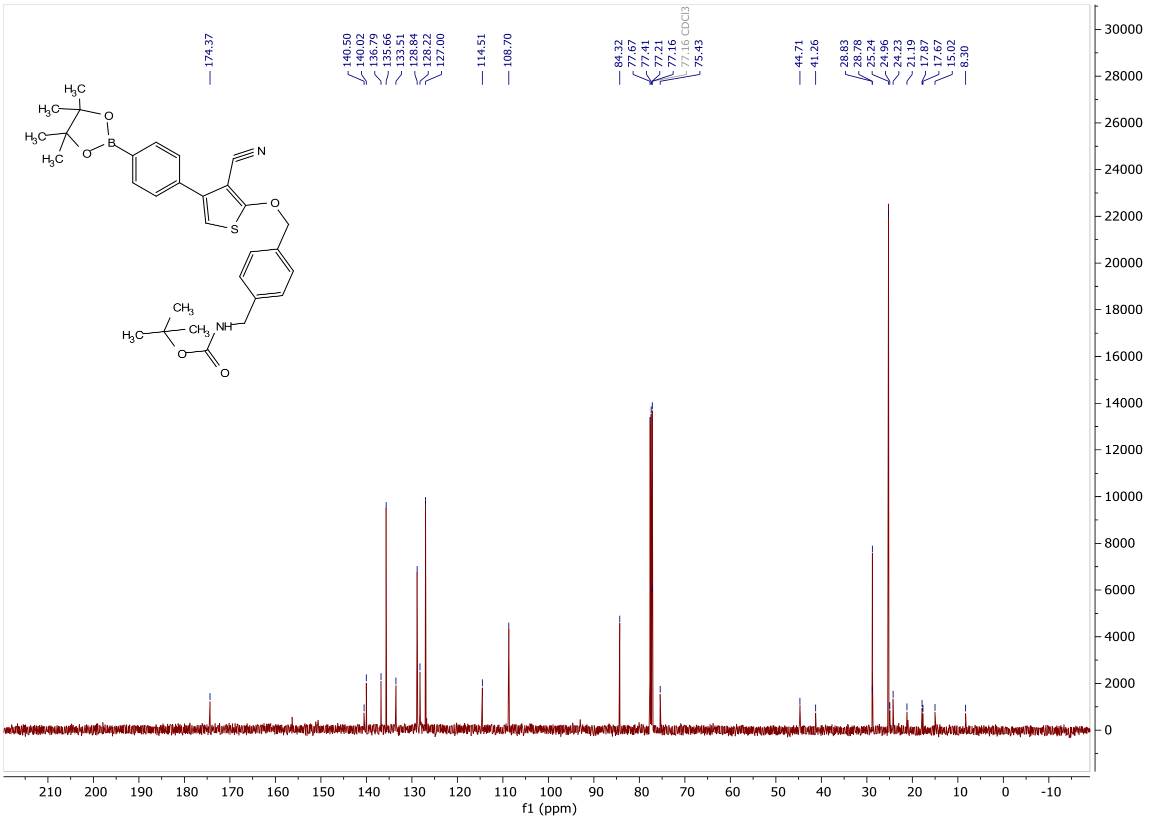

(3-(2,3-dihydrobenzo[b][1,4]dioxin-6-yl)-2-methylphenyl)methanol ***5***

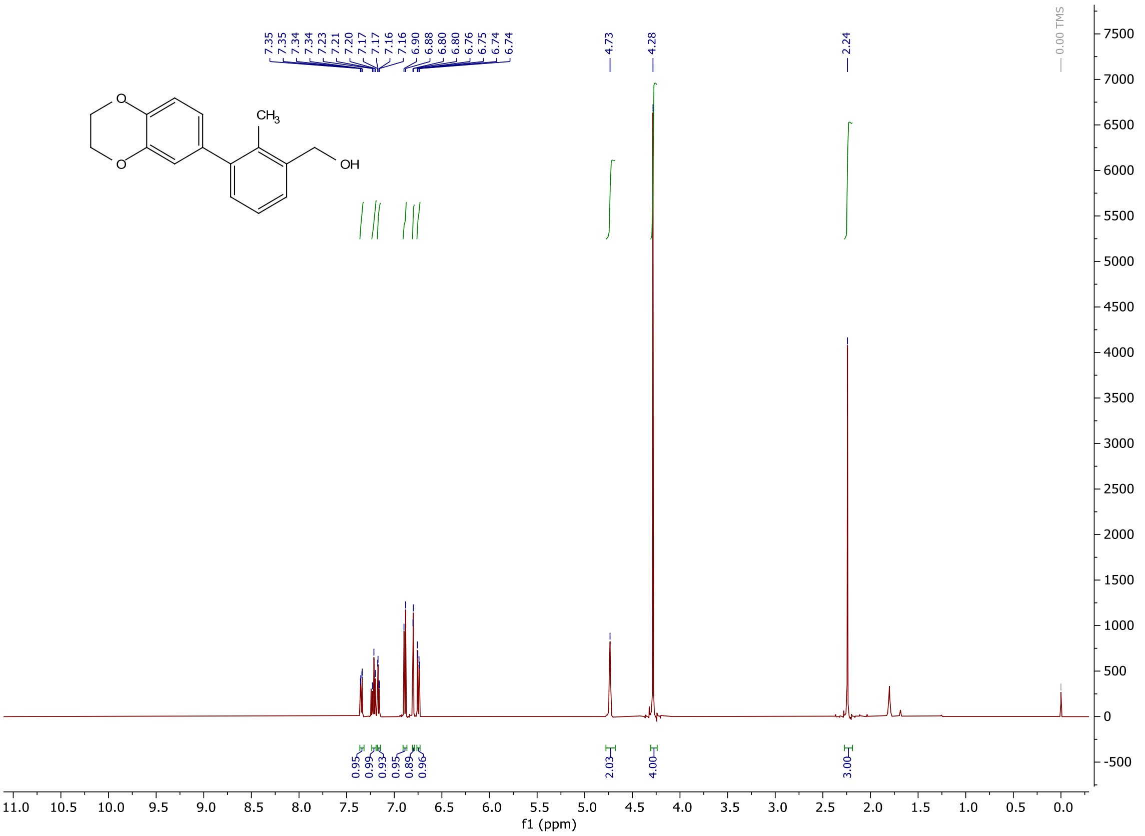

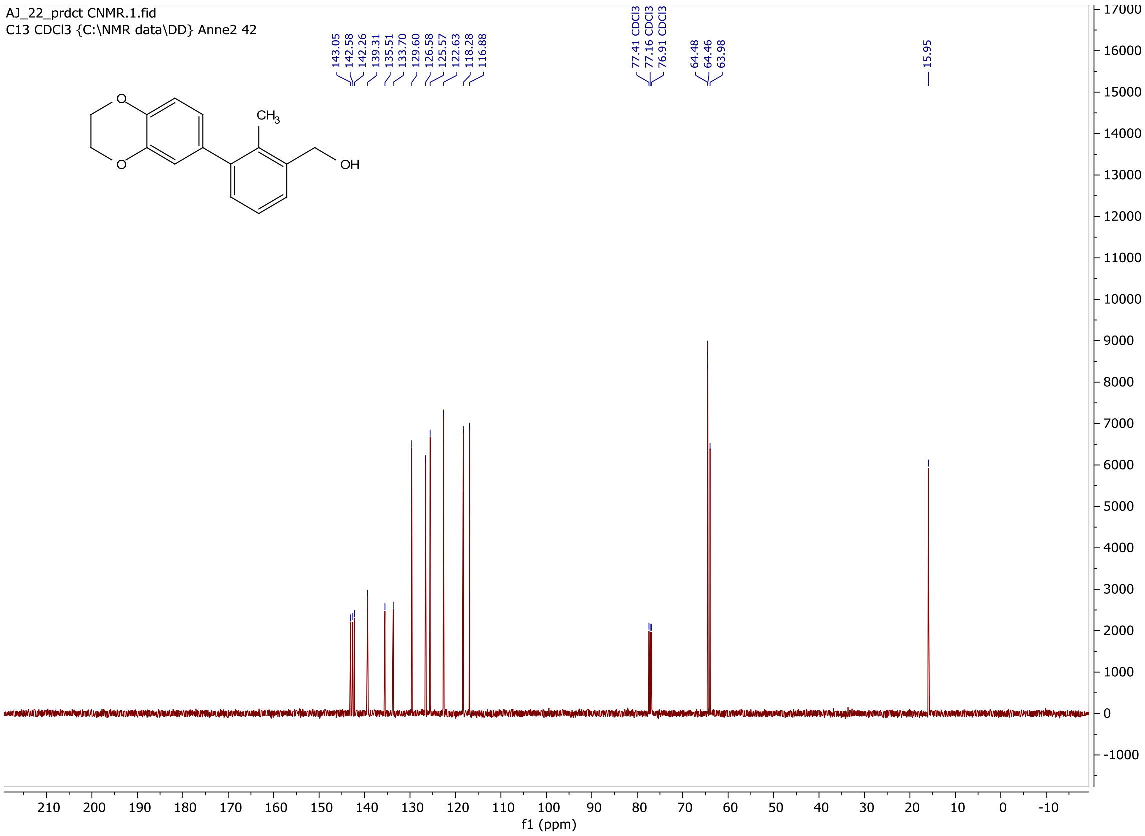

6-(3-(chloromethyl)-2-methylphenyl)-2,3-dihydrobenzo[b][1,4]dioxine ***6***

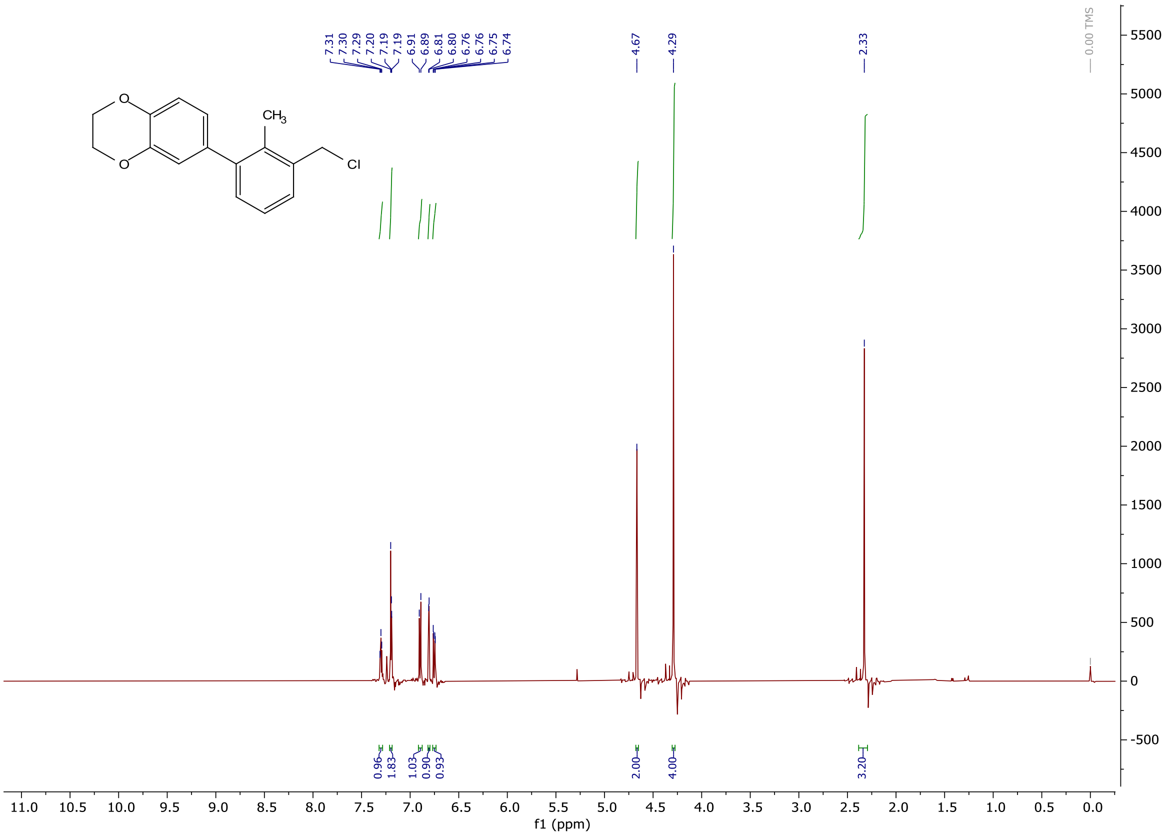

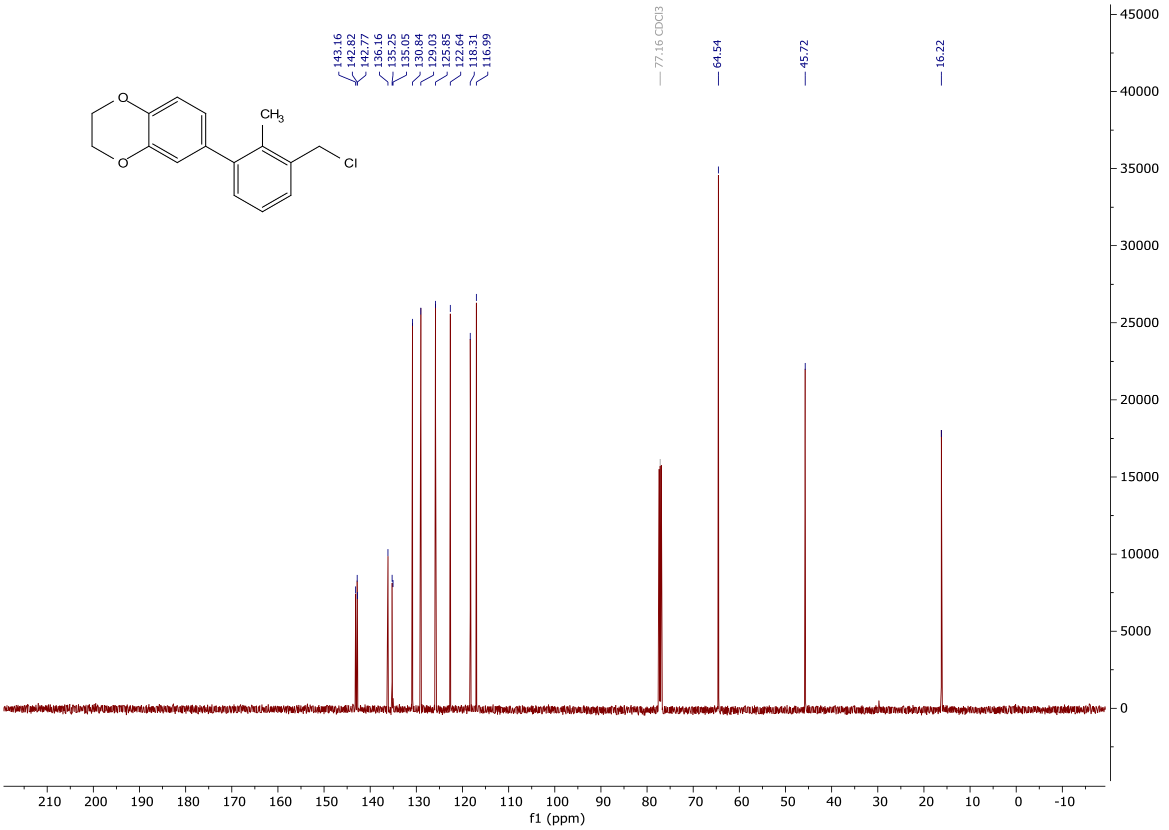

5-chloro-4-((3-(2,3-dihydrobenzo[b][1,4]dioxin-6-yl)-2-methylbenzyl)oxy)-2-hydroxybenzaldehyde ***7***

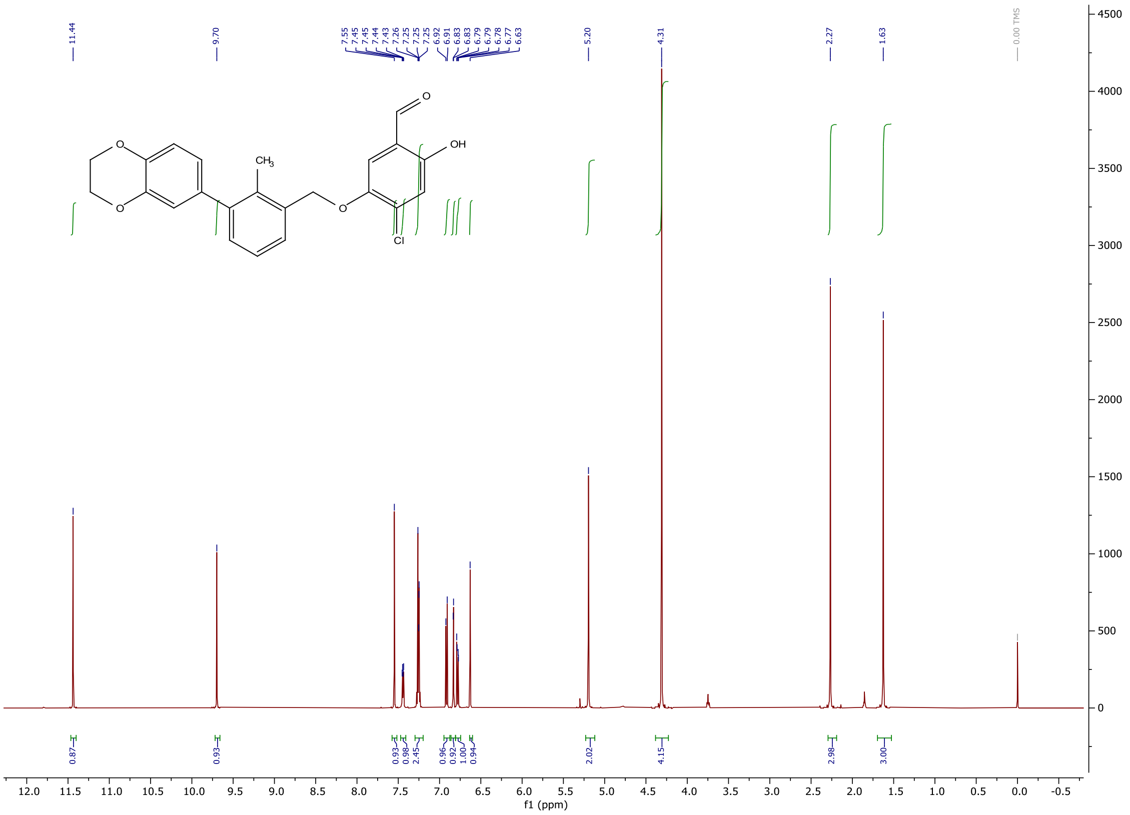

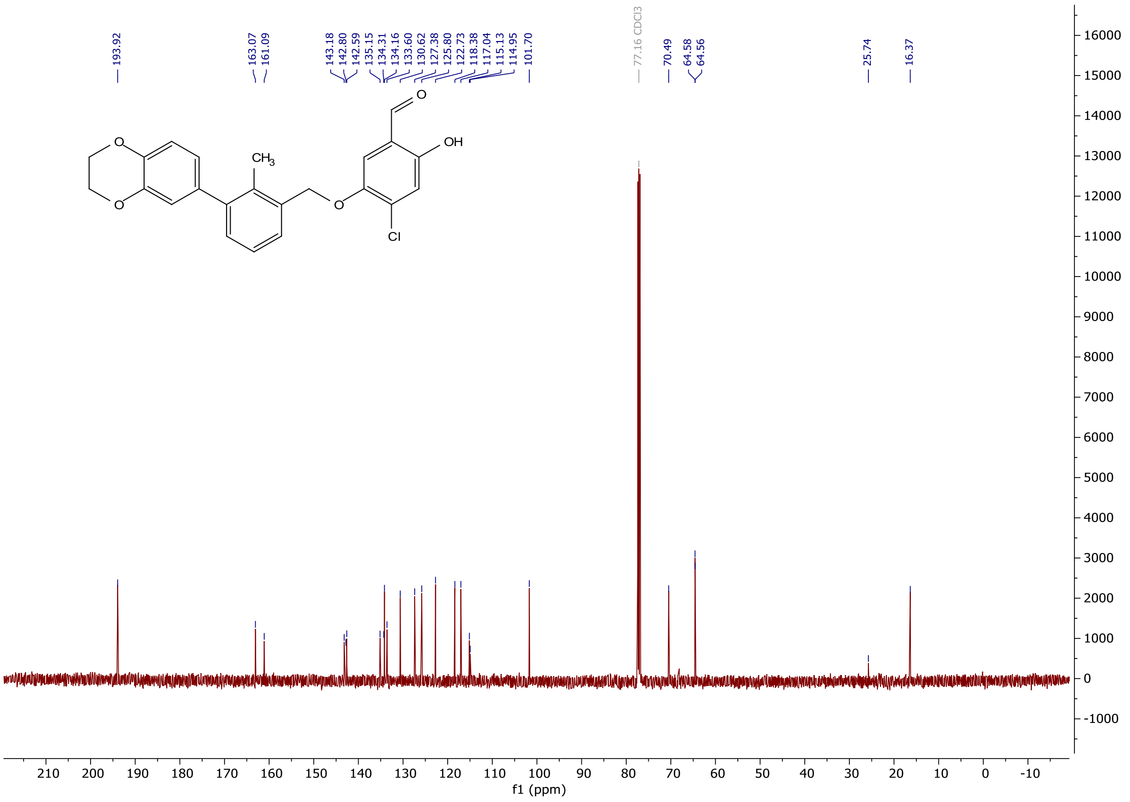

3-((4-chloro-5-((3-(2,3-dihydrobenzo[b][1,4]dioxin-6-yl)-2-methylbenzyl)oxy)-2-formylphenoxy)methyl)benzonitrile ***8***

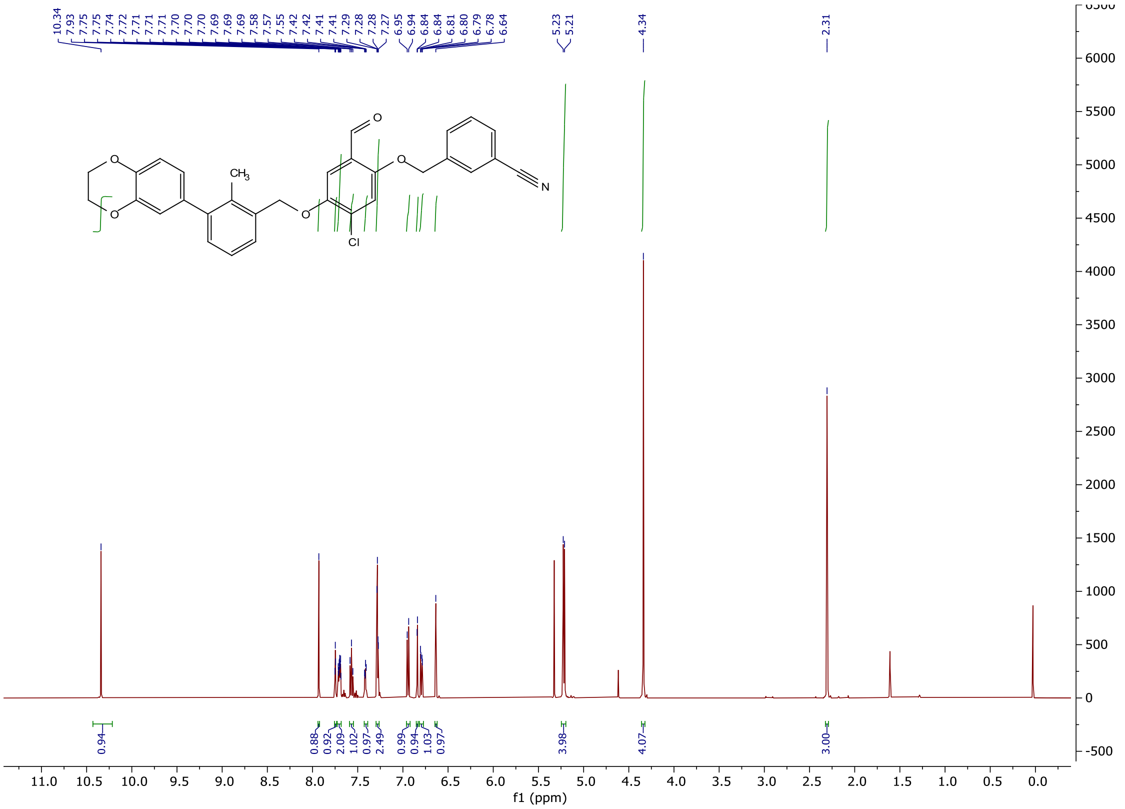

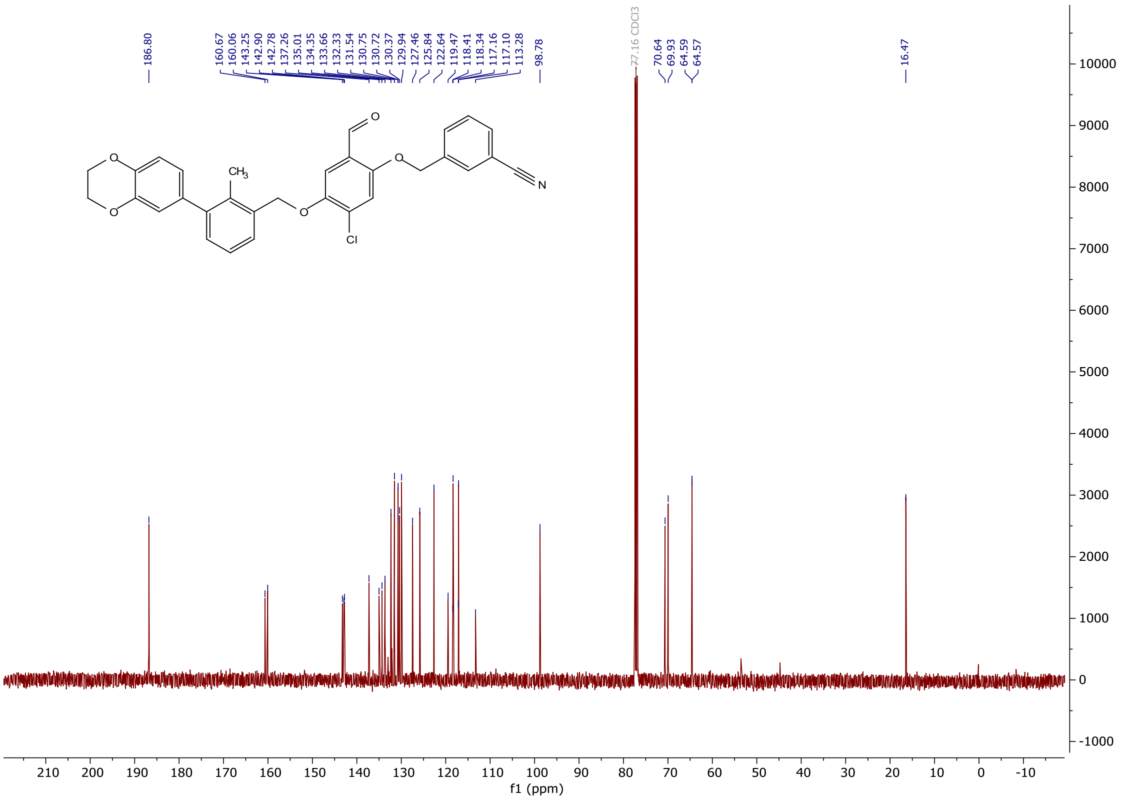

(2R,4S)-1-(5-chloro-2-((3-cyanobenzyl)oxy)-4-((3-(2,3-dihydrobenzo[b][1,4]dioxin-6-yl)-2-methylbenzyl)oxy)benzyl)-4-fluoropyrrolidine-2-carboxylic acid ***9a***

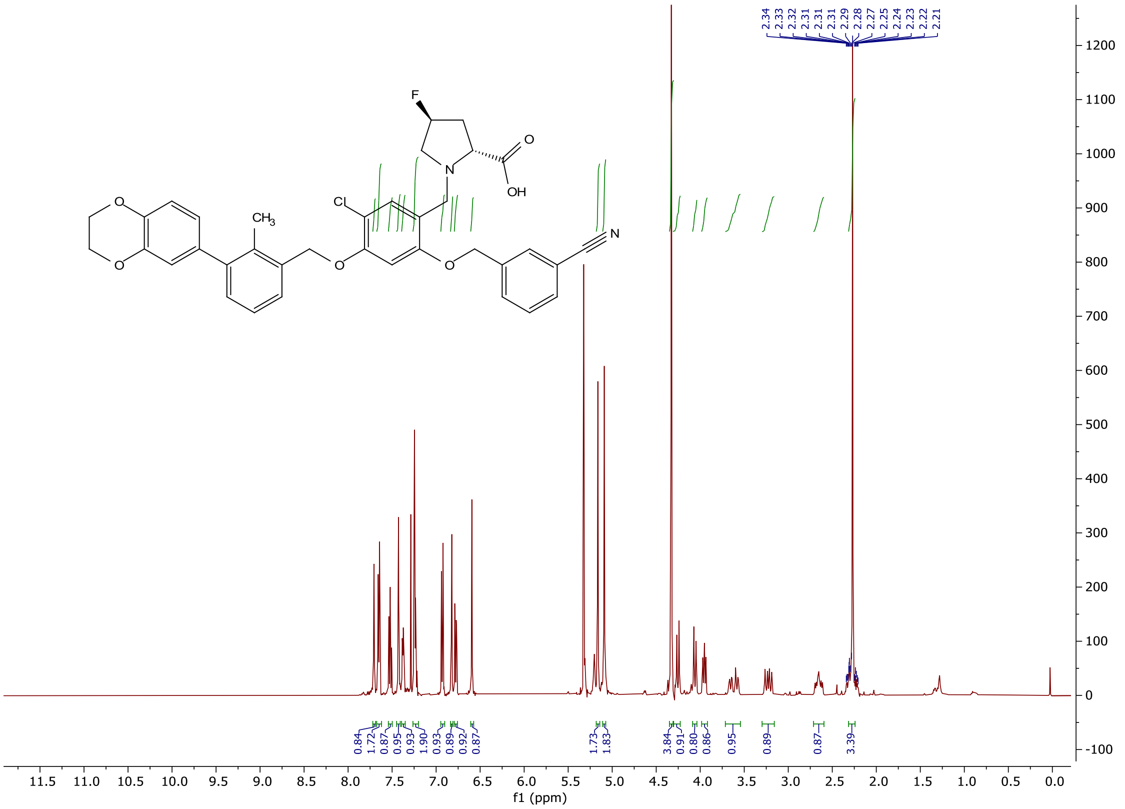

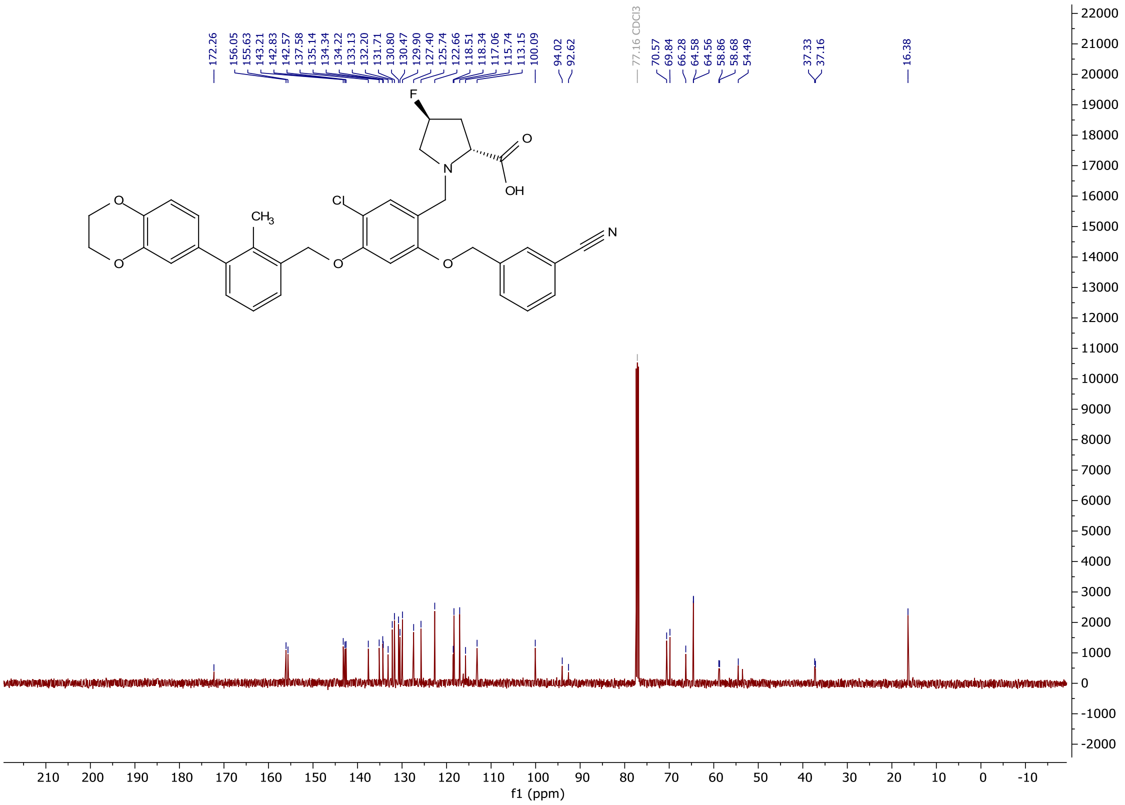

(2R,4R)-1-(5-chloro-2-((3-cyanobenzyl)oxy)-4-((3-(2,3-dihydrobenzo[b][1,4]dioxin-6-yl)-2-methylbenzyl)oxy)benzyl)-4-hydroxypyrrolidine-2-carboxylic acid ***9b***

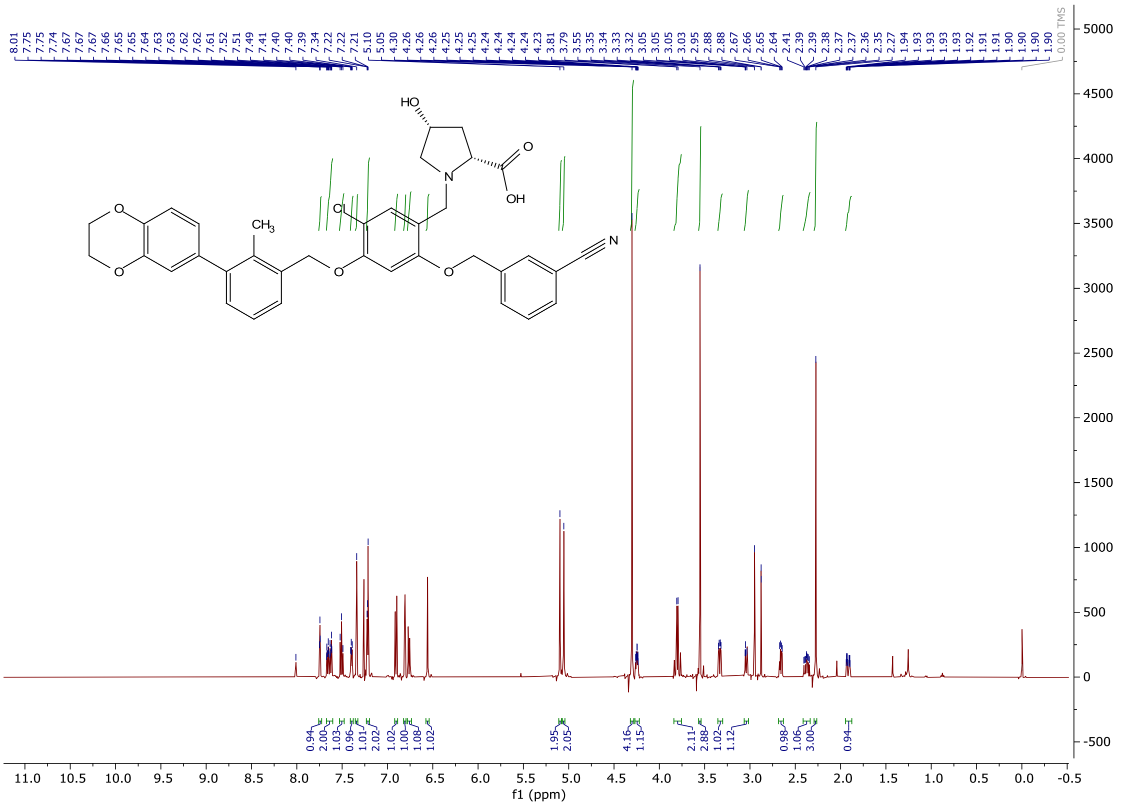

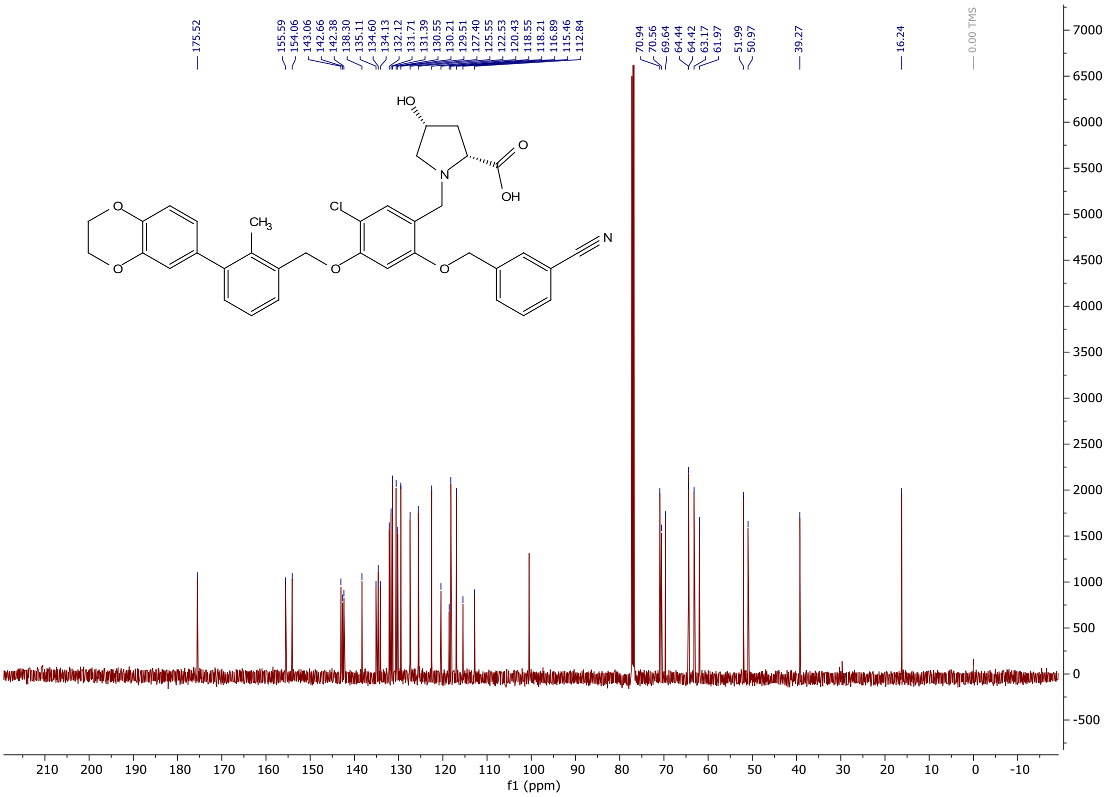

Methyl (2R,4R)-1-(5-chloro-2-((3-cyanobenzyl)oxy)-4-((3-(2,3-dihydrobenzo[b][1,4]dioxin-6-yl)-2-methylbenzyl)oxy)benzyl)-4-hydroxypyrrolidine-2-carboxylate ***9c***

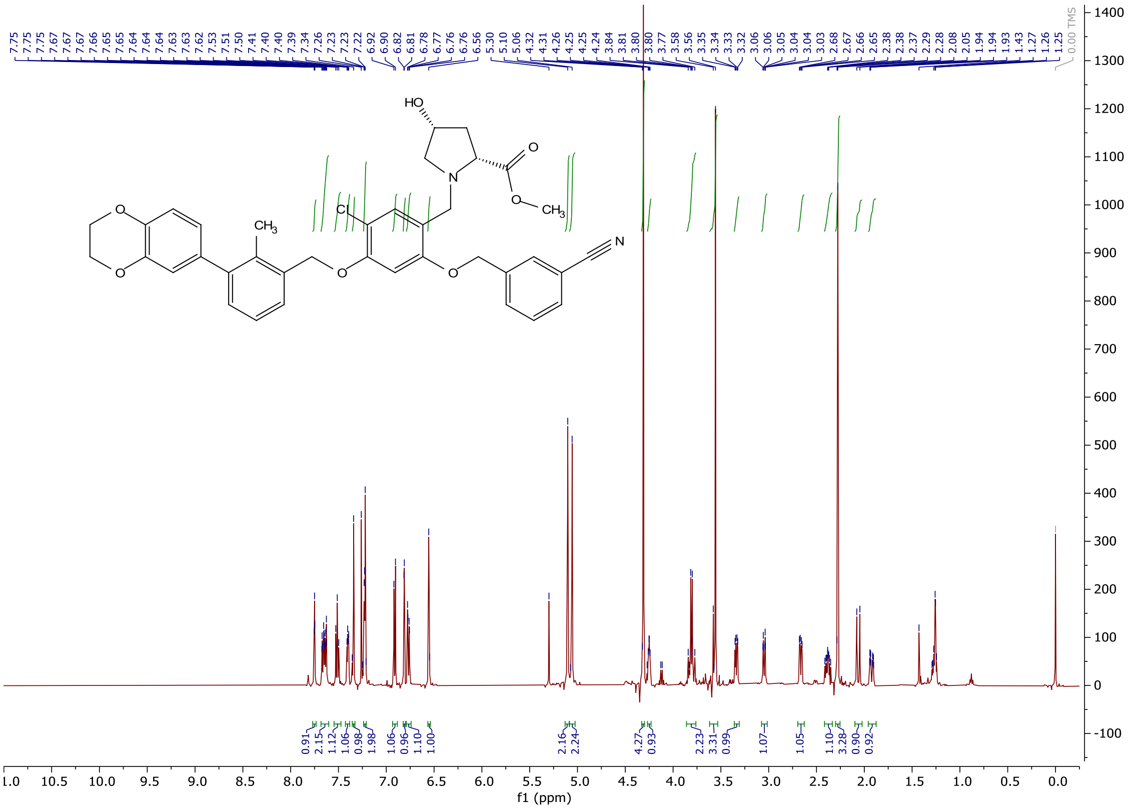

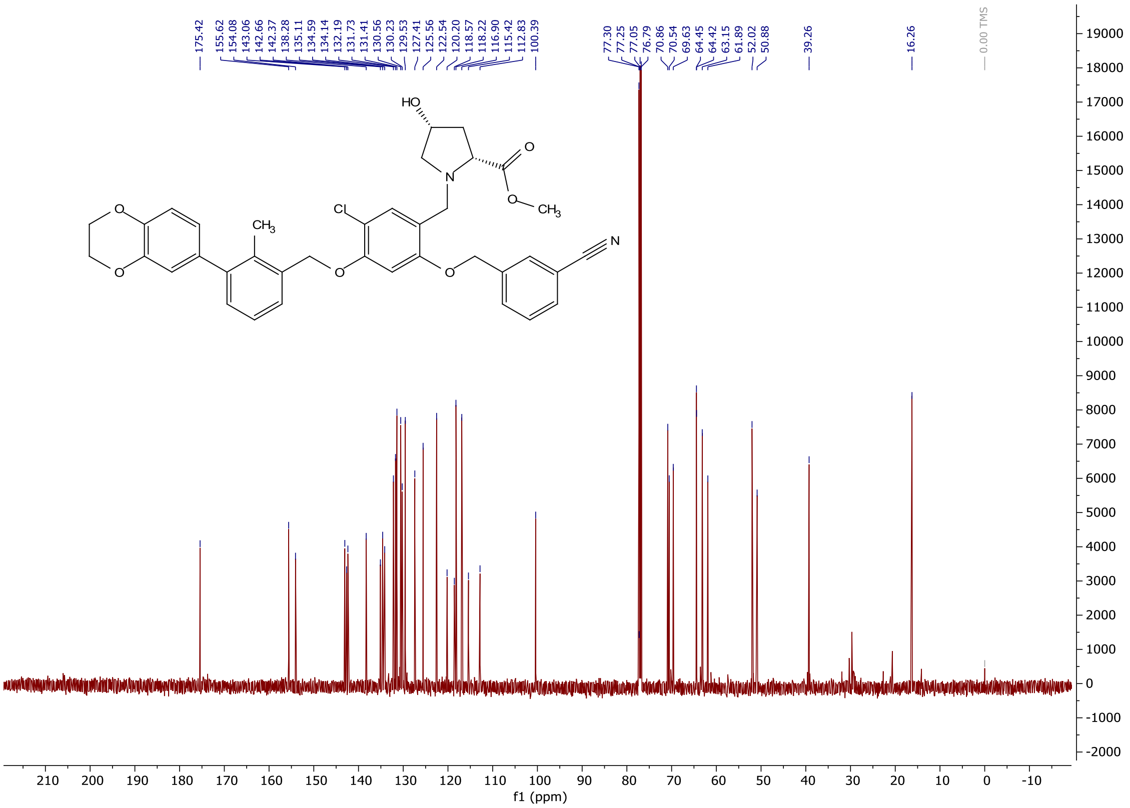

Methyl (2R,4R)-1-(5-chloro-2-((3-cyanobenzyl)oxy)-4-((3-(2,3-dihydrobenzo[b][1,4]dioxin-6-yl)-2-methylbenzyl)oxy)benzyl)-4-(tosyloxy)pyrrolidine-2-carboxylate ***10***

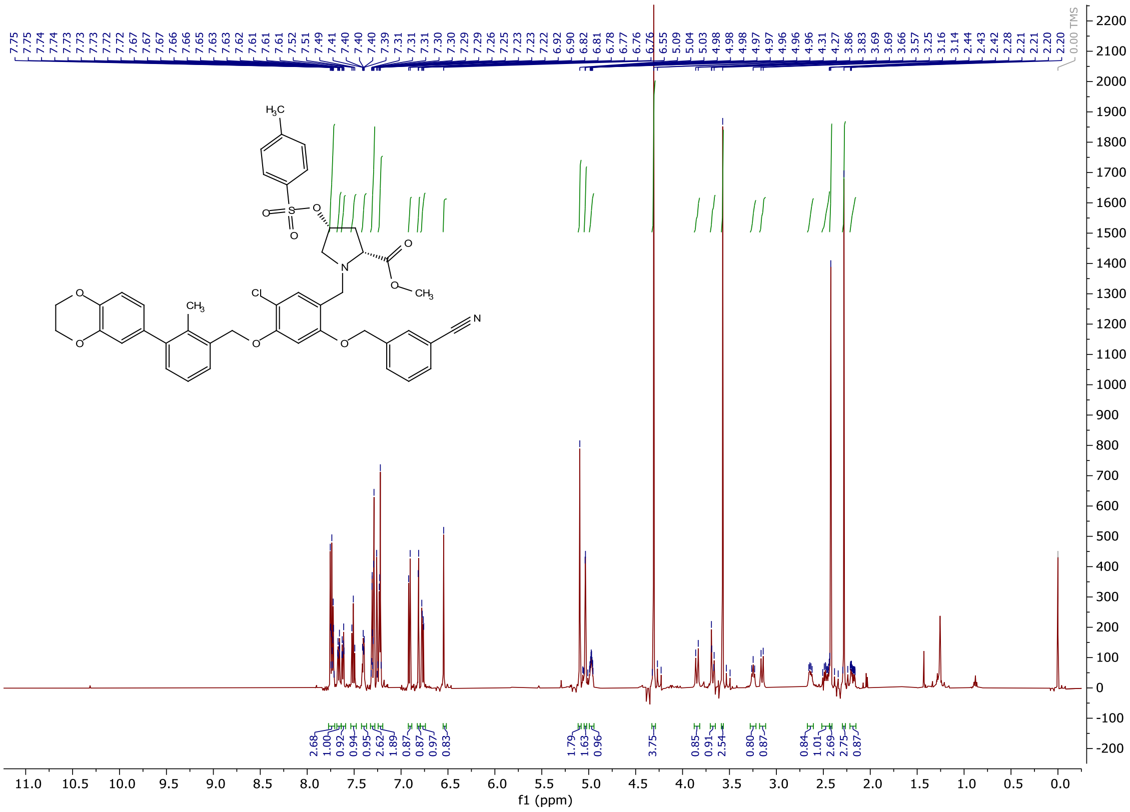

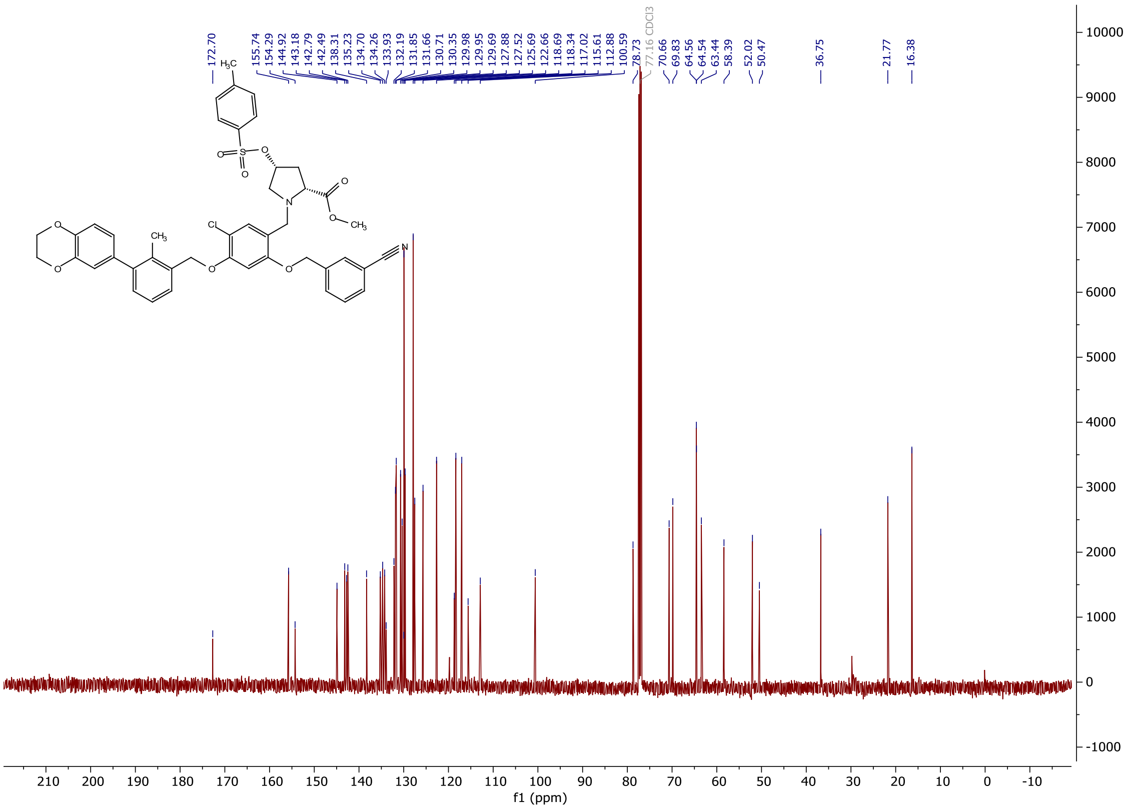

HRMS

*Ethyl 2-amino-4-(4-fluorophenyl)thiophene-3-carboxylate **1a***

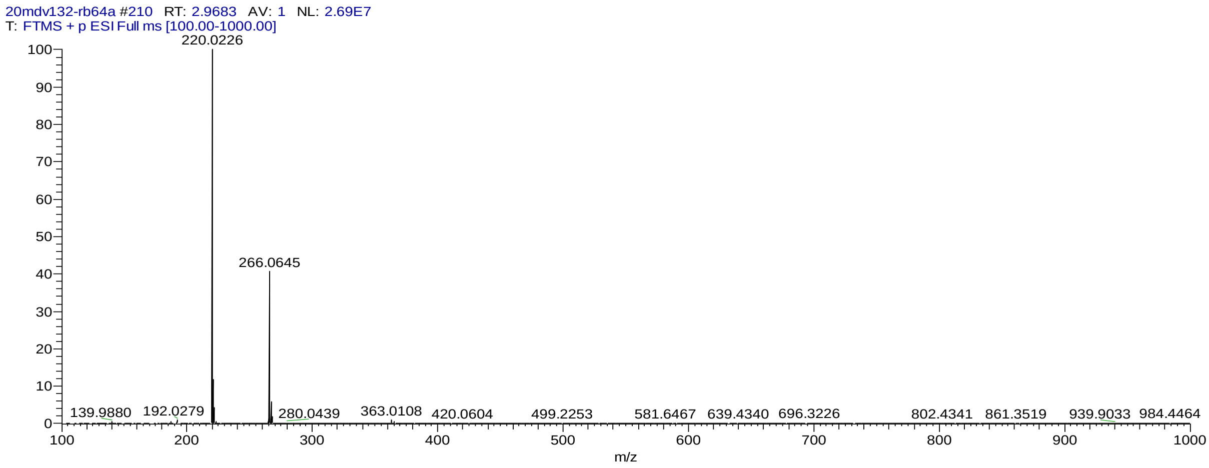

*Ethyl 2-amino-4-(4-bromophenyl)thiophene-3-carboxylate **1b***

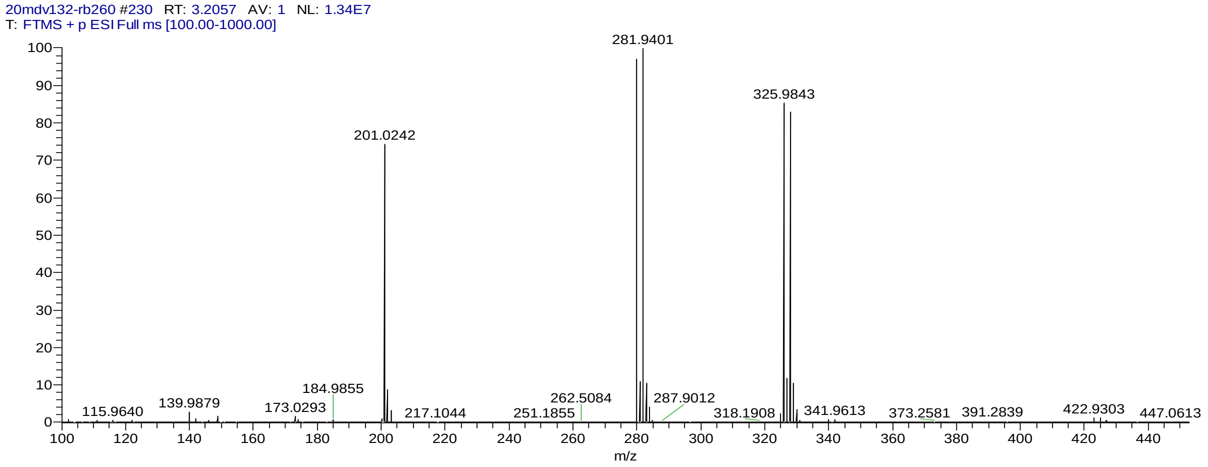

3-(2,3-dihydrobenzo[b][1,4]dioxin-6-yl)-2-methylphenyl)methanol ***5***

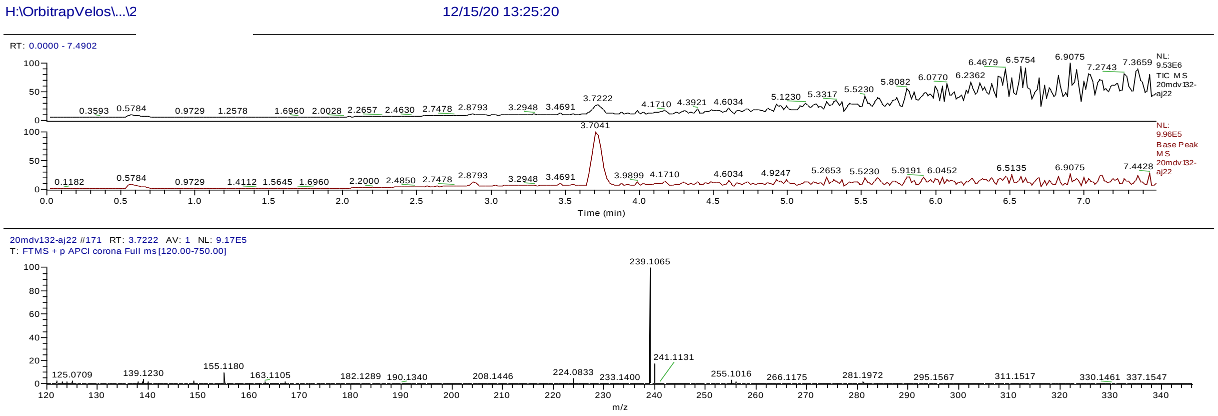

6-(3-(chloromethyl)-2-methylphenyl)-2,3-dihydrobenzo[b][1,4]dioxine ***6***

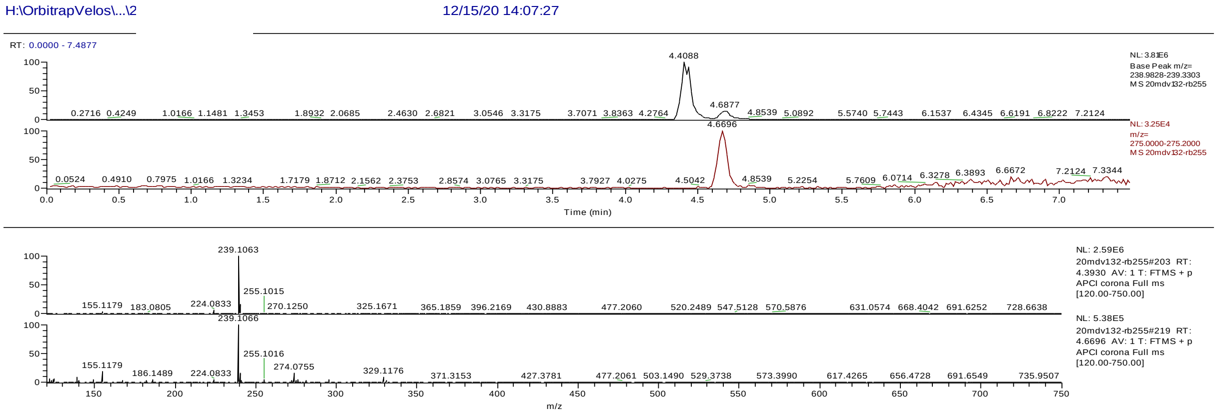

5-chloro-4-((3-(2,3-dihydrobenzo[b][1,4]dioxin-6-yl)-2-methylbenzyl)oxy)-2-hydroxybenzaldehyde ***7***

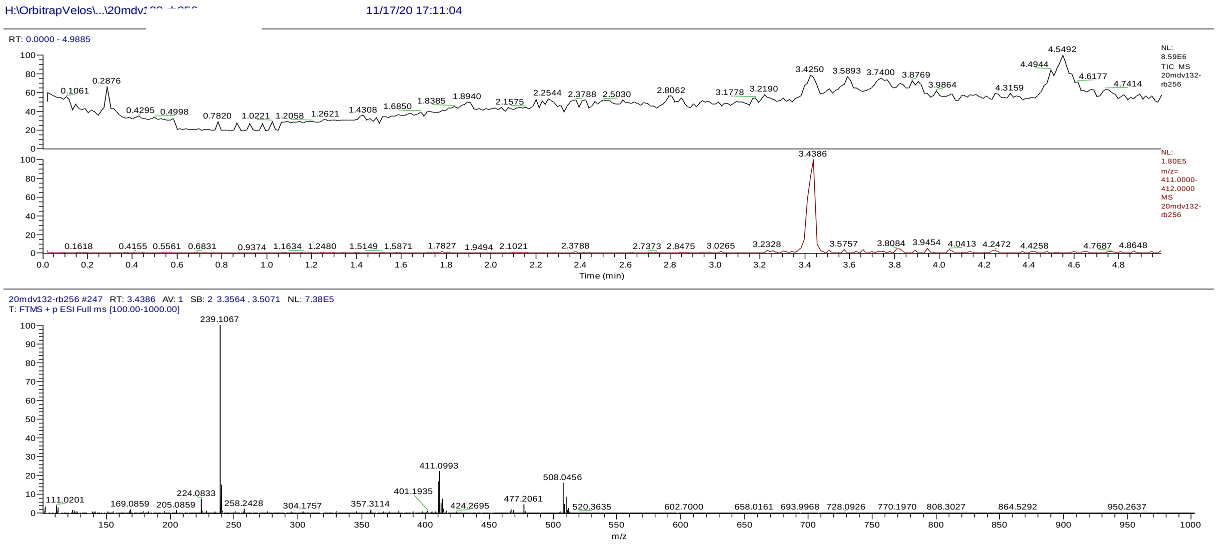

(2R,4S)-1-(5-chloro-2-((3-cyanobenzyl)oxy)-4-((3-(2,3-dihydrobenzo[b][1,4]dioxin-6-yl)-2-methylbenzyl)oxy)benzyl)-4-fluoropyrrolidine-2-carboxylic acid ***9a***

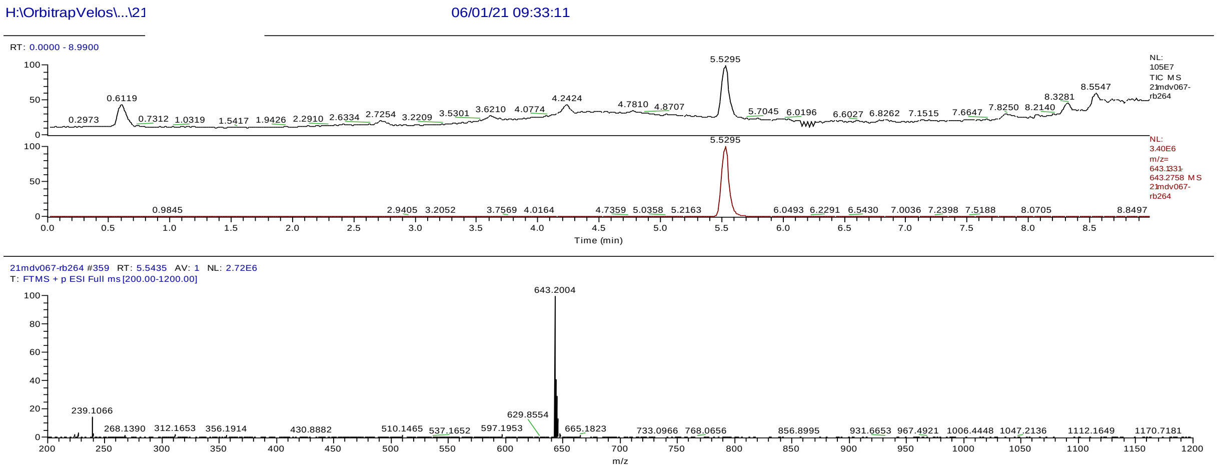

(2R,4S)-1-(5-chloro-2-((3-cyanobenzyl)oxy)-4-((3-(2,3-dihydrobenzo[b][1,4]dioxin-6-yl)-2-methylbenzyl)oxy)benzyl)-4-hydroxypyrrolidine-2-carboxylic acid ***9b***

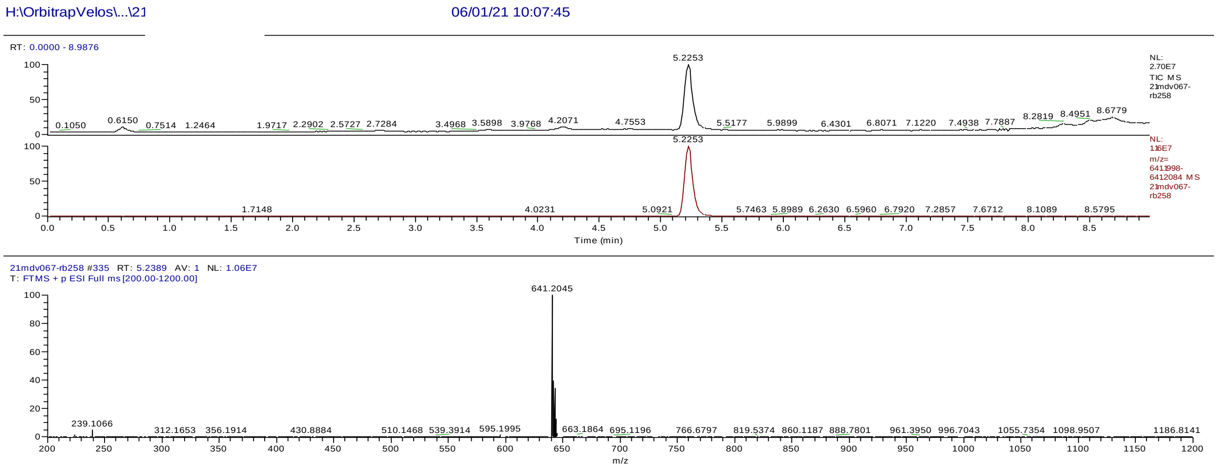

methyl (2R,4R)-1-(5-chloro-2-((3-cyanobenzyl)oxy)-4-((3-(2,3-dihydrobenzo[b][1,4]dioxin-6-yl)-2-methylbenzyl)oxy)benzyl)-4-hydroxypyrrolidine-2-carboxylate **9c**

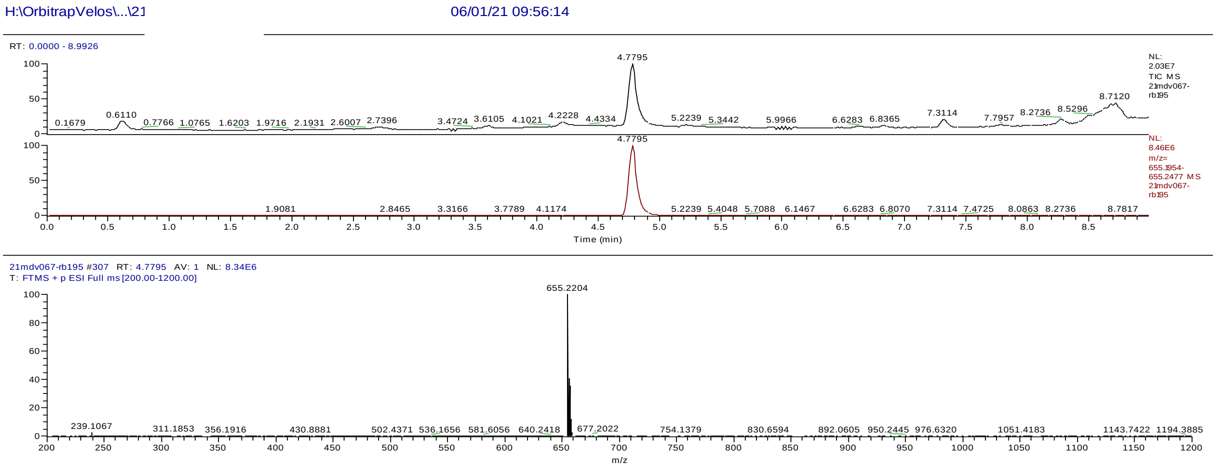

Methyl (2R,4R)-1-(5-chloro-2-((3-cyanobenzyl)oxy)-4-((3-(2,3-dihydrobenzo[b][1,4]dioxin-6-yl)-2-methylbenzyl)oxy)benzyl)-4-(tosyloxy)pyrrolidine-2-carboxylate **10**

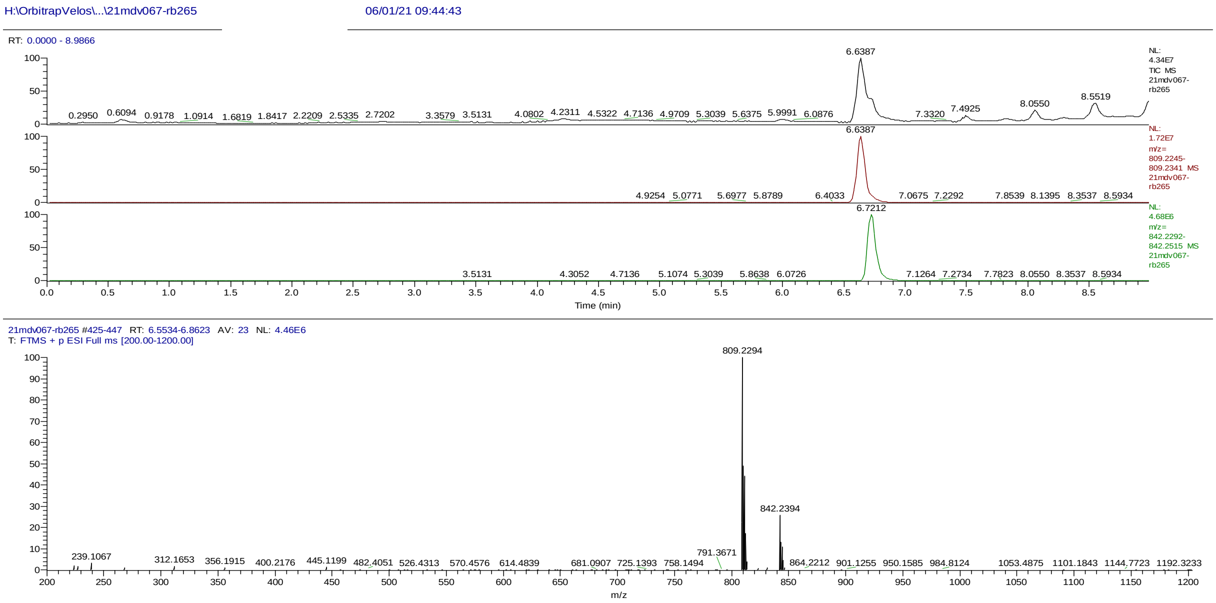

UPLC

RP-UPLC using BEH Shield RP18 column and gradient eluent system (25-75% ACN/water + 0.1 %TFA) for **4a** and BEH Phenyl RP18 column and isocratic eluent system (45% ACN/water + 0.1 %TFA) for **9a**.

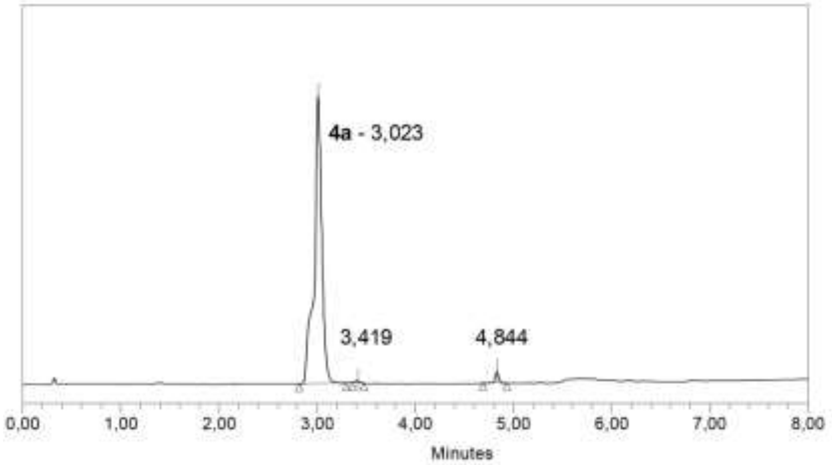

	RT	Area	% Area
1	4,844	28671	1,98
2	3,419	5996	0,41
3	3,023	1411953	97,60

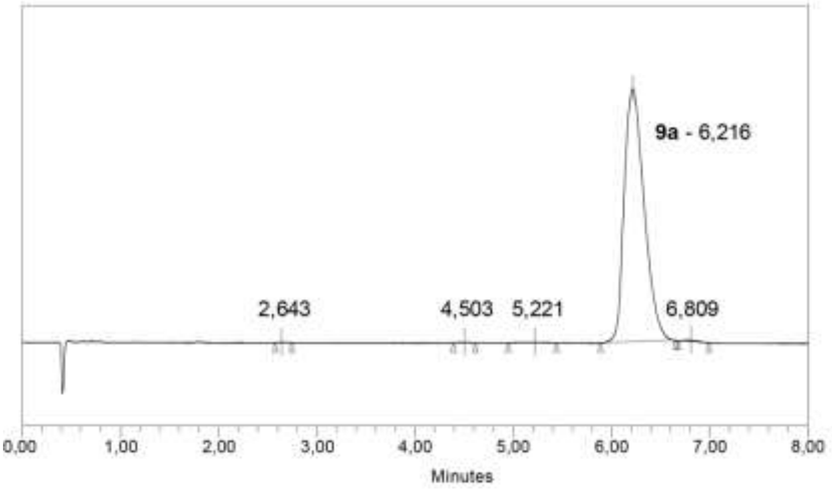

	RT	Height	Area	% Area
1	2,643	37	182	0,10
2	4,503	31	200	0,11
3	6,809	70	653	0,37
4	6,216	12507	176379	99,15
5	5,221	33	486	0,27

Supplementary Figures and Tables

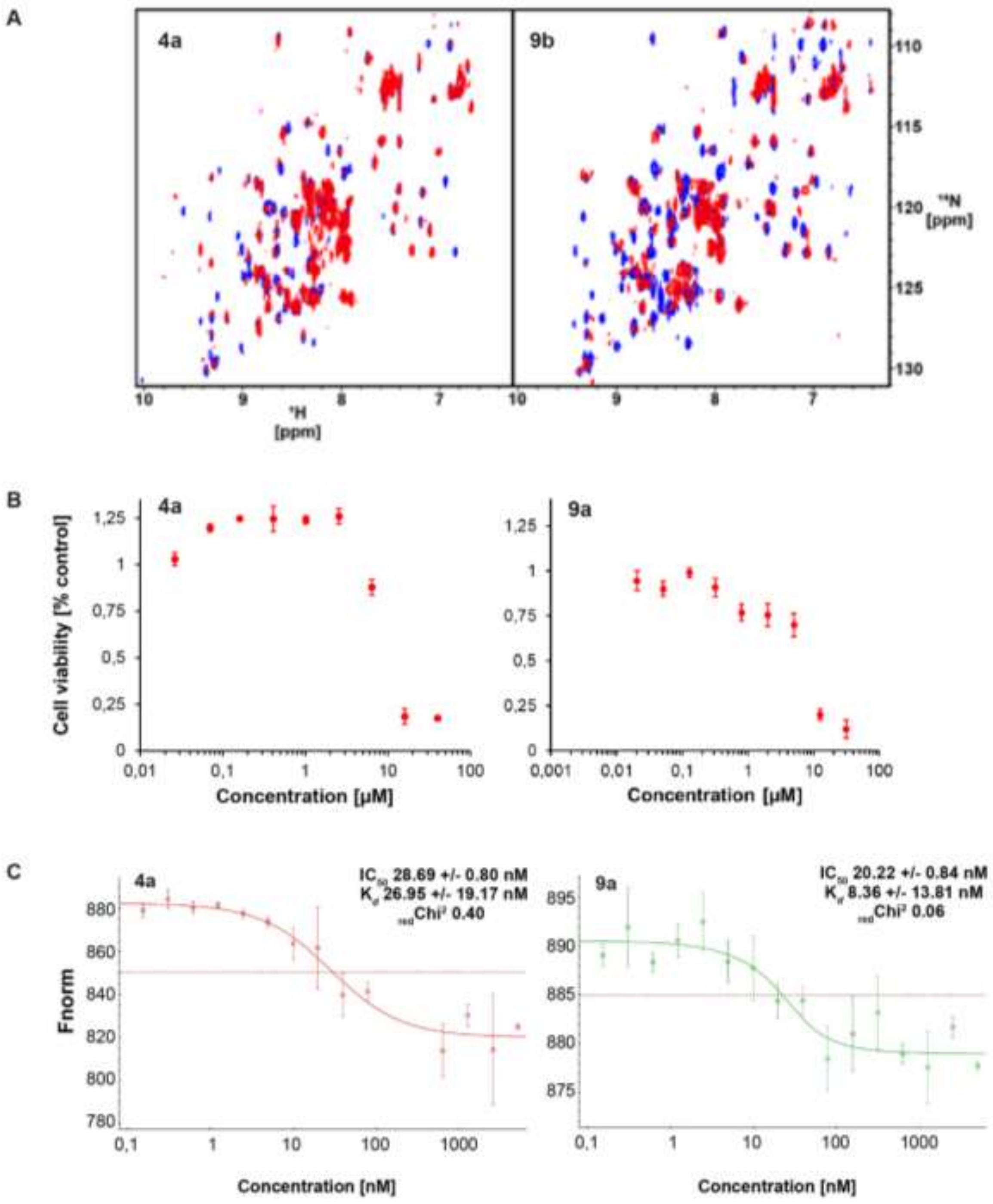

Figure S1. A) ^{1}H-^{15}N HMQC NMR spectra of apo-PD-L1 (blue) and apo-PD-L1 with inhibitor (red): **4a** and **9b** in the molar ratio 1:1, respectively. **B**) Jurkat cell line viability in PD-1/PD-L1 ICB assay with inhibitors **4a** and **9a**. **C**) Affinity analysis of compound **4a** (red) and **9a** (green) to PD-L1. Data was fitted with Kd equation reported in the methodology. n=4**.**

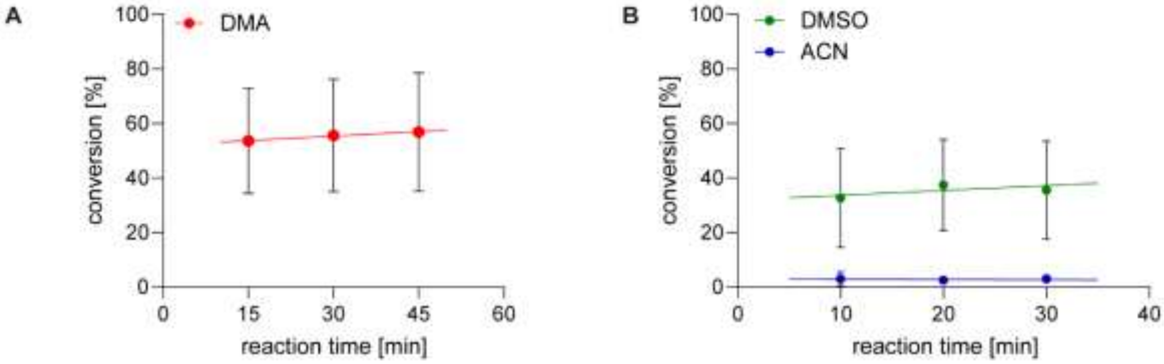

C

tracer	method		duration [min]	RCY [%]	activity [MBq/ml]	amount [ng/ml]
[^{18}F]**4a**	one pot	a	154.3 ± 71.4	8.9 ± 8.6	10.7 ± 5.2	70.7 ± 8.7
[^{18}F]**9a**	A	b	175.0 ± 23.8	0.9 ± 0.5	2.0 ± 1.6	541.5 ± 314.7
	B	c	85.7 ± 7.2	2.5 ± 1.1	29.2 ± 18.0	57.7 ± 48.6

* experiment performed in replicates [a] n=4, [b] n=5, [c] n=6.

Figure S2. Labeling optimization of [^{18}F]F-labeled tracers targeting PD-L1. **A**) [^{18}F]F- conversion into [^{18}F]**4a** measured by TLC (30% EA/hexane) and autoradiography at different timepoint of fluorination reaction; n=3. **B**) [^{18}F]F^- conversion into [^{18}F]**9a** measured by TLC (40% EA/hexane) and autoradiography at different timepoint of fluorination reaction. Two solvents were used, due to low conversion; n=7 (DMSO) and n=3 (ACN). **C**) Characteristic of labeling optimization for [^{18}F]**4a** and [^{18}F]**9a** (two methods).

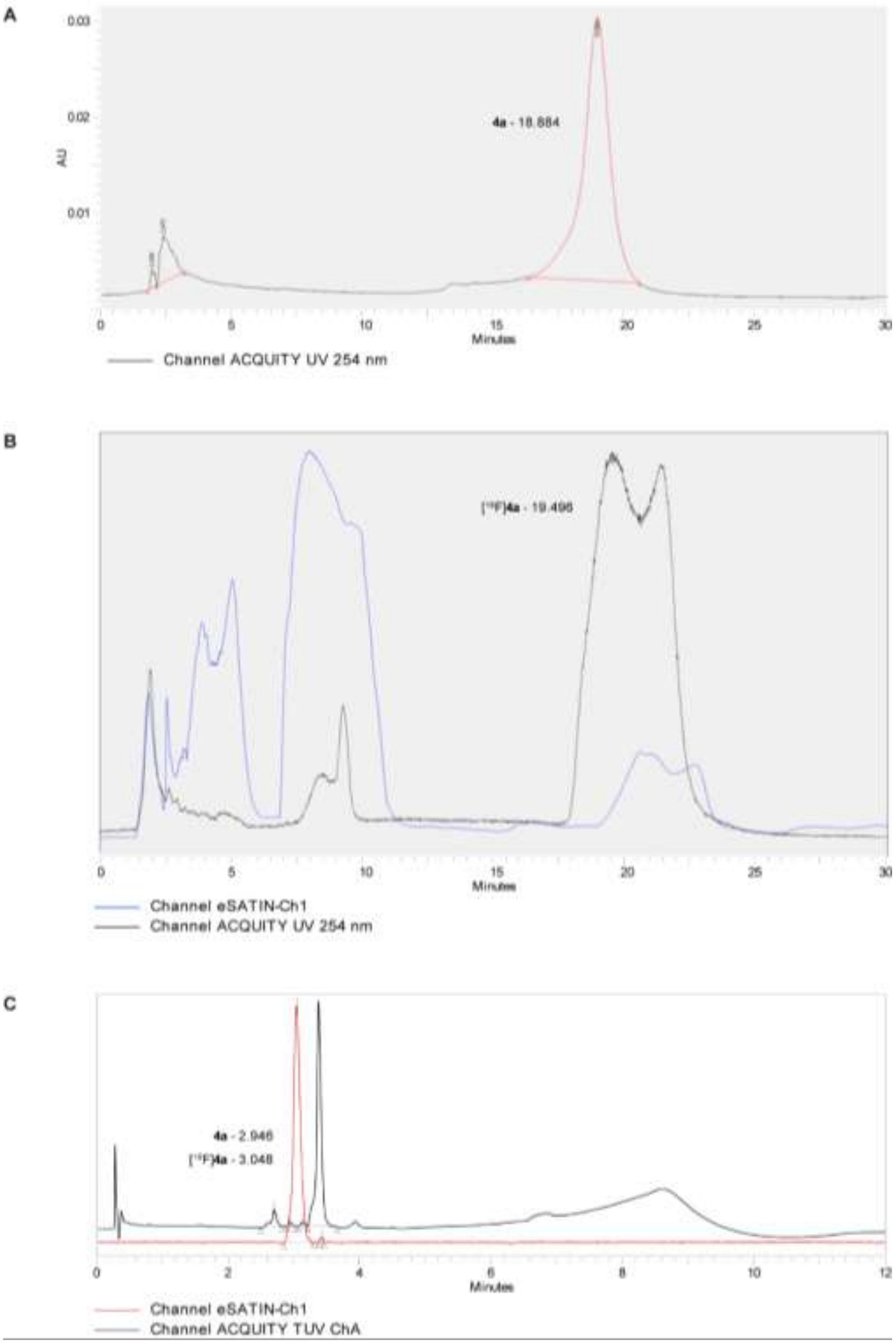

Figure S3. Representative chromatograms used for [^{18}F]**4a** purification and characterization. **A**-**B**) RP-HPLC performed with XBridge BEH Shield OBD Prep C18 column and isocratic eluent system (40% ACN/25mM PBS) for **4a** reference compound (**A**) and [^{18}F]**4a** purification (**B**). **C**) RP-UPLC quality control with BEH Shield RP18 column and gradient eluent system (25-75% ACN/water + 0.1 %TFA).

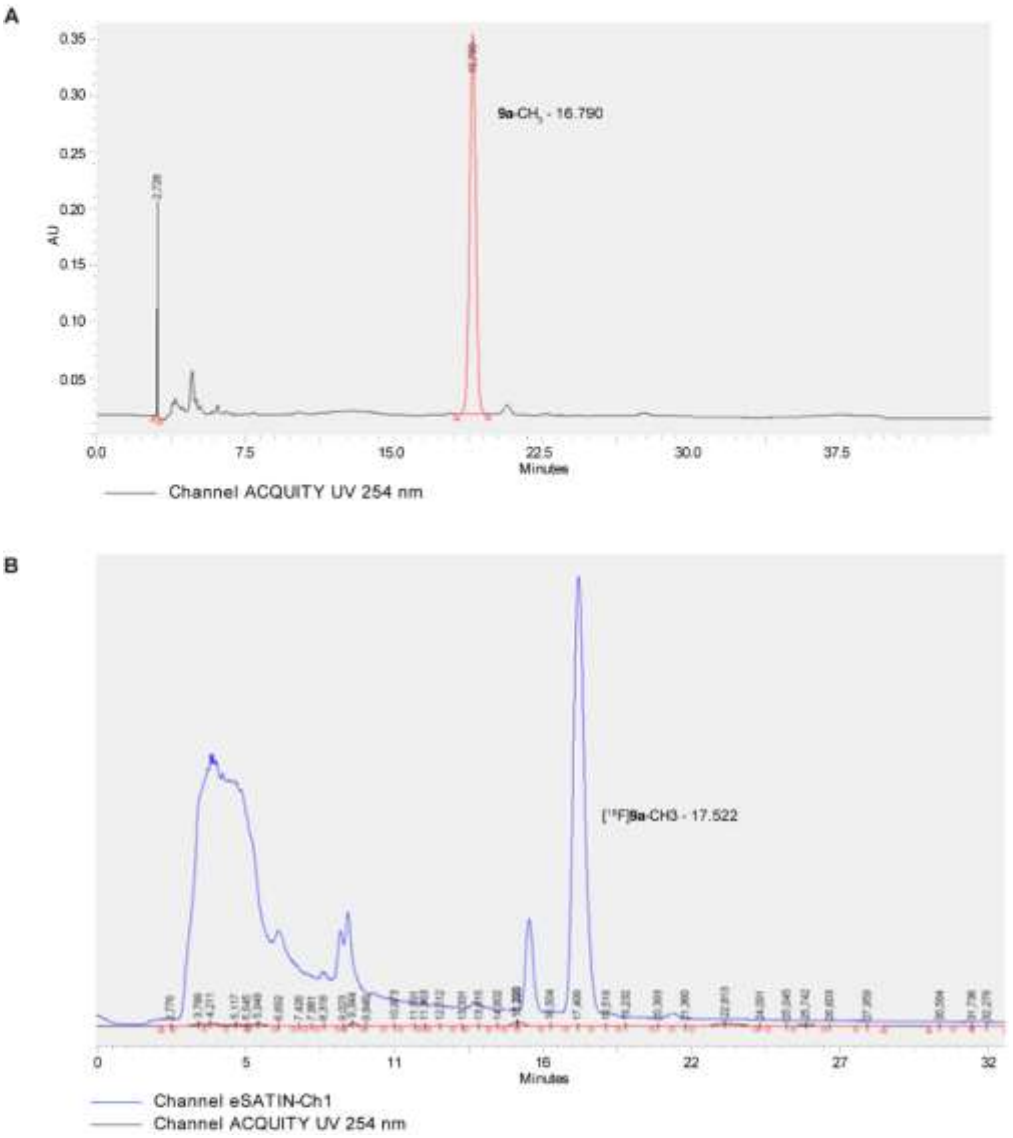

Figure S4. Representative chromatograms used for [^{18}F]**9a** purification and characterization. RP-HPLC system used for a 2-step synthesis method performed with XBridge BEH Shield OBD Prep C18 column and isocratic eluent system (75% ACN/0.1M NaOAc buffer) for **9a**-CH_3 reference compound (**A**), [^{18}F]**9a**-CH_3 purification (**B**).

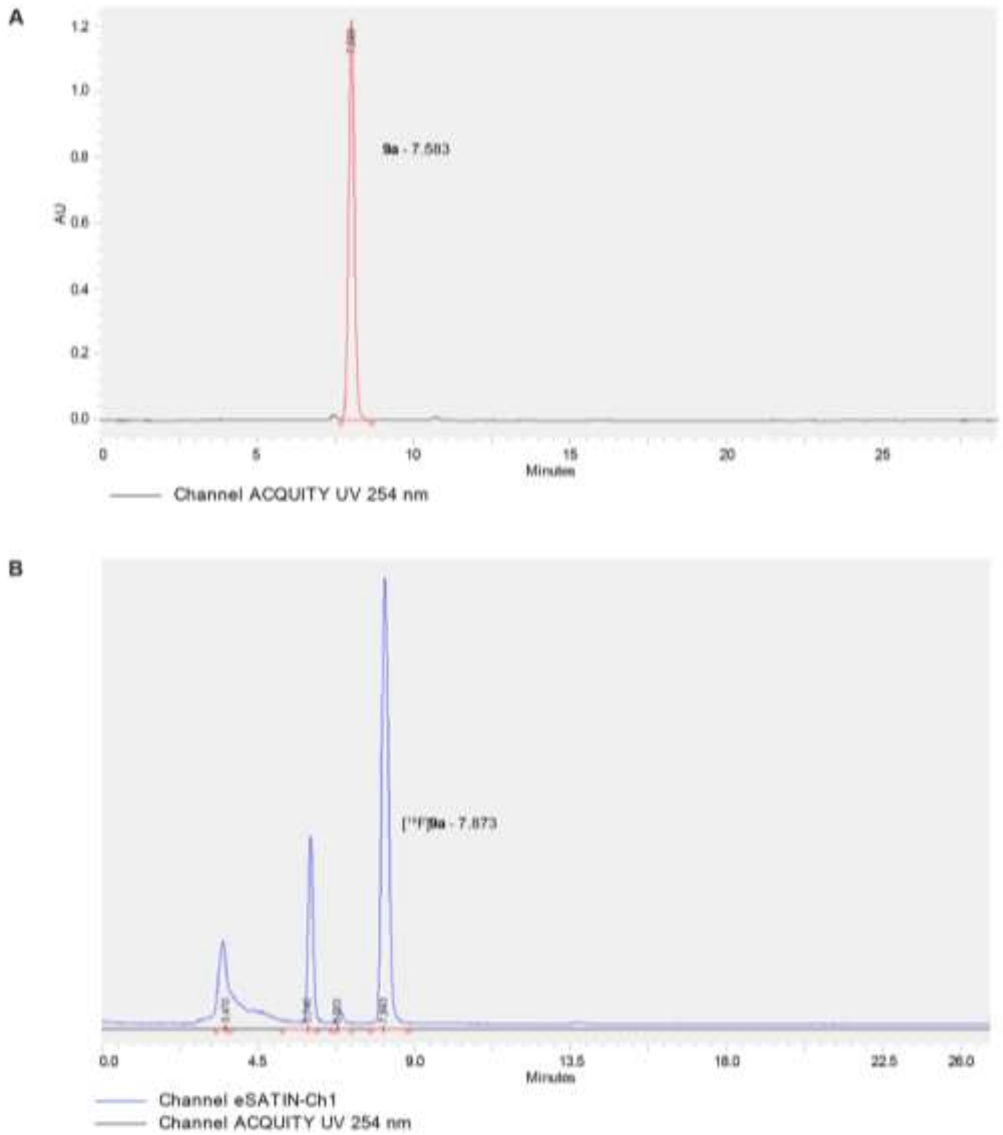

Figure S5. Representative chromatograms used for [^{18}F]**9a** purification and characterization. RP-HPLC system used for a 2-step synthesis method performed with XBridge BEH Shield OBD Prep C18 column and isocratic eluent system (60% ACN/0.1M NaOAc buffer) for **9a** reference compound (**A**) and [^{18}F]**9a** purification (**B**).

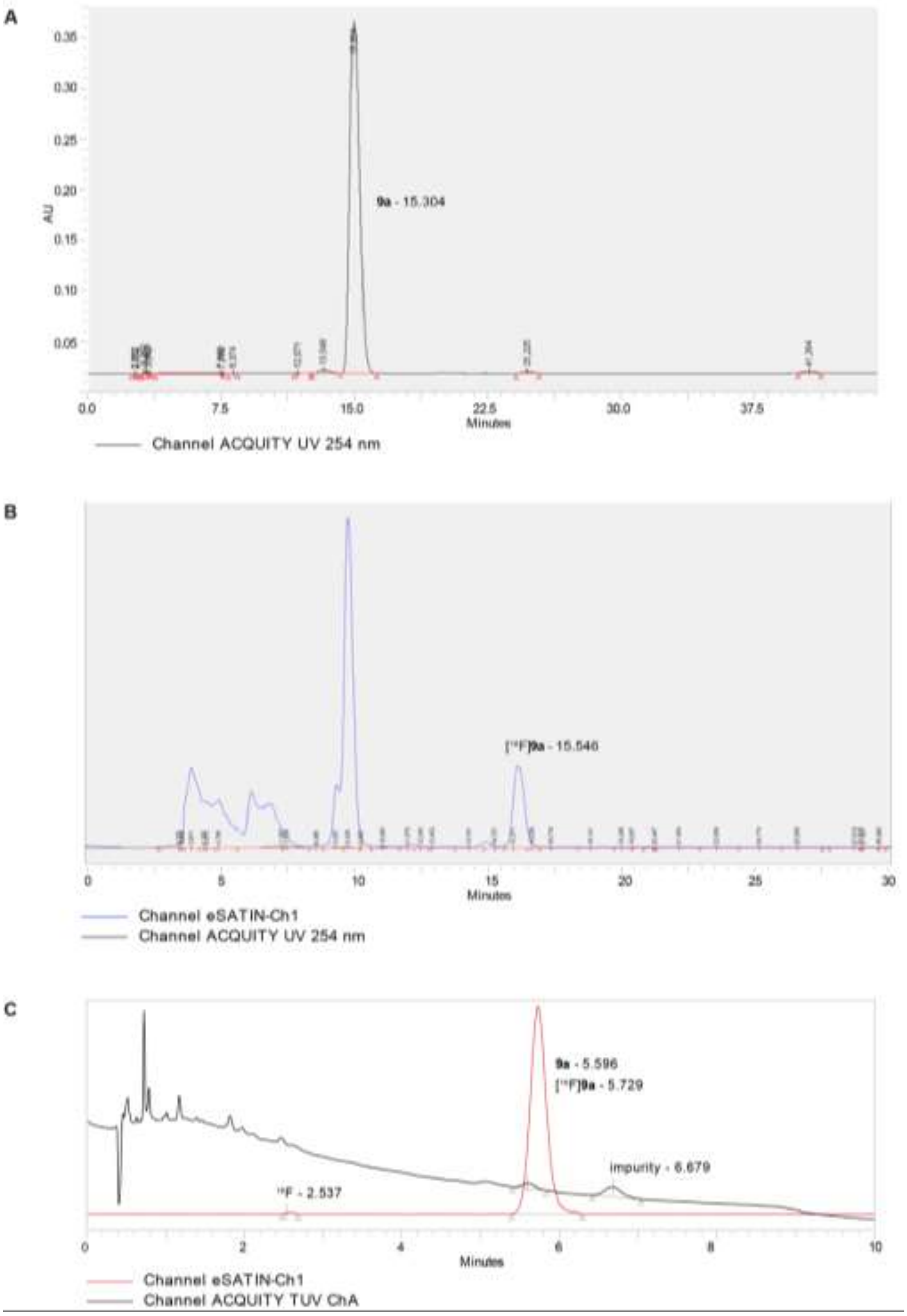

Figure S6. Representative chromatograms used for [18F]**9a** purification and characterization. RP-HPLC system used for one-pot synthesis method performed with XBridge BEH Shield OBD Prep C18 column and isocratic eluent system (50% ACN/0.1M NaOAc buffer) for **9a** reference compound (**A**) and [18F]**9a** purification (**B**). **C**) RP-UPLC quality control with BEH Phenyl RP18 column and isocratic eluent system (45% ACN/water + 0.1 %TFA).

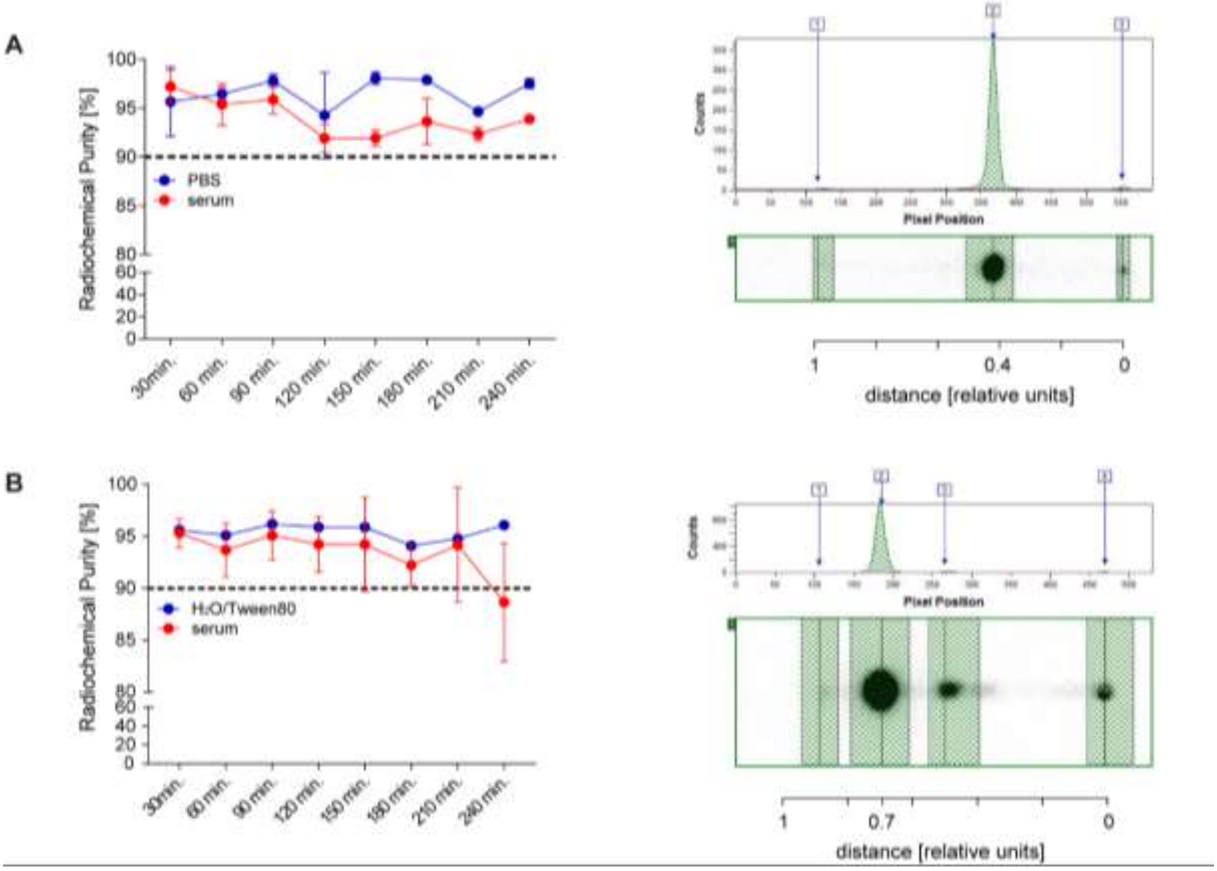

Figure S7. Stability of [^{18}F]F-labeled tracers targeting PD-L1 measured by TLC and visualized by autoradiography. (**A**) [^{18}F]**4a** using 10%MeOH/DCM + NH_3 as eluent, (**B**) [^{18}F]**9a** using 10%MeOH/DCM as eluent.

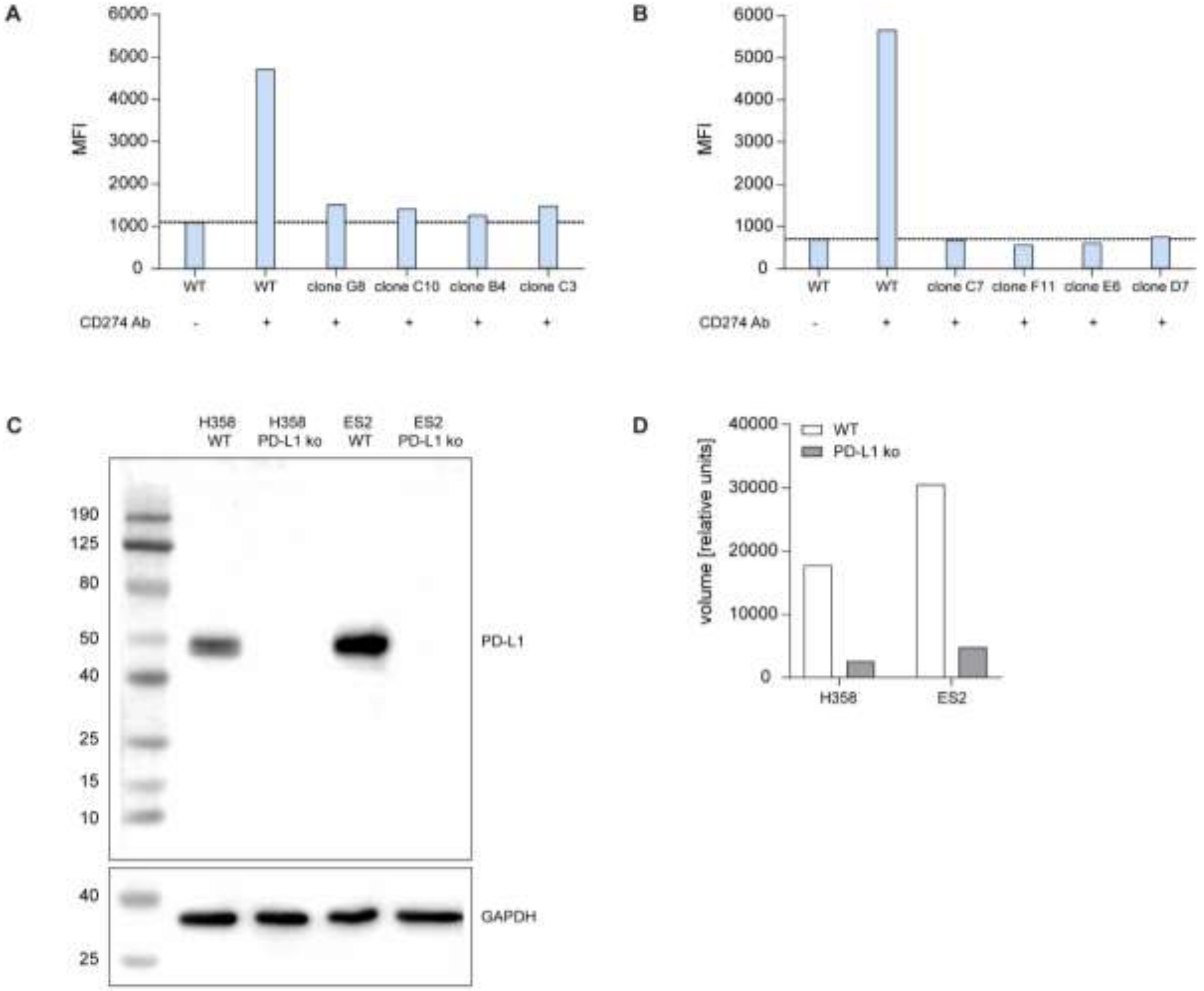

Figure S8. A-**B**) Cell surface PD-L1 expression measured by flow cytometry: H358 PD-L1+/- (**A**) and ES2 PD-L1+/- (**B**). The threshold is set to a wild-type sample without a anti-CD274 antibody. **C**-**D**) Cell intracellular PD-L1 expression measured by western blotting: blot visualization (**C**) and expression levels quantifications (**D**).

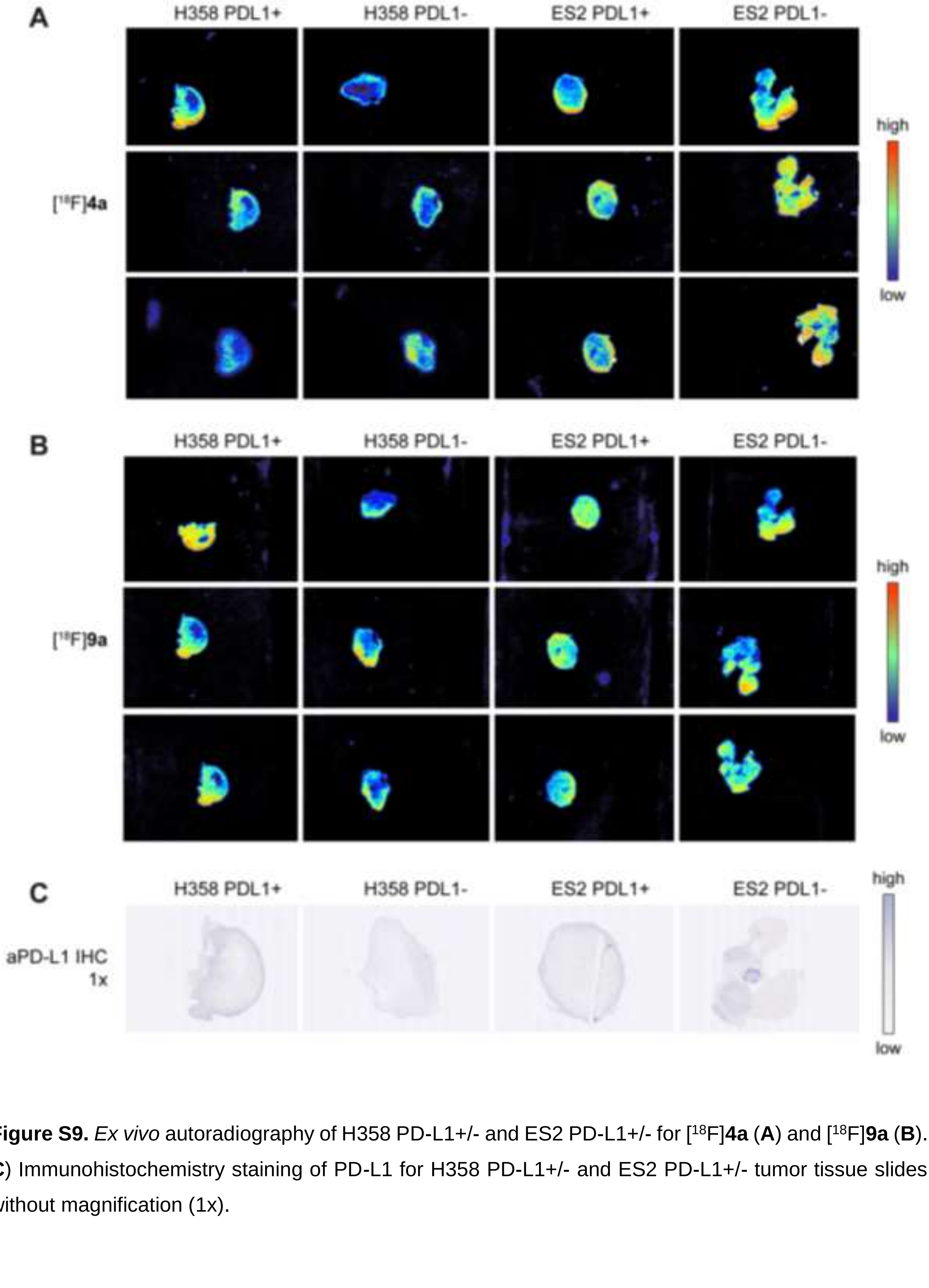

Figure S9. *Ex vivo* autoradiography of H358 PD-L1+/- and ES2 PD-L1+/- for [^{18}F]**4a** (**A**) and [^{18}F]**9a** (**B**). **C**) Immunohistochemistry staining of PD-L1 for H358 PD-L1+/- and ES2 PD-L1+/- tumor tissue slides without magnification (1x).

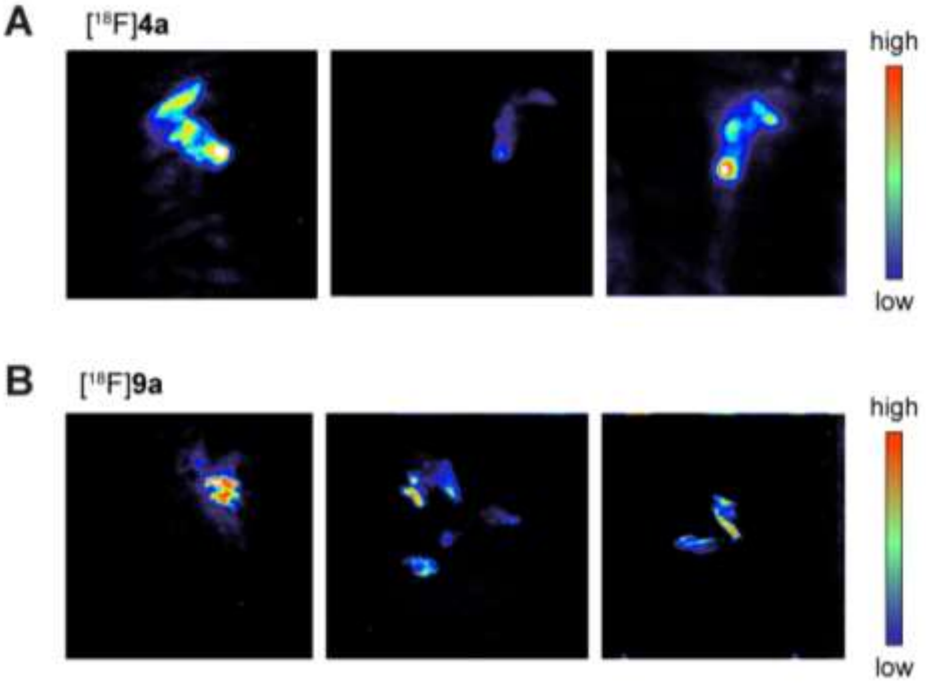

Figure S10. Snap-frozen human tonsils tissue autoradiography with [^{18}F]**4a** (**A**) and [^{18}F]**9a** (**B**) using Typhoon.
